# Electrophysiological biomarkers for foundational signal markers in chronic pancreatitis using machine learning models

**DOI:** 10.3389/fpain.2026.1857591

**Published:** 2026-07-22

**Authors:** Rasmus Bach Nedergaard, Imran Khan Niazi, Fredy Rojas, Usman Ghani, Ana Dugic, Anna Evans Phillips, Mahya Faghih, Misbah Unnisa, Rasmus Hagn-Meincke, Zoltán Hajnády, Dhiraj Yadav, Enrique de-Madaria, Peter Hegyi, Pramod Garg, Rupjyoti Talukdar, Søren Schou Olesen, Shagufta Farheen, Soumya Jagannath, Vikesh Singh, Asbjørn Mohr Drewes

**Affiliations:** 1 Center for Pancreatic Diseases and Mech-Sense, Department of Gastroenterology and Hepatology, Aalborg University Hospital, Aalborg, Denmark; 2 New Zealand College of Chiropractic, Auckland, New Zealand; 3 Heidelberg University, Heidelberg, Germany; 4 Department of Medicine, University of Pittsburgh Medical Center, Pittsburgh, PA, United States; 5 Department of Medicine, Johns Hopkins University, Baltimore, MD, United States; 6 Department of Gastroenterology, Asian Institute of Gastroenterology, Hyderabad, India; 7 Department of Medical Chemistry, Semmelweis University, Budapest, Hungary; 8 Department of Gastroenterology, Hospital General Universitario Dr. Balmis, Alicante, Spain; 9 Department of Gastroenterology, All-India Institute of Medical Science, New Delhi, India

**Keywords:** chronic pancreatitis, cold pressor pain, electrocardiography, electroencephalography, machine learning

## Abstract

**Background:**

Most patients with chronic pancreatitis (CP) experience abdominal pain, but the pathophysiology differs. We aimed to investigate whether using only electroencephalography (EEG) and electrocardiography (ECG) could be used to identify distinct differences between healthy controls and people with CP with and without pain.

**Methods:**

This study utilised cross-sectional data from a multicentre research project, including healthy controls (*n* = 35) and patients with CP (*n* = 124), with (*n* = 94) and without (*n* = 30) pain. EEG and ECG recordings were analysed while resting and in experimental pain states. Analysis was performed using the machine learning models: decision tree classifier, random forest classifier, logistic regression, and support vector machine. The features used in the machine learning models were statistical-, entropy-, and fractal-feature extraction.

**Results:**

Classifying differences between all CP patients and controls yielded 78% F1-score (both EEG and ECG) using a support vector machine. Classifying differences between CP patients with and without pain was less precise, with a maximum 63% F1-score (both EEG and ECG) using a support vector machine. Overall, EEG and ECG improved classifications by up to 10 percentage points. Adding the cold pressor (tonic pain) condition did not reliably change the separation between patients with and without pain, which remained near chance.

**Discussion:**

The classification helps to show the importance of potential EEG and ECG measures in characterization of chronic pancreatitis and pain. Future work should focus on incorporating other disease-specific features in the model.

## Introduction

Chronic pancreatitis (CP) has a prevalence of 50–150 per 100,000 people (1, 2). Pain is the most dominant and debilitating symptom, affecting as many as 70% of patients (3, 4). The underlying pathophysiology of pain is believed to be a complex interaction of chronic inflammation and neural injury that affects peripheral and central pain processing (5–7). As such, painful CP has been associated with altered brain processing through changes in the peripheral (8) and central nervous system (9–11). Magnetic resonance imaging and spectroscopy on patients with CP have shown changes in the brain structure (12), microstructure (13), cortical thickness (14), and hippocampal metabolites (15). Additionally, increased sympathetic activity, derived from venous plasma norepinephrine levels, at a supine venous draw, has been found in patients with pancreatitis and hyperalgesia (16). Such measures, however, need resources and are difficult to use. Therefore, simpler and cheaper methods are needed to assess the neuronal changes. Segments of the pain system in CP can be examined using pancreatic quantitative sensory testing (P-QST), exploring the extent and spread of central sensitization and control mechanisms (17). However, P-QST only evaluates a fraction of the pain response, since pain is also impacted by cognitive and affective components (17). Electroencephalography (EEG) is a simple, non-invasive measure of brain activity that has also been shown to be sensitive to changes in CP patients with pain (18–20). The heart is sensitive to changes in the sympathetic and parasympathetic tone from the autonomic nervous system, which is also influenced by pain (21). Using electrocardiography (ECG) allows for a non-invasive insight into the effects on the activity of the brainstem and the autonomic nervous system. Hence, signals from the brainstem using ECG as a proxy for the autonomic nervous system, and brain activity using EEG can be used to identify distinct pain phenotypes with the potential to use these in personalised treatment. Using continuous EEG and ECG signals allows for a vast number of potential features, both previously established disease specific features and general features representing variability, complexity, and structure for robust machine learning classification. An advantage to general features is also the ability to run analysis in a clinical setting with no specific hardware requirements.

In recent years, artificial intelligence (AI) and machine learning (ML) has enabled early prediction of disease outcomes. In the field of acute pancreatitis, a substantial volume of research using ML/AI has been conducted to predict clinical outcomes (22–31). The ML techniques applied within clinical research tend to favor supervised learning, which improves the outcomes based on previous data. Amongst these models are support vector machines, decision trees, random forest classification, and logistic regression (32). These models vary in complexity, interpretability, and accuracy. However, finding the best model for a given task is often based on trial and error, given the specific patient population and available data.

The initial scope of this paper is to take the parts of EEG and ECG measures, related to the central and peripheral nervous system, using general features to find foundational signal markers. To investigate if foundational signal markers help identify differences comparing healthy controls to people with CP, and within CP patients with and without pain. While the scope of the paper is to investigate which combination of features can help ML distinguish patient groups from healthy controls and pain from pain free CP this is a first step in feature selection to understand the interaction of EEG and ECG, not as a means to replace the clinical classification of chronic pancreatitis, and its subgroups. For the analysis of this paper, EEG and ECG recordings, which were recorded simultaneously in both a resting state and a tonic pain state (the cold pressor test), were used. We hypothesize that machine learning classifiers using EEG and ECG measures can be used to distinguish people with CP from healthy controls. We also hypothesize that the same data and classifiers can be used to distinguish between patients with painful and pain-free CP. The aims of this study were to: (1) Explore which machine learning models best distinguish between healthy controls and patients with CP, and within CP patients with and without pain. (2) Investigate how EEG and EEG combined with ECG perform in the distinction of the groups. (3) Investigate if considering the cortical and autonomic response to painful stimulus (the cold pressor test) will improve the performance of the machine learning models.

## Methods

### Subjects

Data presented in this study were from a multicentre research project (33). Participants were either patients with CP, with or without pain, or healthy controls. The patients with CP were included from research centres in Europe (Alicante, Budapest), the United States (Pittsburgh, Baltimore), and India (Hyderabad, New Delhi) as part of the “INPAIN study” (33).

The healthy controls were recruited through professional networks at the inclusion sites of the research centres (colleagues, hospital staff, and affiliated healthcare professionals at the participating centres). These had no pancreas-associated diseases or painful disorders, and did not receive treatment with analgesics for the last three months. Intermittent use of over-the-counter analgesics was not an exclusion criterion.

Participants with CP fulfilled the M-ANNHEIM classification of definitive CP (34). The exclusion criteria were previous abdominal surgery or severe chronic pain due to diseases other than CP.

All participants gave written informed consent before enrolment. This study complied with the Code of Ethics and complied with all relevant national regulations, institutional policies, and was in accordance with the tenets of the Helsinki Declaration (as amended in 2013), and was approved by the authors' Institutional Review Board or equivalent research ethical committee.

### Data acquisition

A custom-built wearable system based Norteklabs (WIBCI 16T), two Texas Instruments ADS1299 analog-to-digital biopotential converters designed to simultaneously record 9 channels of EEG and 4 channels of ECG signals was used (35). Two periods of recordings were performed (resting state and cold pressor). Common for both recordings was to sit in a comfortable chair with eyes closed and relax as much as possible without falling asleep. For the resting state recording, two minutes of resting state EEG with eyes closed were recorded. Two minutes were used in both the resting and painful stimulation (cold pressor recording), to for one minimise the amount of time the subjects needed to submerge their hand in cold water, and two to keep a short, but consistent window of recorded data between the resting and painful stimulation. Eyes closed was used to minimise eye moving and blinking, reducing the need for manual data cleaning during both resting and painful stimulation. Maintaining the same eye condition for both the resting state and the cold-pressor stimulation also ensured consistency across recordings. For the cold pressor recording (36), the subject was instructed to submerge their hand in cold water at 2 degrees Celsius for up to 120 s. If the subject withdrew their hand from the water earlier, the time was noted, and the recording was stopped.

**Figure 1:**
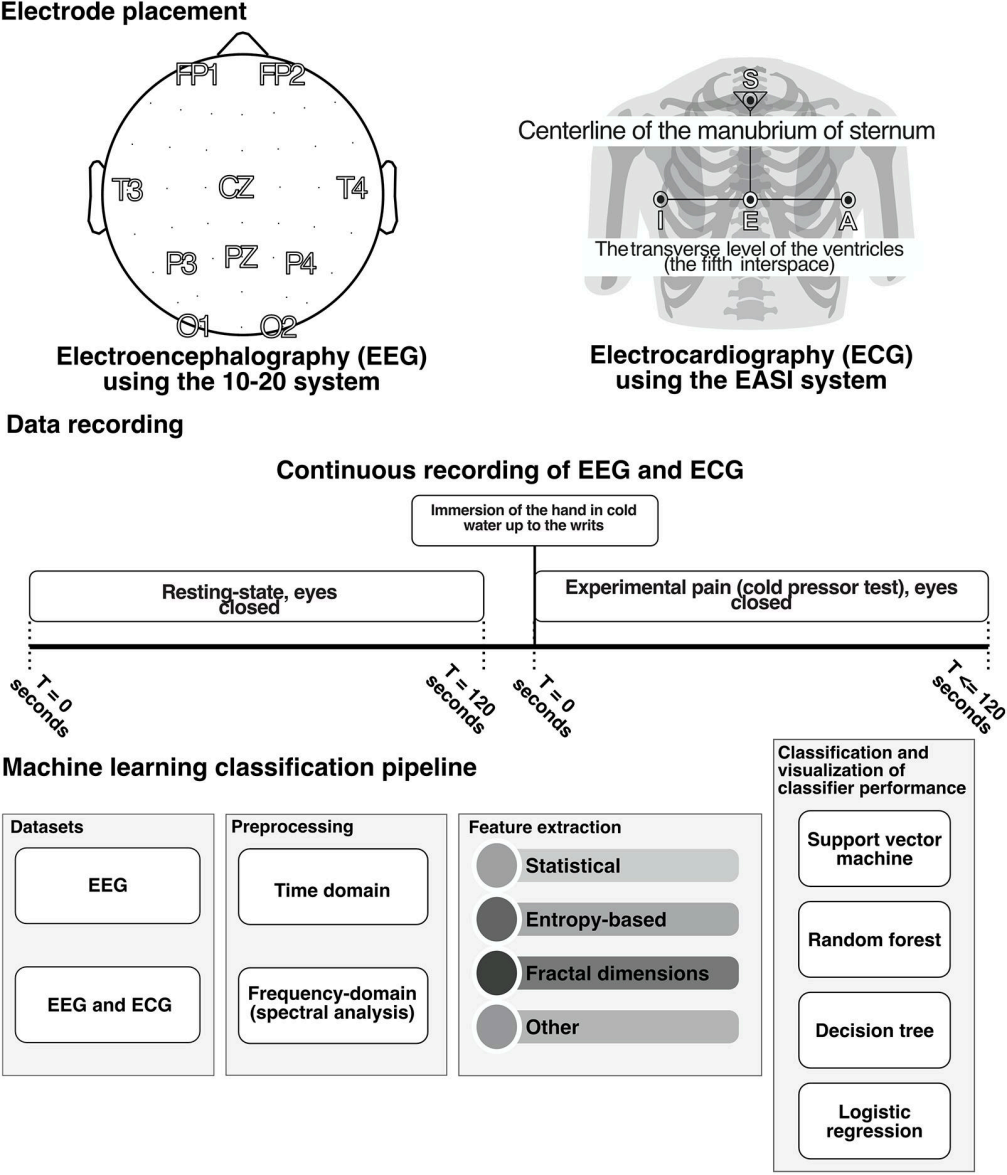
Diagram of electrode placement of electroencephalography (EEG) using the 10–20 system and electrocardiography (ECG) using the EASI system. An overview of the data recording setup, and lastly, a visualisation of the machine learning classification pipeline.

Four ECG Ag/AgCl leads (20 mm Blue Sensor, AMBU A/S, Denmark) were used as electrodes according to the EASI system (37). Three of the ECG leads were placed horizontally along the line of the transverse level of the ventricles (the fifth thoracic interspace), one on the sternum and one on each side of the sternum on the midaxillary line. The last electrode was placed on the centre of the manubrium sternum. The EEG channels (FP1, FP2, Cz, T3, T4, P3, Pz, P4, O1, and O2) were placed according to the 10–20 system using only the 9 mentioned channels in a 19-channel sintered EEG Electrode Cap (OpenBCI: Brooklyn, NY). A diagram of electrode placements is available in Figure 1. The EEG and ECG data were collected with a sampling frequency of 250 Hz and saved for later offline data processing.

### Preprocessing

**Figure 2:**
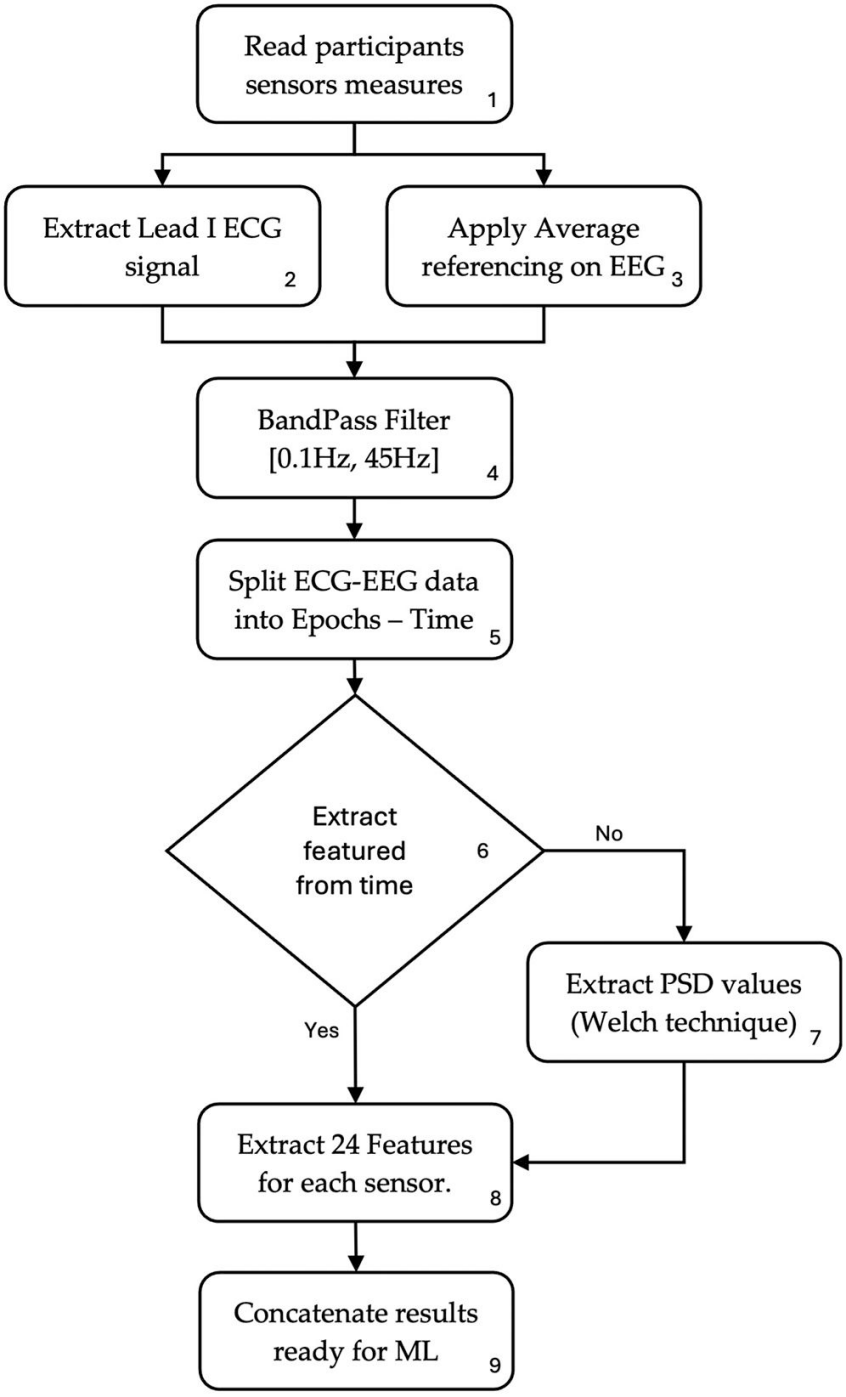
Preprocessing pipeline producing ML-ready features.

As illustrated in Figure 2 (steps 1–5), the MNE Python library was used for EEG and ECG preprocessing. Non-EEG channels were excluded from the EEG analysis. The data were average referenced before applying a bandpass filter between 0.1 and 45 Hz. After this, the continuous EEG signals were segmented into 3-second epochs, incorporating a 1-second overlap for each epoch. For the ECG analysis, data were obtained using a 1-lead configuration; the signal was obtained by subtracting the potential measurements (µV) of the right side electrode (A) from the left side electrode (I), resulting in a lead I. These data were also band-pass filtered between 0.1 and 45 Hz, and segmented into 3-second epochs, incorporating a 1-second overlap for each epoch.

## Time and frequency domain representations

After creating ECG and EEG segments (Figure 2, step 5), two parallel processing routes were followed for feature extraction.

Time domain route (Figure 2 step 5 → step 8): The EEG and ECG segmentation step (step 5) already produces epochs in the time domain representation. Therefore, no additional preprocessing was required before feature extraction. This direct path is reflected in Figure 2 by the arrow connecting step 5 directly to step 8 (feature extraction).

Frequency domain route (Figure 2 step 7): PSD was estimated using Welch's method on 3 s EEG epochs sampled at 250 Hz (59). The segment length was set to the full epoch duration of 750 samples, with no Welch window overlap and an FFT length of 1,000 samples, resulting in a frequency-bin spacing of 0.25 Hz. Frequencies between 0.1 and 45 Hz were retained for analysis, yielding 180 PSD values per sensor per epoch. These PSD values were then used to extract the features described in the subsequent subsection.

### Feature extraction

The previous section described two representations of each epoch: time domain and frequency domain (PSD). For each representation, the same set of 24 features was extracted, as illustrated in Figure 2 (step 8). A brief high-level description of these 24 features is provided below.

1. **Statistical:** Included are *mean*, *variance*, *standard deviation*, *peak-to-peak amplitude*, *minimum* and *maximum* values along with their indices, *root mean square*, *kurtosis*, and *skewness* (38, 39). In addition, two Hjorth parameters, *mobility* and *complexity*, were used. *Hjorth mobility*, defined as the square root of the variance of the signal's first derivative, essentially indicates the signal's mean frequency. *Hjorth complexity*, on the other hand, is the ratio of the mobility of the signal's derivative to the mobility of the signal (40).
2. **Entropy-based:** *Approximate-* and *sample-entropy* (41, 42) were extracted from the dataset, and both are indicators of signal regularity, persistence, and complexity. To capture dynamics not observed by these measures, *permutation-entropy* was also extracted (40), offering a refined view of the 'signal's temporal structure and transitions. *Spectral entropy* was calculated to analyse power irregularity across frequencies (42), which involved normalising the spectral power within a specific frequency range and then computing Shannon entropy from this normalised power distribution. Similarly, *SVD-entropy* was derived, aligning with the principles of Spectral Entropy but employing singular value decomposition (40).
3. **Fractal dimensions:** These features quantify the complexity, randomness, and chaotic nature of EEG signals by analysing fractal characteristics. Fractal dimensions are particularly effective in capturing the dimensional complexity and self-similarity in non-stationary and nonlinear EEG signals. Techniques such as the Petrosian, Higuchi (43), and Katz Fractal Dimensions (44) have been employed for this, enhancing the machine learning models with a detailed understanding of fractal properties of the EEG data.
4. **Other:** Complementing the features previously described, our classification approach also incorporates *Curve Length* (44) and *Zero Crossing* (38). Curve Length acts as a direct measure of the EEG signal's variability or energy, aiding in the identification of high-activity periods or fluctuations. Zero Crossing efficiently captures the rate of signal change, facilitating the differentiation of various neural activities.

Feature scaling was implemented using scikit-learn's Pipeline with standardization applied within each cross-validation fold. Specifically, the mean and standard deviation were estimated only from the training partition of each fold and then used to transform the corresponding training and validation partitions. This procedure was applied during GridSearch-based hyperparameter optimization and repeated KfoldCV, ensuring that information from the validation folds was not used when fitting the scaling parameters. Therefore, the scaling procedure was nested within the cross-validation workflow and helped prevent feature leakage during model selection. The complete set of 24 features, with definitions and domain, is listed in Supplementary File 1.

### Classification

In the classification process, machine learning classifiers were trained and evaluated for their ability to interpret time-domain and frequency-domain data (spectral analysis). Multiple classifiers were used to try to understand which models perform the best using the available data. Specifically both decision tree and random forest was used to see if a simpler model, that can also be used to visualise the decision path (decision tree) would perform similarly to a more complex model (random forest).

## Balancing strategy

Our current dataset has two levels of imbalance: first, the number of signal segments (epochs) per subject, and second, the number of subjects per class. In this study, we implemented a custom preprocessing pipeline that applies the oversampling technique SMOTE independently to each subject. For each individual, a subject-specific dataset was created before applying SMOTE, guaranteeing that each new synthetic sample was generated only from neighbouring samples belonging to the same individual. Once the oversampling process was completed, each individual had the same number of epochs. However, since each subject represents one class {0,1}, another source of imbalance remains due to the different number of subjects per class. To address this second level of imbalance (uneven subject counts per class), we relied on the class_weight parameter available in all selected scikit-learn ML algorithms (see Table 1 for specific settings per algorithm). This parameter adjusts loss functions to penalize misclassifications of the minority class 0 more heavily, effectively balancing class influence at training time without requiring additional resampling.

**Table 1:** All combinations of the listed values were exhaustively evaluated.

Model	Hyperparameter	Values
Decision Tree (DT)	criterion	gini, entropy
	max_features	sqrt, log2
	max_depth	2 to 19 (integer values)
	splitter	best, random
	class_weight	balanced
	random_state	0, 42
Random Forest (RF)	n_estimators	25, 50, 75, 100, 150, 200
	criterion	gini, entropy, log_loss
	max_features	sqrt, log2
	max_depth	None, 2, 10, 20
	class_weight	balanced
	random_state	0, 42
Logistic Regression (LR)	penalty	l1, l2, elasticnet, none
	C	0.01, 0.05, 0.1, 0.5, 1, 2, 3, 4, 5, 8, 10, 12, 15
	solver	saga
	max_iter	500
	class_weight	balanced
	random_state	0, 42
	l1_ratio	0.0, 0.25, 0.5, 0.75, 1.0
Support Vector Machine (SVM)	C	0.01, 0.05, 0.1, 0.5, 1, 2, 3, 4, 5
	kernel	linear, poly, rbf, sigmoid
	probability	TRUE
	class_weight	balanced
	random_state	0, 42

Performance outside this space was not tested.

## Hyperparameter optimization

Hyperparameter optimization was conducted using Grid Search with cross-validation to identify the optimal combination of parameters, with the F1-score serving as the primary optimization criterion (63). Grid Search provides a systematic and exhaustive approach to parameter tuning, ensuring comprehensive exploration of all parameter combinations defined in Table 1 (60). To improve the reliability of performance estimation for each parameter configuration, our Grid Search methodology incorporated a custom 5-fold cross-validation (5kfoldCV) strategy. This approach helps reduce the risk of selecting hyperparameters that overfit a specific data partition, promoting more stable internal performance estimation across different training splits.

## Custom subject-wise K fold CV

Due to subject-level labelling (all epochs of a subject share the same label), small dataset size, and class imbalance (majority class = 1), we adopted 5-fold cross-validation instead of a single train/test split to obtain more stable performance estimates. Standard GroupKFold ensures that all epochs of a subject stay together, but it does not balance the count of subjects per class across folds. We therefore implemented a custom function that: (1) performs subject-wise splitting, (2) balances the number of subjects per class within each fold, and (3) excludes all SMOTE-generated synthetic data from the validation set (validation uses only original real samples). This design prevents data leakage and reduces overestimation risk.

## Model selection and model evaluation

The analysis followed a two-stage design. In the first stage, model selection, hyperparameters for each algorithm family were chosen by Grid Search with the custom subject-wise 5-fold cross-validation described above, using F1 as the selection criterion. In the second stage, model evaluation, the single configuration selected for each condition was assessed for stability using 1,000 repeated subject-wise 5-fold cross-validation, each repeat using a different subject-level partition. Performance across the 1,000 repeats is summarised by the mean, standard deviation, and 2.5 to 97.5 percentile interval for accuracy, precision, recall, and F1. The Grid Search results, therefore, report the selected configurations, and the repeated cross-validation results report how those configurations behave across subject splits drawn from a population assumed similar to the present sample.

### Machine learning classifiers

In this study, there are two classification tasks. The first task is to identify whether an epoch (signal segment) originated from a CP patient or from a Control patient. The second task is to identify whether the epoch originated from a painful CP patient or from a pain-free CP patient.

For each classification task, two sensor conditions were used: (1) EEG data only, and (2) combined EEG + ECG data. Additionally, each model was trained independently for the resting condition and the cold-pressor recording condition. As mentioned in the Balancing Strategy section, we used ML algorithms that support automatic class balancing through the class_weight parameter for this exploratory study. Below is a brief description of each ML algorithm family:

1. **Decision tree classifier (DT)** provides a graph-based representation of possible solutions by starting from a tree node and ending at a leaf node. The parameters used to tune the tree were three hyperparameters: max_features, criterion, and max_depth.
2. **Random forest classifier (RF)** is an ensemble learning method that utilises multiple decision trees to make predictions. It combines the output of these trees to reach a single result, improving accuracy and reducing overfitting compared to individual decision trees.
3. **Logistic regression (LR)** is a parametric and straightforward model that uses a sigmoid function to predict the probability of event occurrence. The only hyperparameter used is C, which is a regularisation factor.
4. **Support vector machine (SVM)** is a machine learning algorithm based on finding the hyperplane that optimally divides a dataset into classes. SVMs can utilise the kernel trick technique to solve nonlinear classification problems. The kernel trick involves mapping the input features into high-dimensional feature spaces where linear separation is possible. This enables the SVM to form nonlinear decision boundaries in the original feature space.

### Model comparison

All data are reported in an F1 score, which is calculated using the following formula:

$$F1\;\mathrm{score}=\frac{\mathrm{True}\;\mathrm{positive}}{\mathrm{True}\;\mathrm{positive}+\frac{1}{2}(\mathrm{False}\;\mathrm{positive}+\mathrm{False}\;\mathrm{negative})}\;\;100$$

This measure of accuracy was chosen to help assess performance with imbalanced classes (45).

### Statistical analysis

To investigate whether the groups differed in how long subjects kept their hand in the water during the cold pressor test, a Cox proportional hazards regression was used, with time to hand withdrawal (0–120 s) as the outcome and group (healthy control, patients with pain, and patients without pain) as the predictor. Participants who tolerated the full 120 s were treated as censored observations. To compare model performance across configurations, the repeated cross-validation F1 distributions were compared with a Friedman test, with Kendall's W reported as the effect size to gauge the practical magnitude of any difference. The level of significance was set to 0.05.

### Feature importance

Feature importance analysis was conducted using both impurity-based and permutation-based approaches. Initially, impurity-based feature importance was evaluated using the best-performing tree-based model, either Decision Tree (DT) or Random Forest (RF), since these methods provide native split-based importance estimation. This analysis was performed for each signal recording condition (resting or cold pressor), considering features derived from both time and frequency domains using either all sensors (EEG + ECG) or EEG-only configurations. However, impurity-based importance is known to be sensitive to continuous variables and may introduce bias toward features with a larger number of possible split points. Additionally, the 5-fold cross-validation procedure showed performance variability, likely associated with the limited dataset size. Therefore, to further assess the consistency of feature relevance across different attribution approaches, permutation-based feature importance was subsequently applied to the previously selected tree-based models.

## Impurity-based feature importance

Feature importance was calculated from the training set based on how much each feature contributed to reducing impurity across the decision branches of the model during training (61, 62) The importance score accumulates the impurity reduction associated with each feature over all splits and trees. Features with higher scores are interpreted as having a greater contribution to the model's decision-making process during training.

## Permutation-based feature importance

Permutation-based feature importance was evaluated on the test set. In this study, permutation-based feature importance was only applied to the selected tree-based models (DT and RF) from impurity-based feature importance. The method first computes a baseline performance metric, which in this study was the f1-score. Then, one feature at a time is randomly permuted across samples while the remaining features are kept unchanged, and model performance is recalculated. The reduction in performance relative to the baseline is used to estimate feature importance. Features producing a larger decrease in performance are interpreted as having a greater contribution to the model's predictions.

All analyses were performed in Python 3.8.8 using numpy 1.24.4, pandas 1.5.3, scikit-learn 1.3.2, matplotlib 3.7.0, seaborn 0.13.1, MNE 1.3.1, imbalanced-learn 0.12.2, and antropy 0.1.5; the full environment is provided as a requirements file in the Supplementary Material.

## Results

Table 2 presents the demographics of participants. All data are presented in features in the time and frequency domain based on the extracted features, and all participants were subjected to both a resting-state and an experimental tonic pain state. All F1-scores are presented as the average of 5-fold cross validation, and reported with mean and range of the 5-fold cross validation.

**Table 2:** Demographics of the people included in the study are presented in mean (SD).

Measure	Healthy controls (*n* = 35)	Chronic pancreatitis (*n* = 124)	*P*-value
Males, *n* (%)	17 (48.57)	86 (69.35)	**0.028**
Mean age (SD)	42.5 (15)	38.2 (13.8)	0.1
Age group, *n* (%)			
<40	20 (57.14)	76 (61.29)	
40–60	9 (25.71)	35 (28.23)	
>60	6 (17.14)	13 (10.48)	
BMI, kg/m^2^ (SD)	25.6 (4.2)	21.4 (5.3)	**<0.001**
Race, *n* (%)			**<0.001**
Asian	16 (45.71)	106 (85.48)	
White	17 (48.57)	18 (14.52)	
African	1 (2.86)	–	
Other	1 (2.86)	–	

Bold represents statistical significant values.

The following results correspond to the model selection stage and report the configuration selected for each condition by Grid Search. The full fold-wise performance ranges for these selected configurations are provided in Supplementary File 2.

### Resting-state

#### Classifying patients vs. controls

**Figure 3:**
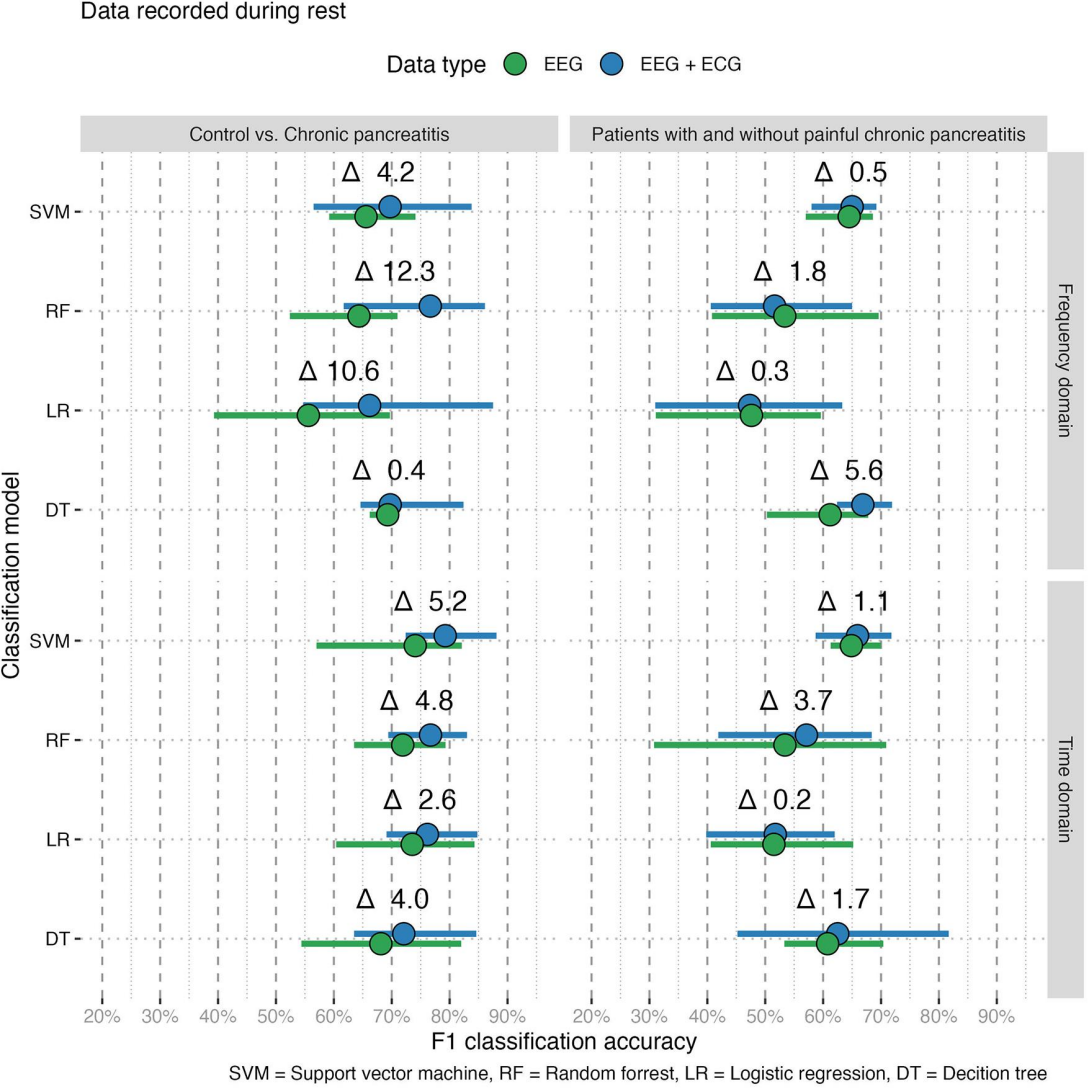
Resting state recorded data and the corresponding F1-scores for the machine learning models: support vector machine (SVM), random forest (RF), logistic regression (LR), and decision trees (DT). The two analysed groups are healthy controls compared to patients with CP and patients with CP with and without pain. Additionally, the data are divided into a time domain and a frequency domain component. The two data types [EEG features alone (green), and EEG and ECG features (blue) are visualised along with the absolute percentage point differences denoted by the Δ].

When comparing healthy controls to all patients with CP in the time domain, EEG features were first used. The support vector machine classifier performed best, with an average F1 score of 74% (range 57%–82%). Adding ECG features to the model increased the F1 score of the best models, and the support vector machine reported an average F1 score of 79% (range 72%–88%), Figure 3.

In the frequency domain using only EEG features, the best classifier was the random forest classifier, which had an average F1 score of 64% (range 52%–71%). After adding ECG, the random forest classifier reported an average F1 score of 77% (range 62%–86%).

#### Classifying patients with and without pain

Across patients with and without pain, the decision tree classifier, using only EEG in the time domain, had an average F1 score of 60% (range 53–70). After adding ECG features to the model, the decision tree classifier had an average F1 score of 63% (range 45%–81%).

In the frequency domain, using only EEG, the decision tree classifier had an average F1 score of 61% (range 50%–68%). Adding ECG features to the model yielded a decision tree classifier with an average F1 score of 67% (range 62%–72%).

All relevant data on the resting-state analysis are available in Figure 3.

### Experimental tonic pain

Overall, the time the groups could keep their hand in cold water differed significantly across conditions (Cox regression: *χ*^2^ = 37.95, df = 2, *p* < 0.001). Due to right-censoring at 120 s (42% of participants reached maximum tolerance). Patients without pain endured a median of 105 s, and patients with pain endured a median of 50 s. Cox proportional hazards regression revealed that, compared to healthy controls, patients without pain had 4.14 times higher hazard of early withdrawal [HR = 4.14, 95% CI (1.62, 10.58), *p* = 0.003], and patients with pain had 7.41 times higher hazard of early withdrawal [HR = 7.41, 95% CI (3.21, 17.11), *p* < 0.001]. Among patients, those with pain showed significantly lower tolerance than pain-free individuals, with 1.79 times higher hazard of early withdrawal [HR = 1.79, 95% CI (1.04, 3.08), *p* = 0.037].

#### Classifying patients vs. controls

**Figure 4:**
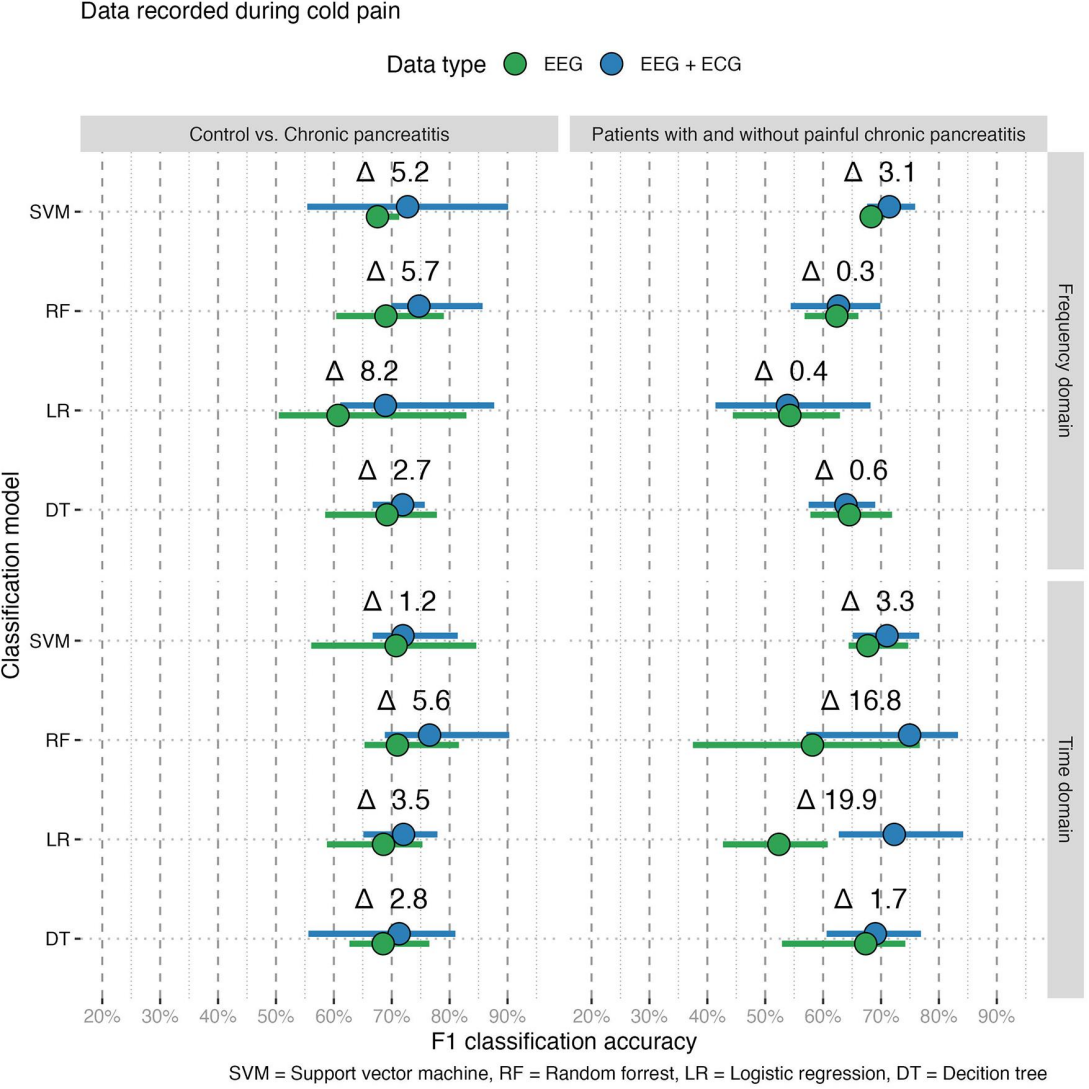
Cold pressor pain recorded data and the corresponding F1-scores for the machine learning models: support vector machine (SVM), random forest (RF), logistic regression (LR), and decision trees (DT). The two analysed groups are healthy controls compared to patients with CP and patients with CP with and without pain. Additionally, the data are divided into a time domain and a frequency domain component. The two data types [EEG features alone (green), and EEG and ECG features (blue) are visualised along with the absolute percentage point differences denoted by the Δ].

Comparing healthy controls and patients with CP during the cold pressor test using only EEG features in the time domain, the random forest classifier has the highest F1 score with an average of 71% (range 65%–82%). After adding ECG, the random forest classifier showed an average F1 score of 77% (69%–90%), Figure 4.

In the frequency domain, the random forest classifier had an average F1 score of 69% (range 60%–79%). Adding ECG, the random forest classifier resulted in an average F1 score of 75% (70%–86%).

#### Classifying patients with and without pain

In the time domain, using only EEG features, the support vector machine had an average F1 score of 68% (range 67%–70%). After adding ECG features, the decision tree classifier showed the highest accuracies, with an average F1 score of 74% (range 57%–83%).

In the frequency domain using only EEG, the support vector machine was the best-performing classifier, with an average F1 score of 68% (range 67%–71%). Adding ECG data to the classifiers, the support vector machine classifier had an average score of 71% (65%–77%). All relevant data on the resting-state analysis are shown in Figure 4.

### Model evaluation

The selected configurations were re-evaluated across 1,000 repeated subject-wise 5-fold cross-validation partitions. The highest observed F1 was obtained with EEG and ECG in the time domain at rest using a support vector machine (mean F1 78%, 95% CI: 65 to 90; accuracy 80%, 95% CI: 70 to 90). Across conditions, adding ECG to EEG increased the mean F1 by about 5–10 percentage points (Table 3). For the painful vs. pain-free task, the same evaluation showed accuracy near chance (49% to 58%) with wide confidence intervals; the higher F1 values in this task were driven by recall on the majority class rather than by class separation, as shown by precision near 50% (Table 4). The only configuration above its resting counterpart was EEG and ECG under the cold pressor, though its interval still included chance. Per configuration distributions for all sixteen models are provided in Supplementary File 3. A Friedman test across the repeated cross-validation F1 distributions was significant (*p* < 0.001), but the effect size was small (Kendall's W = 0.17) and the distributions overlapped, so no single configuration was treated as best. These statistical comparisons should be interpreted as exploratory evidence, because the independence assumption may be weakened under repeated k-fold cross-validation.

**Table 3:** Control vs. CP, repeated cross validation [mean (95% CI)].

Sensors	Domain	Condition	Model	F1%	Accuracy %	Precision %	Recall %
EEG + ECG	Time	Resting	SVM	78 [65, 90]	80 [70, 90]	85	72
EEG + ECG	Time	Cold pressor	RF	74 [62, 85]	75 [65, 85]	79	70
EEG + ECG	Frequency	Resting	RF	77 [65, 87]	78 [68, 87]	82	73
EEG + ECG	Frequency	Cold pressor	RF	72 [58, 83]	74 [63, 83]	78	66
EEG	Time	Resting	SVM	71 [58, 83]	72 [60, 83]	73	69
EEG	Time	Cold pressor	RF	68 [55, 80]	71 [60, 80]	74	64
EEG	Frequency	Resting	RF	67 [52, 80]	70 [58, 80]	72	64
EEG	Frequency	Cold pressor	RF	64 [49, 76]	67 [57, 78]	72	58

**Table 4:** Painful vs. pain free CP, repeated cross validation (mean [95% CI]).

Sensors	Domain	Condition	Model	F1%	Accuracy %	Precision %	Recall %
EEG + ECG	Time	Resting	DT	58 [43, 70]	56 [42, 68]	55	61
EEG + ECG	Time	Cold pressor	SVM	63 [47, 73]	58 [45, 68]	57	72
EEG + ECG	Frequency	Resting	DT	57 [34, 70]	51 [38, 62]	50	69
EEG + ECG	Frequency	Cold pressor	DT	48 [32, 63]	50 [37, 63]	50	47
EEG	Time	Resting	DT	60 [43, 72]	55 [43, 68]	54	69
EEG	Time	Cold pressor	SVM	61 [43, 70]	51 [38, 60]	50	80
EEG	Frequency	Resting	DT	59 [42, 70]	54 [42, 65]	53	68
EEG	Frequency	Cold pressor	SVM	54 [27, 69]	49 [40, 58]	49	65

### Subsection: interpretability results

In this study, two main classification tasks were evaluated: distinguishing Chronic Pancreatitis (CP) patients from Healthy Controls, and distinguishing painful CP from pain-free CP. Each task was assessed under multiple experimental conditions depending on: (1) the signal recording condition (resting or cold pressor), (2) whether the features were derived from the time or frequency domain, and (3) whether the models were trained using EEG-only signals or the full sensor configuration (EEG + ECG). Therefore, each classification task included eight independent training conditions in which the machine learning algorithms were trained and evaluated separately. In this section, we present the feature importance analysis for the best-performing condition of each task. The feature importance results for all evaluated conditions are provided in Supplementary File 4.

### CP vs. control time domain EEG + ECG

**Figure 5:**
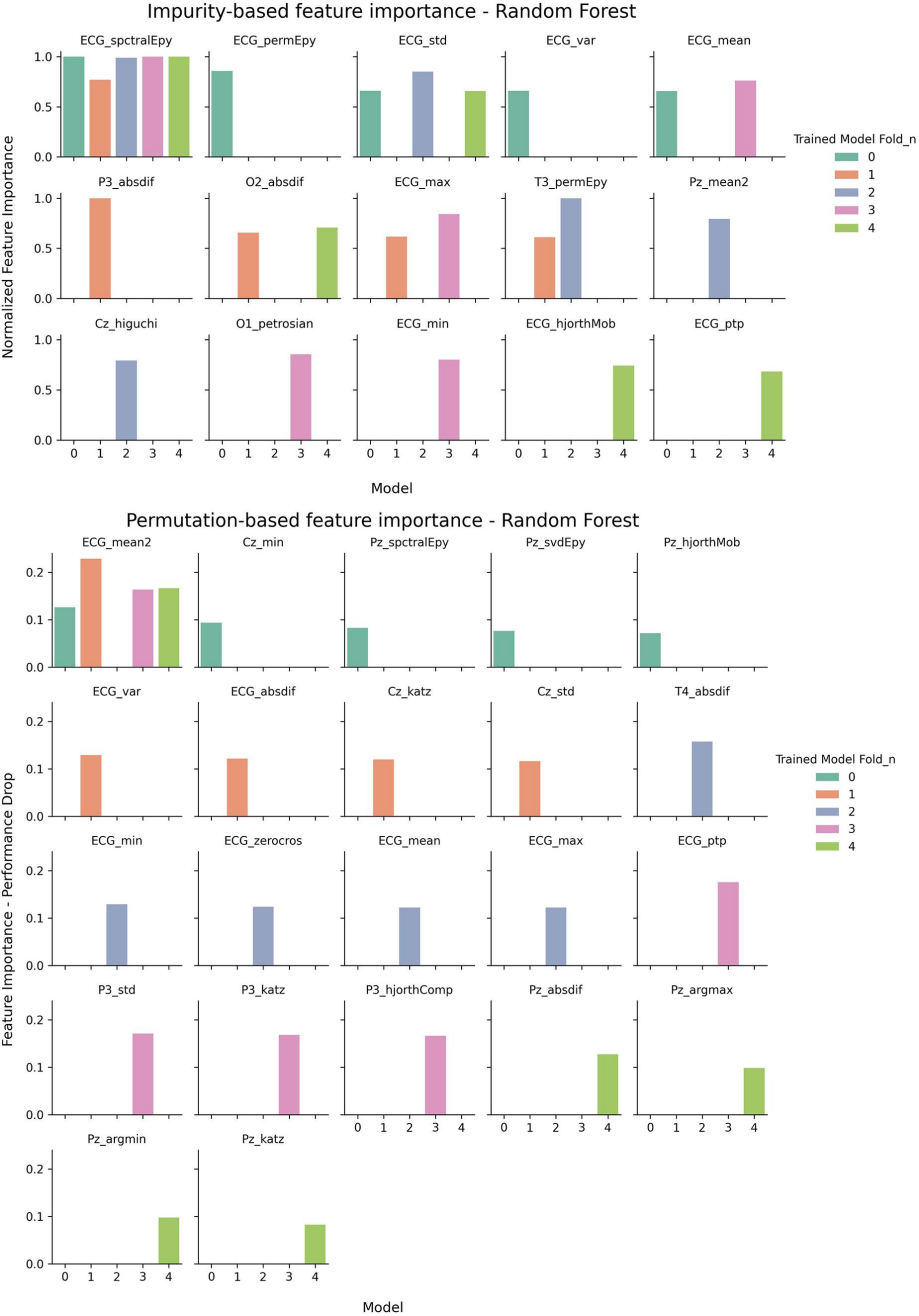
Feature importance analysis for impurity and permutation-based approach for classification task, CP vs. Control.

Figure 5 presents the feature importance results for the Decision Tree model. In the impurity-based feature importance analysis, a feature derived from ECG signals (Spectral Entropy) appeared to play a relevant role in reducing impurity during training, suggesting that the model frequently used this feature to learn patterns distinguishing CP patients from Healthy Controls. In the permutation-based feature importance analysis, the trained fold models again showed some agreement regarding the relevance of ECG-derived features. However, in this case, the ECG mean across epochs appeared to produce a larger impact on validation performance when permuted, suggesting a greater contribution to the predictive task.

### Painful vs. pain-free CP time domain cold pressor EEG + ECG

**Figure 6:**
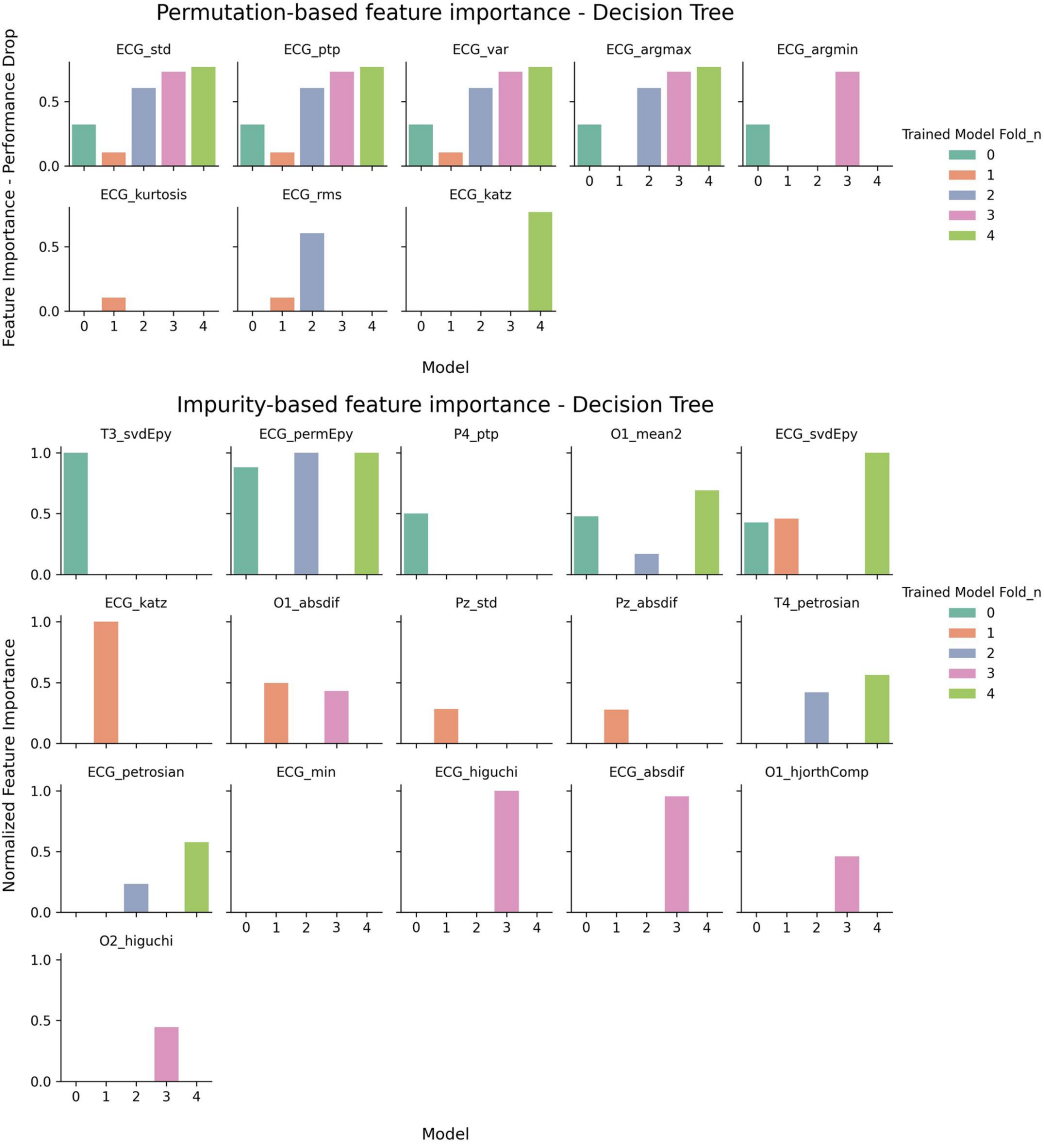
Feature importance analysis for the impurity and permutation-based approach for the classification task, painful CP vs. Pain-free CP.

Figure 6 presents the feature importance results for the Decision Tree model. Although there was variability in the top-5 ranked features across the five trained fold models, some agreement was observed for ECG-derived features, particularly Permutation Entropy and SVD Entropy. Additionally, some consistency was observed for the EEG O1 channel mean square feature. When the model was further evaluated using permutation-based feature importance, which measures the reduction in performance after feature shuffling at the sample level, a stronger agreement emerged regarding the relevance of ECG-derived features such as standard deviation, peak-to-peak amplitude, and variance.

Supplementary File 4 (Feature Importance.pdf) contains a figure for comprehensive feature analysis.

## Discussion

Classifying differences between healthy subjects and patients with CP yielded results in the 60%–79% range. Classifying differences between patients with CP with and without pain had slightly lower results. Overall, no single machine learning model outperformed any other model, but adding ECG data to a model based on EEG alone improved the performance. Adding a tonic pain stimulus helped distinguish between patients with and without pain, but not between the healthy controls and patients with pain. The reason for the ladder finding is likely that many factors other than pain influence the EEG and ECG in the patients (15, 46). This foundational analysis allows for the detection of pancreatic pain well above chance. Although this cannot describe all differences between disease states, EEG and ECG features can be used as part of a comprehensive model of the pain phenotype, describing important components of pancreatic pain.

The repeated cross validation showed that the painful vs. pain free contrast was unstable across subject level partitions, with accuracy near chance and wide confidence intervals. These results should be read as exploratory. The configuration combining EEG, ECG, and the cold pressor was the only one to exceed its resting counterpart, which is consistent with autonomic response to tonic pain carrying pain relevant information, but the present features and sample size do not support a reliable pain classifier. For the same reason, feature importance for the pain contrast is reported with caution, as importance rankings are unlikely to be stable when classification itself is unstable.

### Building a model to explain pain in chronic pancreatitis

Electroencephalography has been used extensively in the detection and classification of CP (9, 10, 47). It has investigated the involvement of neuropathic pain (10) and contributed to the understanding of central mechanisms underlying chronic pancreatic pain (47). ECG was included as a feature in this paper to gain insights into different aspects of the autonomic nervous system, as e.g., higher parasympathetic activity predicts decreased pain (21). Combining EEG and ECG in almost all instances increased the performance of the machine learning classification, especially between patients with and without pain. In the feature extraction for this analysis, no specific ECG-derived parameters were extracted, the same four feature groups described in feature extraction were used. However, some of the variability of the ECG-derived parameters was encompassed in the statistical, entropy-based, and fractal dimensions of the feature extraction. Both the time and frequency domain components of the analysis performed well above chance.

The patient population differs from the healthy controls in the presence of pain (for most patients), but also for other disease characteristics. Some of these effects are malnutrition, lack of vitamins and micronutrients, fatigue, co-medication, concomitant diseases, and low-grade inflammation. In contrast, patients with and without pain are more comparable. A combination of EEG and ECG features, combined with a painful stimulus, showed the strongest differentiation between pain stages within CP, likely as the autonomic response to pain is a crucial dimension of the pain response.

ECG is widely used in the classification of pain, and a recent review found that parasympathetic activity measures can be used as a pain predictor (21). The addition of ECG adds valuable insights, potentially to the pain system, which possibly results in improved accuracy between healthy patients and between painful and pain-free CP. The current paper strengthens the importance of brain networks and brainstem factors in the distinction of pancreatic pain.

The choice to generate a model with predictive performance, using five-fold cross validation makes any model interpretability difficult, since each fold potentially generates a new set of feature importances. Hence five-fold validation was used instead of any one particular training split.

### The effect of experimental pain stimulation

The use of tonic pain, the cold pressor test, is one of the best experimental measures to mimic clinical pain (48, 64). Thus, adding a tonic pain stimulus may reduce the differences between the healthy controls and patients. However, this did not improve classification accuracies between healthy controls and patients with CP, likely as many other factors than pain interfere with the electrophysiological recordings. Within patients with CP, the classification performance distinguishing painful and pain-free states improved when using both the EEG and the ECG features. This is likely due to factors that characterise chronic pain, such as neuronal hyperexcitability, reorganisation of pain networks, and malfunctioning of the autonomic nervous response and control mechanisms. Hence, a pain stimulation will trigger these abnormal circuits, reflected in changes in the EEG and ECG in patients with preexisting pain. In contrast, the central pathways in patients without pain behave differently. Thusly, adding pain as a stimulus might help differentiate the heterogeneity between patients with pain within the pain population.

### Machine learning models

The choice to use artificial intelligence models, specifically machine learning models, was to integrate the large amount of information available in EEG and ECG data into a single model. The aim was to identify correlations in the dataset for which exploratory analysis would be too exhaustive, allowing us to avoid being bound by assumptions attached to conventional statistical methodology.

Out of all the tested models, there was no clear best-performing classifier in all tests. However, the support vector machine had the highest overall result. The random forest and decision tree classifiers outperform the support vector machine in some subcategories.

The machine learning models were generally better at distinguishing healthy controls from patients with CP than they were at distinguishing patients with CP with and without pain. This is likely due to the heterogeneity of the CP population compared to a more homogenous control group. The logistic regression in this setup was inferior to the other models. Logistic regression is a widely used model within the medical field, but it assumes a linear relationship between the features and the outcome (49). As EEG and ECG are nonlinear signals (50), this might explain why the support vector machines and random forest classifications performed better since they were able to capture nonlinearity and perform well in a complex feature space (51).

### Feature selection

The four groups of features were not selected specifically for CP. They were selected for robustness and reproducibility in electrophysiological signals. Additional features, other than those extracted from EEG and ECG, may increase the accuracy of the phenotyping. Research on acute pancreatitis has, among other predictors, yielded predictions of severity and complications. These models used artificial neural networks, decision tree classifiers, logistic regressions, and support vector machines, as well as machine learning models (22–31). Accuracies between 72 and 93 percent were reported using a combination of clinical and biochemical measures. The higher performances are likely due to the use of features that are very specific to acute pancreatitis, where the initial features used in this paper were chosen to be robust across diseases, and able to extract in any computational environment (not reliant on specific hardware). A use of more disease-specific features in future work is warranted. Apart from questionnaire data, magnetic resonance imaging of the pancreas may be useful as it has been used for fibrosis detection (52), inflammation detection (53), and changes in pancreatic duct morphology (54). Within serum biomarkers, TGF-β, the IL-6 receptors, and genetic markers have been shown to associate with pain and CP, and could also be used (55). However, these methods are cumbersome and expensive and cannot be used at bedside examinations.

### Feature importance

The variability observed in the top-ranked features across folds suggests that feature attribution may be sensitive to the training subset composition. This behaviour is expected in small datasets, where minor sampling differences can substantially affect learned decision boundaries and associated feature rankings. Therefore, the identified important features should not be interpreted as definitive biomarkers or disease-specific indicators, but rather as features that the models found useful under the current experimental conditions.

### Strengths and limitations

Among the strengths in the current study are the addition of ECG, which improved the machine learning models in classifying patients with and without pain, independent of the cold pressor endurance. The usability of EEG and ECG features helps strengthen the foundational knowledge of the physiology of CP. Using robust features and incorporating ECG measures significantly improved the classification of the described models. There are several limitations. The distribution of participants from different races varies across the control and patient populations, which may cause bias in the computations. There is also an imbalance in the number of people who have CP with pain (*n* = 94) and without pain (*n* = 30), which might have skewed the results, this is also the reason why a leave one site out strategy was not used in the validation of the results. Finally, the amount of time each subject group could endure the cold pressor test differed. This has been reported previously and was attributed to hyperalgesia (6, 8, 56). However, the lowest average endurance was 66 s for patients with pancreatic pain. This has previously been shown to be sufficient to induce expected changes in EEG (57).

## Conclusion

This work illuminates the possibility of using features from EEG and ECG to build a phenotype to better understand the pathophysiology of CP. For future work within this field, we recommend using EEG and ECG as features in machine learning classification and adding more disease-specific features. The addition of a tonic pain stimulus will likely yield better results than resting stage recordings alone. In the overall selection of classification algorithms, no algorithm performs best, and we recommend a mix of multiple methods to find the best for any given task. Additionally, the use of ensembled classifications has previously also resulted in better test accuracies and may be helpful (58).

### New and noteworthy

This study combines EEG and ECG features with machine-learning methods to classify chronic pancreatitis patients with and without pain. Unlike prior work focusing only on EEG or clinical data, it shows that adding autonomic (ECG) information and tonic pain stimulation improves classification. Using robust, non-disease-specific signal features, the study demonstrates a feasible, bedside-friendly approach to pain phenotyping in chronic pancreatitis and establishes a foundation for more disease-specific models.

## Data Availability

The raw data supporting the conclusions of this article will be made available by the authors, upon reasonable request.
