# Supplementary material for: Electrophysiological biomarkers for foundational signal markers in chronic pancreatitis using machine learning models
Source: Front Pain Res (Lausanne). 2026 Jul 22;7:1857591. doi: 10.3389/fpain.2026.1857591 (PMC13438447; doi:10.3389/fpain.2026.1857591)
Supplement: Supplementary file 1 [file Datasheet1.pdf]

Supplementary File 1. Complete List of the 24 Features Extracted from Each Epoch

Each feature was extracted identically from the time domain epoch and from the frequency domain (Welch power spectral density) representation, giving two feature sets per epoch.

#	Feature	Group	Brief definition
1	Mean	Statistical	Average amplitude of the epoch
2	Standard deviation	Statistical	Dispersion of amplitude around the mean
3	Peak to peak amplitude	Statistical	Difference between maximum and minimum amplitude
4	Variance	Statistical	Squared dispersion of amplitude
5	Minimum	Statistical	Lowest amplitude value
6	Maximum	Statistical	Highest amplitude value
7	Index of minimum	Statistical	Sample position of the minimum
8	Index of maximum	Statistical	Sample position of the maximum
9	Mean square	Statistical	Mean of squared amplitude (signal power)
10	Root mean square	Statistical	Square root of the mean squared amplitude
11	Curve length	Other	Sum of absolute differences between consecutive samples
12	Skewness	Statistical	Asymmetry of the amplitude distribution
13	Kurtosis	Statistical	Peakedness of the amplitude distribution
14	Zero crossing	Other	Position of sign change, rate of signal change
15	Approximate entropy	Entropy	Regularity and predictability of the signal (order 2)
16	Permutation entropy	Entropy	Complexity from ordinal pattern distribution (order 3, normalised)
17	SVD entropy	Entropy	Complexity from the singular spectrum of the embedded signal (order 3)
18	Spectral entropy	Entropy	Irregularity of the power spectrum (Welch, normalised)
19	Sample entropy	Entropy	Regularity, less biased variant of approximate entropy (order 2)
20	Katz fractal dimension	Fractal	Waveform complexity from curve length and diameter
21	Higuchi fractal dimension	Fractal	Complexity of the signal treated as a fractal curve
22	Petrosian fractal	Fractal	Fractal complexity from

	dimension		sign changes in the derivative
23	Hjorth mobility	Statistical	Mean frequency, ratio of SD of first derivative to SD of the signal
24	Hjorth complexity	Statistical	Change in frequency, deviation of the signal from a pure sine
