# Supplementary material for: Electrophysiological biomarkers for foundational signal markers in chronic pancreatitis using machine learning models
Source: Front Pain Res (Lausanne). 2026 Jul 22;7:1857591. doi: 10.3389/fpain.2026.1857591 (PMC13438447; doi:10.3389/fpain.2026.1857591)
Supplement: Supplementary file 2 [file Datasheet2.pdf]

Binary Classification Task

0: Healthy control inidividuals

1: Chronic pancreatitis patients

Channels

- Channels ['T3', 'Cz', 'T4','P3', 'Pz', 'P4', 'O1', 'O2']

Epoch_level metrics - Time Domain - Cold Pressor

Models	fold_info	acc	f1_score	precision	recall	roc_auc
**DT**	*mean*	0.5924	0.6853	0.5596	0.8976	0.6583
	min_max	[0.53 ; 0.7]	[0.63 ; 0.76]	[0.52 ; 0.62]	[0.69 ; 1.0]	[0.57 ; 0.75]
**RF**	*mean*	0.7154	0.7098	0.7271	0.6963	0.7523
	min_max	[0.66 ; 0.82]	[0.65 ; 0.82]	[0.66 ; 0.86]	[0.59 ; 0.78]	[0.69 ; 0.84]
**LR**	*mean*	0.6762	0.6859	0.676	0.7109	0.709
	min_max	[0.61 ; 0.76]	[0.59 ; 0.75]	[0.59 ; 0.83]	[0.56 ; 0.83]	[0.63 ; 0.82]
**SVM**	*mean*	0.6643	0.7076	0.6375	0.8065	0.703
	min_max	[0.46 ; 0.83]	[0.56 ; 0.85]	[0.47 ; 0.79]	[0.67 ; 0.98]	[0.58 ; 0.84]

Epoch_level metrics - Frequency Domain - Cold Pressor

Models	fold_info	acc	f1_score	precision	recall	roc_auc
**DT**	*mean*	0.671	0.6917	0.6798	0.738	0.6358
	min_max	[0.5 ; 0.74]	[0.58 ; 0.78]	[0.5 ; 0.88]	[0.56 ; 0.95]	[0.48 ; 0.81]
**RF**	*mean*	0.695	0.6901	0.6967	0.6932	0.7174
	min_max	[0.65 ; 0.76]	[0.6 ; 0.79]	[0.66 ; 0.75]	[0.54 ; 0.89]	[0.62 ; 0.8]
**LR**	*mean*	0.6317	0.6071	0.646	0.5765	0.6257
	min_max	[0.55 ; 0.83]	[0.51 ; 0.83]	[0.56 ; 0.85]	[0.45 ; 0.81]	[0.51 ; 0.82]
**SVM**	*mean*	0.5241	0.6753	0.5146	0.9858	0.5761
	min_max	[0.5 ; 0.62]	[0.66 ; 0.71]	[0.5 ; 0.57]	[0.94 ; 1.0]	[0.38 ; 0.72]

Epoch_level metrics - Time Domain - Resting State

Models	fold_info	acc	f1_score	precision	recall	roc_auc
**DT**	*mean*	0.6808	0.6811	0.6955	0.6857	0.7029
	min_max	[0.6 ; 0.83]	[0.54 ; 0.82]	[0.58 ; 0.86]	[0.47 ; 0.84]	[0.57 ; 0.83]
**RF**	*mean*	0.717	0.719	0.7206	0.722	0.7772
	min_max	[0.65 ; 0.8]	[0.64 ; 0.79]	[0.64 ; 0.82]	[0.62 ; 0.79]	[0.71 ; 0.87]
**LR**	*mean*	0.7321	0.735	0.7375	0.7465	0.8006
	min_max	[0.63 ; 0.85]	[0.6 ; 0.84]	[0.64 ; 0.87]	[0.57 ; 0.88]	[0.65 ; 0.91]
**SVM**	*mean*	0.7393	0.7405	0.7379	0.7462	0.7681
	min_max	[0.57 ; 0.82]	[0.57 ; 0.82]	[0.57 ; 0.83]	[0.57 ; 0.87]	[0.55 ; 0.89]

Epoch_level metrics - Frequency Domain - Resting State

Models	fold_info	acc	f1_score	precision	recall	roc_auc
**DT**	*mean*	0.5955	0.6931	0.5621	0.9113	0.6365
	min_max	[0.54 ; 0.66]	[0.66 ; 0.71]	[0.52 ; 0.62]	[0.84 ; 1.0]	[0.6 ; 0.72]
**RF**	*mean*	0.66	0.6434	0.6732	0.6194	0.6995
	min_max	[0.58 ; 0.72]	[0.52 ; 0.71]	[0.61 ; 0.75]	[0.46 ; 0.68]	[0.57 ; 0.8]
**LR**	*mean*	0.5807	0.5558	0.5893	0.5318	0.6149
	min_max	[0.49 ; 0.73]	[0.39 ; 0.7]	[0.48 ; 0.78]	[0.33 ; 0.64]	[0.46 ; 0.81]
**SVM**	*mean*	0.5841	0.6557	0.5702	0.7852	0.6022
	min_max	[0.48 ; 0.73]	[0.59 ; 0.74]	[0.49 ; 0.71]	[0.65 ; 0.88]	[0.52 ; 0.77]

Binary Classification Task

0: Healthy control inidividuals

1: Chronic pancreatitis patients

Channels

- EEG Channels ['ECG', 'T3', 'Cz', 'T4','P3', 'Pz', 'P4', 'O1', 'O2']

Epoch_level metrics - Time Domain - Cold Pressor

Models	fold_info	acc	f1_score	precision	recall	roc_auc
DT	*mean*	0.6362	0.7126	0.5973	0.8865	0.6467
	min_max	[0.41 ; 0.77]	[0.56 ; 0.81]	[0.44 ; 0.7]	[0.74 ; 0.97]	[0.48 ; 0.76]
RF	*mean*	0.7747	0.7653	0.7977	0.7437	0.8252
	min_max	[0.7 ; 0.9]	[0.69 ; 0.9]	[0.71 ; 0.9]	[0.67 ; 0.94]	[0.73 ; 0.96]
LR	*mean*	0.7182	0.7205	0.7147	0.7334	0.7937
	min_max	[0.66 ; 0.76]	[0.65 ; 0.78]	[0.67 ; 0.77]	[0.64 ; 0.85]	[0.67 ; 0.9]
SVM	*mean*	0.6528	0.7195	0.6061	0.889	0.6967
	min_max	[0.56 ; 0.78]	[0.67 ; 0.81]	[0.53 ; 0.7]	[0.78 ; 0.99]	[0.54 ; 0.83]

Epoch_level metrics - Frequency Domain - Cold Pressor

Models	fold_info	acc	f1_score	precision	recall	roc_auc
DT	*mean*	0.74	0.7186	0.8091	0.6598	0.6908
	min_max	[0.69 ; 0.8]	[0.67 ; 0.76]	[0.67 ; 0.96]	[0.61 ; 0.78]	[0.62 ; 0.79]
RF	*mean*	0.7438	0.747	0.747	0.7526	0.8322
	min_max	[0.68 ; 0.86]	[0.7 ; 0.86]	[0.64 ; 0.88]	[0.67 ; 0.83]	[0.78 ; 0.93]
LR	*mean*	0.6993	0.6887	0.7113	0.6696	0.7199
	min_max	[0.61 ; 0.88]	[0.61 ; 0.88]	[0.62 ; 0.86]	[0.61 ; 0.89]	[0.63 ; 0.88]
SVM	*mean*	0.7563	0.7274	0.8031	0.6683	0.8052
	min_max	[0.62 ; 0.9]	[0.55 ; 0.9]	[0.67 ; 0.91]	[0.47 ; 0.89]	[0.63 ; 0.95]

Epoch_level metrics - Time Domain - Resting State

Models	fold_info	acc	f1_score	precision	recall	roc_auc
DT	*mean*	0.6828	0.7208	0.6531	0.813	0.6992
	min_max	[0.61 ; 0.84]	[0.63 ; 0.85]	[0.58 ; 0.83]	[0.68 ; 0.94]	[0.6 ; 0.89]
RF	*mean*	0.7714	0.7669	0.7891	0.7541	0.8596
	min_max	[0.68 ; 0.83]	[0.69 ; 0.83]	[0.67 ; 0.89]	[0.63 ; 0.84]	[0.78 ; 0.91]
LR	*mean*	0.772	0.7615	0.8097	0.7237	0.8274
	min_max	[0.68 ; 0.86]	[0.69 ; 0.85]	[0.66 ; 0.91]	[0.65 ; 0.79]	[0.75 ; 0.91]
SVM	*mean*	0.811	0.7926	0.8788	0.723	0.8679
	min_max	[0.74 ; 0.89]	[0.72 ; 0.88]	[0.78 ; 1.0]	[0.64 ; 0.8]	[0.77 ; 0.96]

Epoch_level metrics - Frequency Domain - Resting State

Models	fold_info	acc	f1_score	precision	recall	roc_auc
DT	*mean*	0.7137	0.6973	0.7421	0.6588	0.7025
	min_max	[0.65 ; 0.83]	[0.65 ; 0.82]	[0.65 ; 0.87]	[0.6 ; 0.78]	[0.64 ; 0.83]
RF	*mean*	0.7734	0.7667	0.7774	0.7642	0.8393
	min_max	[0.64 ; 0.84]	[0.62 ; 0.86]	[0.65 ; 0.84]	[0.59 ; 0.96]	[0.65 ; 0.92]
LR	*mean*	0.6848	0.6617	0.6895	0.6442	0.7246
	min_max	[0.59 ; 0.87]	[0.55 ; 0.87]	[0.61 ; 0.84]	[0.5 ; 0.92]	[0.58 ; 0.95]
SVM	*mean*	0.71	0.6971	0.7189	0.6813	0.743
	min_max	[0.61 ; 0.84]	[0.56 ; 0.84]	[0.62 ; 0.83]	[0.5 ; 0.84]	[0.62 ; 0.91]

Binary Classification Task

0: Painfree Chronic Pancreatitis

1: Painful pancreatitis patients

Channels

- EEG Channels ['T3', 'Cz', 'T4','P3', 'Pz', 'P4', 'O1', 'O2']

Epoch_level metrics - Time Domain - Cold Pressor

Models	fold_info	acc	f1_score	precision	recall	roc_auc
DT	*mean*	0.5699	0.6735	0.5429	0.8878	0.572
	min_max	[0.39 ; 0.65]	[0.53 ; 0.74]	[0.43 ; 0.6]	[0.69 ; 1.0]	[0.29 ; 0.67]
RF	*mean*	0.5557	0.5815	0.5548	0.6169	0.5546
	min_max	[0.31 ; 0.76]	[0.38 ; 0.77]	[0.34 ; 0.76]	[0.42 ; 0.81]	[0.29 ; 0.81]
LR	*mean*	0.4876	0.5234	0.4966	0.5606	0.4805
	min_max	[0.37 ; 0.58]	[0.43 ; 0.61]	[0.41 ; 0.59]	[0.44 ; 0.67]	[0.32 ; 0.64]
SVM	*mean*	0.5726	0.6773	0.5472	0.8963	0.5551
	min_max	[0.5 ; 0.68]	[0.64 ; 0.75]	[0.5 ; 0.62]	[0.78 ; 1.0]	[0.41 ; 0.71]

Epoch_level metrics - Frequency Domain - Cold Pressor

Models	fold_info	acc	f1_score	precision	recall	roc_auc
DT	*mean*	0.552	0.6451	0.535	0.8146	0.5464
	min_max	[0.44 ; 0.65]	[0.58 ; 0.72]	[0.47 ; 0.6]	[0.72 ; 0.92]	[0.46 ; 0.63]
RF	*mean*	0.5913	0.6235	0.5839	0.6749	0.6027
	min_max	[0.51 ; 0.65]	[0.57 ; 0.66]	[0.51 ; 0.67]	[0.61 ; 0.75]	[0.49 ; 0.69]
LR	*mean*	0.5439	0.5427	0.5456	0.5622	0.5692
	min_max	[0.5 ; 0.57]	[0.44 ; 0.63]	[0.5 ; 0.59]	[0.37 ; 0.78]	[0.47 ; 0.69]
SVM	*mean*	0.5713	0.683	0.5441	0.9241	0.5886
	min_max	[0.53 ; 0.61]	[0.67 ; 0.71]	[0.51 ; 0.57]	[0.83 ; 1.0]	[0.51 ; 0.69]

Epoch_level metrics - Time Domain - Resting State

Models	fold_info	acc	f1_score	precision	recall	roc_auc
DT	*mean*	0.5494	0.6078	0.5539	0.7158	0.5112
	min_max	[0.44 ; 0.65]	[0.53 ; 0.7]	[0.47 ; 0.68]	[0.56 ; 1.0]	[0.3 ; 0.63]
RF	*mean*	0.5086	0.5342	0.4993	0.5819	0.4923
	min_max	[0.3 ; 0.67]	[0.31 ; 0.71]	[0.31 ; 0.63]	[0.31 ; 0.81]	[0.27 ; 0.62]
LR	*mean*	0.4987	0.515	0.507	0.534	0.4863
	min_max	[0.36 ; 0.66]	[0.41 ; 0.65]	[0.38 ; 0.67]	[0.44 ; 0.7]	[0.38 ; 0.67]
SVM	*mean*	0.5333	0.649	0.5206	0.8684	0.5344
	min_max	[0.48 ; 0.57]	[0.61 ; 0.7]	[0.49 ; 0.54]	[0.76 ; 1.0]	[0.47 ; 0.63]

Epoch_level metrics - Frequency Domain - Resting State

Models	fold_info	acc	f1_score	precision	recall	roc_auc
DT	*mean*	0.5482	0.6123	0.5363	0.7262	0.5179
	min_max	[0.49 ; 0.63]	[0.5 ; 0.68]	[0.49 ; 0.61]	[0.52 ; 0.91]	[0.45 ; 0.6]
RF	*mean*	0.478	0.5336	0.4856	0.6222	0.4291
	min_max	[0.3 ; 0.61]	[0.41 ; 0.7]	[0.36 ; 0.57]	[0.34 ; 0.89]	[0.24 ; 0.53]
LR	*mean*	0.4489	0.4763	0.4502	0.5187	0.4002
	min_max	[0.37 ; 0.57]	[0.31 ; 0.6]	[0.39 ; 0.56]	[0.26 ; 0.68]	[0.31 ; 0.59]
SVM	*mean*	0.4956	0.6449	0.498	0.9186	0.5126
	min_max	[0.4 ; 0.55]	[0.57 ; 0.69]	[0.44 ; 0.53]	[0.8 ; 1.0]	[0.4 ; 0.6]

Binary Classification Task

0: Painfree Chronic Pancreatitis

1: Painful pancreatitis patients

Channels

- Channels ['ECG', 'T3', 'Cz', 'T4','P3', 'Pz', 'P4', 'O1', 'O2']

Epoch_level metrics - Time Domain - Cold Pressor

Models	fold_info	acc	f1_score	precision	recall	roc_auc
DT	*mean*	0.6516	0.6901	0.6274	0.7688	0.6644
	min_max	[0.54 ; 0.75]	[0.61 ; 0.77]	[0.53 ; 0.71]	[0.69 ; 0.83]	[0.52 ; 0.76]
RF	*mean*	0.7194	0.7495	0.6943	0.8241	0.6889
	min_max	[0.5 ; 0.83]	[0.57 ; 0.83]	[0.5 ; 0.83]	[0.67 ; 0.97]	[0.47 ; 0.84]
LR	*mean*	0.6873	0.7231	0.675	0.8098	0.7065
	min_max	[0.55 ; 0.83]	[0.63 ; 0.84]	[0.53 ; 0.8]	[0.59 ; 1.0]	[0.48 ; 0.87]
SVM	*mean*	0.6468	0.7106	0.6065	0.8659	0.6976
	min_max	[0.58 ; 0.74]	[0.65 ; 0.77]	[0.56 ; 0.71]	[0.78 ; 1.0]	[0.52 ; 0.91]

Epoch_level metrics - Frequency Domain - Cold Pressor

Models	fold_info	acc	f1_score	precision	recall	roc_auc
DT	*mean*	0.6258	0.6394	0.6192	0.6709	0.6107
	min_max	[0.56 ; 0.67]	[0.57 ; 0.69]	[0.56 ; 0.67]	[0.56 ; 0.83]	[0.54 ; 0.64]
RF	*mean*	0.6037	0.6264	0.5955	0.6765	0.6306
	min_max	[0.5 ; 0.67]	[0.54 ; 0.7]	[0.5 ; 0.66]	[0.48 ; 0.81]	[0.55 ; 0.72]
LR	*mean*	0.5582	0.5382	0.5585	0.5466	0.599
	min_max	[0.51 ; 0.62]	[0.41 ; 0.68]	[0.52 ; 0.59]	[0.33 ; 0.81]	[0.5 ; 0.75]
SVM	*mean*	0.6163	0.7144	0.5744	0.9519	0.649
	min_max	[0.56 ; 0.71]	[0.68 ; 0.76]	[0.53 ; 0.65]	[0.92 ; 1.0]	[0.58 ; 0.72]

Epoch_level metrics - Time Domain - Resting State

Models	fold_info	acc	f1_score	precision	recall	roc_auc
DT	*mean*	0.6014	0.6252	0.5816	0.6932	0.5681
	min_max	[0.46 ; 0.82]	[0.45 ; 0.82]	[0.46 ; 0.81]	[0.44 ; 0.98]	[0.42 ; 0.81]
RF	*mean*	0.5701	0.5711	0.5648	0.5809	0.5478
	min_max	[0.47 ; 0.68]	[0.42 ; 0.68]	[0.46 ; 0.68]	[0.38 ; 0.68]	[0.44 ; 0.7]
LR	*mean*	0.5025	0.5173	0.505	0.5386	0.4336
	min_max	[0.41 ; 0.63]	[0.4 ; 0.62]	[0.41 ; 0.64]	[0.39 ; 0.68]	[0.39 ; 0.57]
SVM	*mean*	0.5222	0.6594	0.516	0.9184	0.523
	min_max	[0.42 ; 0.64]	[0.59 ; 0.72]	[0.45 ; 0.59]	[0.83 ; 0.99]	[0.44 ; 0.62]

Epoch_level metrics - Frequency Domain - Resting State

Models	fold_info	acc	f1_score	precision	recall	roc_auc
DT	*mean*	0.5524	0.6687	0.5334	0.9046	0.5316
	min_max	[0.47 ; 0.61]	[0.62 ; 0.72]	[0.48 ; 0.58]	[0.76 ; 0.99]	[0.46 ; 0.64]
RF	*mean*	0.4638	0.516	0.4733	0.5857	0.4242
	min_max	[0.3 ; 0.59]	[0.41 ; 0.65]	[0.35 ; 0.56]	[0.35 ; 0.77]	[0.16 ; 0.54]
LR	*mean*	0.4691	0.4731	0.4691	0.4842	0.4128
	min_max	[0.36 ; 0.63]	[0.31 ; 0.63]	[0.37 ; 0.64]	[0.27 ; 0.63]	[0.28 ; 0.67]
SVM	*mean*	0.5022	0.6501	0.5019	0.9266	0.4789
	min_max	[0.41 ; 0.55]	[0.58 ; 0.69]	[0.45 ; 0.53]	[0.82 ; 1.0]	[0.35 ; 0.58]
