# Supplementary material for: Electrophysiological biomarkers for foundational signal markers in chronic pancreatitis using machine learning models
Source: Front Pain Res (Lausanne). 2026 Jul 22;7:1857591. doi: 10.3389/fpain.2026.1857591 (PMC13438447; doi:10.3389/fpain.2026.1857591)
Supplement: Supplementary file 3 [file Datasheet3.pdf]

Supplementary File 3. Model evaluation: repeated K-fold cross-validation (per-configuration distributions, figures and tables)

Contents

Results Control vs Chronic Pancreatitis. 2

Evaluation of Selected Candidate Model Configurations – EEG and ECG 2

Time Domain – Cold Pressor – EEG and EEG – RF 2

Time Domain – Resting – EEG and ECG – SVM 3

Frequency Domain – Cold Pressor – EEG and ECG – RF 4

Frequency Domain – Resting – EEG and ECG – DT 5

Evaluation of Selected Candidate Model Configurations – EEG 6

Time Domain – Cold Pressor – EEG – RF 6

Time Domain – Resting – EEG – RF 7

Frequency Domain – Cold Pressor – EEG – RF 8

Frequency Domain – Resting – EEG – RF 9

Results Pain-free vs Painful Chronic Pancreatitis 10

Evaluation of Selected Candidate Model Configurations – EEG and ECG 10

Time Domain – Cold Pressor – EEG and ECG – SVM 10

Time Domain – Resting – EEG and ECG – DT 11

Frequency Domain – Cold Pressor – EEG and ECG – DT 12

Frequency Domain – Resting – EEG and ECG – DT 13

Evaluation of Selected Candidate Model Configurations – EEG 14

Time Domain – Cold Pressor – EEG - SVM 14

Time Domain – Resting – EEG – DT 15

Frequency Domain – Cold Pressor – EEG – SVM 16

Frequency Domain – Resting – EEG – DT 17

Results Control vs Chronic Pancreatitis.

Evaluation of Selected Candidate Model Configurations – EEG and ECG

Time Domain – Cold Pressor – EEG and EEG – RF

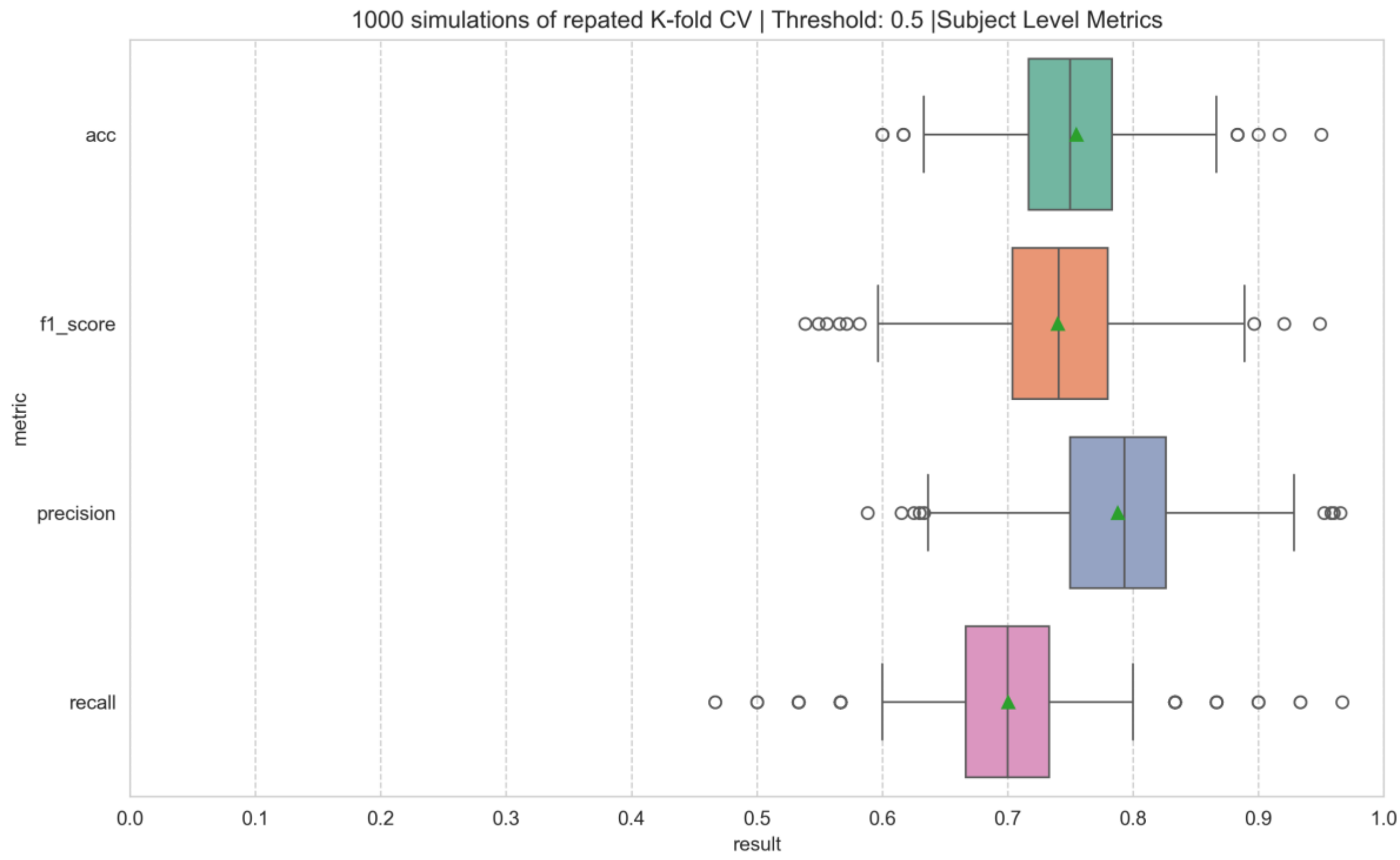

Fig x1: Caption

metric	mean	std	min	25%	median	75%	max	CI 2.5%	CI 97.5%
**acc**	0.7549	0.0513	0.6	0.7167	0.75	0.7833	0.95	0.65	0.85
**f1_score**	0.7398	0.0575	0.5385	0.7037	0.7407	0.7797	0.9492	0.6182	0.8519
**precision**	0.788	0.0601	0.5882	0.75	0.7931	0.8261	0.9655	0.6667	0.9091
**recall**	0.7004	0.0729	0.4667	0.6667	0.7	0.7333	0.9667	0.5667	0.8333

Table x1: Summary statistics of subject-level performance metrics across 1000 repeated K-fold cross-validation simulations for time-domain EEG-ECG features during the cold pressor condition using the selected Random Forest candidate configuration.

Time Domain – Resting – EEG and ECG – SVM

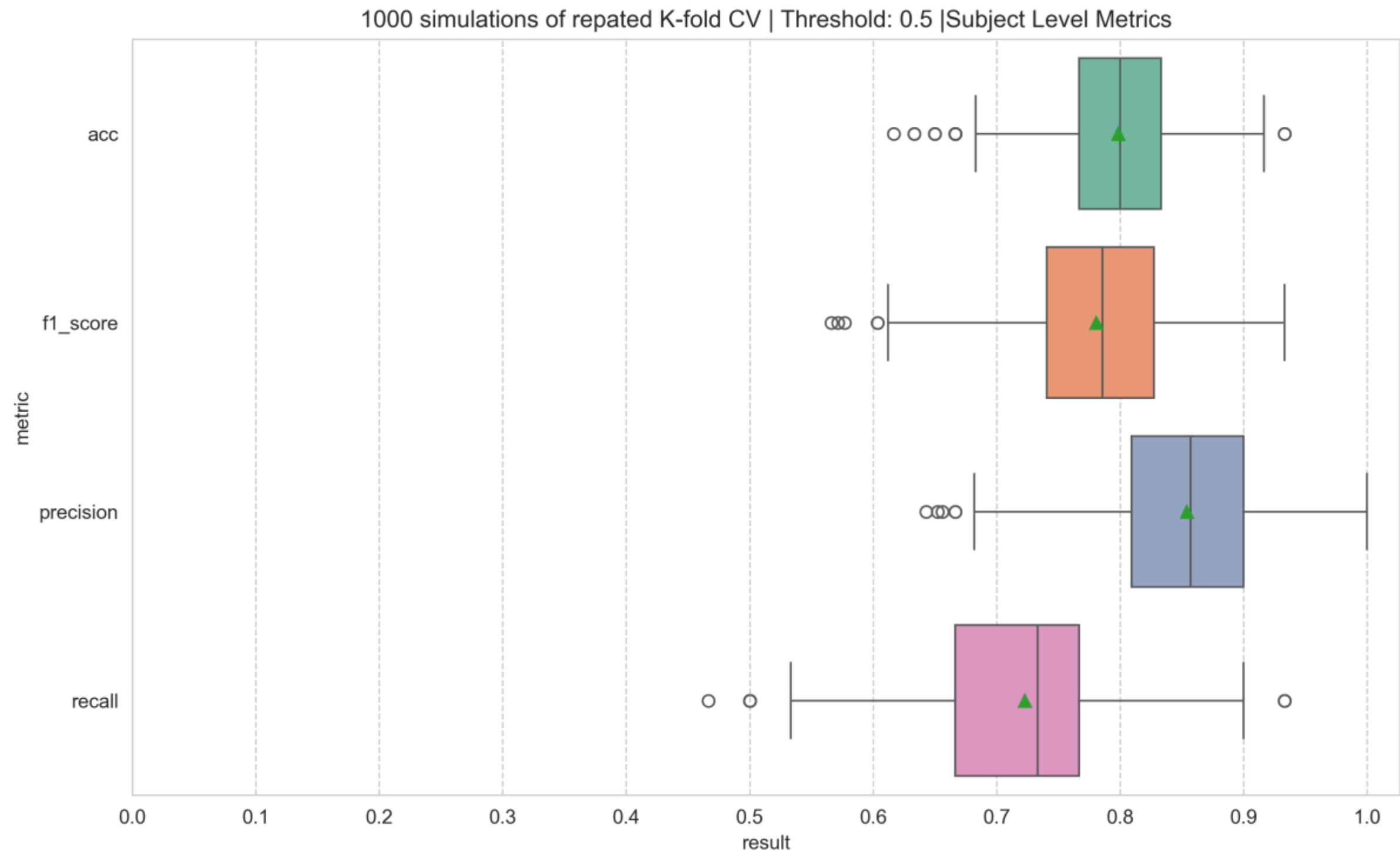

Fig x2: Caption

metric	mean	std	min	25%	median	75%	max	CI 2.5%	CI 97.5%
**acc**	0.7984	0.0533	0.6167	0.7667	0.8	0.8333	0.9333	0.7	0.9
**f1_score**	0.7807	0.0616	0.566	0.7407	0.7857	0.8276	0.9333	0.6538	0.8966
**precision**	0.854	0.0646	0.6429	0.8095	0.8571	0.9	1	0.7241	0.96
**recall**	0.7227	0.0777	0.4667	0.6667	0.7333	0.7667	0.9333	0.5667	0.8667

Table x2: Summary statistics of subject-level performance metrics across 1000 repeated K-fold cross-validation simulations for time-domain EEG-ECG features during the resting condition using the selected Support Vector Classifier candidate configuration.

Frequency Domain – Cold Pressor – EEG and ECG – RF

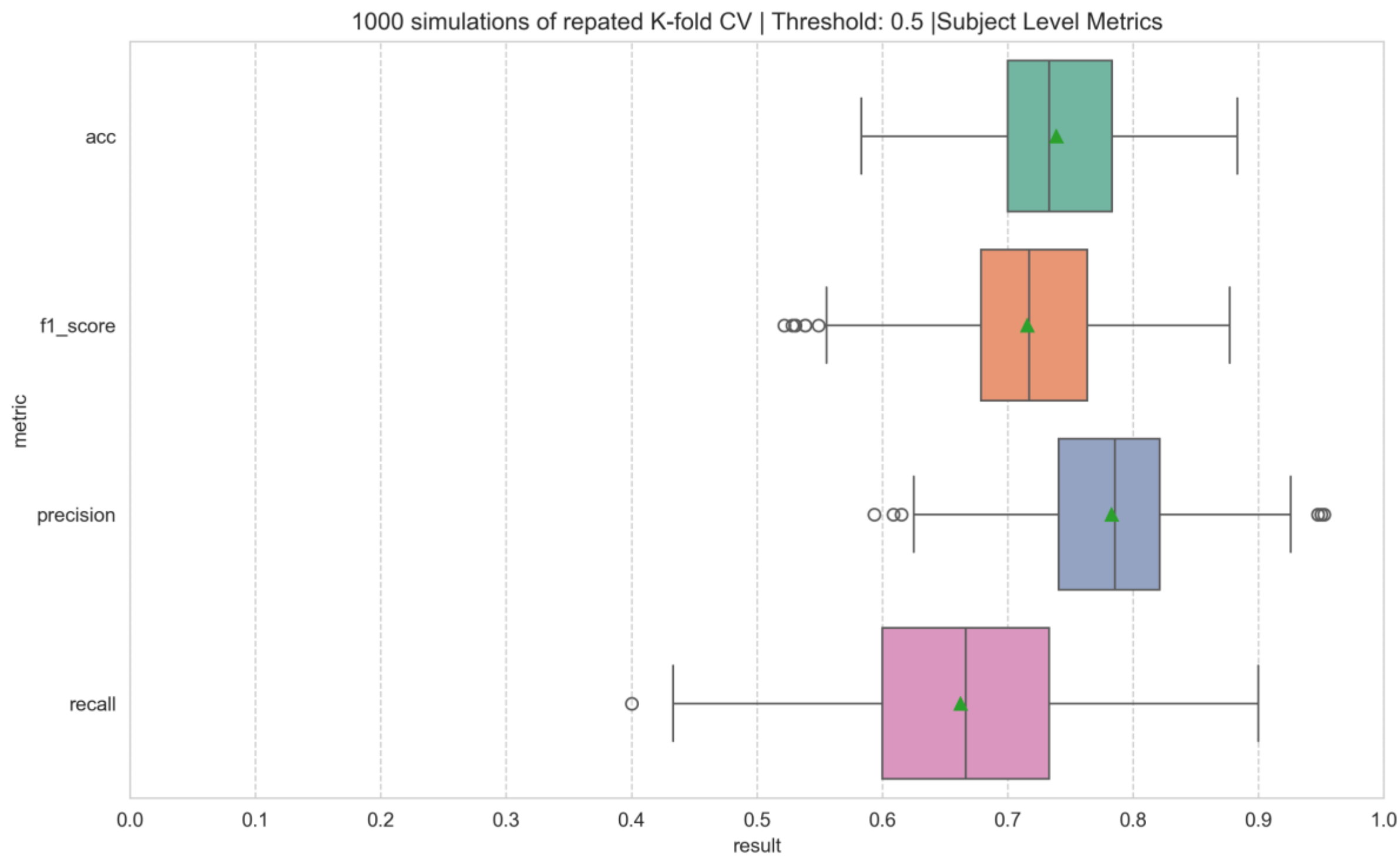

Fig x3: Capiton

metric	mean	std	min	25%	median	75%	max	CI 2.5%	CI 97.5%
**acc**	0.7388	0.0526	0.5833	0.7	0.7333	0.7833	0.8833	0.6333	0.8333
**f1_score**	0.7155	0.0635	0.5217	0.6786	0.717	0.7636	0.8772	0.5833	0.8276
**precision**	0.783	0.0603	0.5938	0.7407	0.7857	0.8214	0.9524	0.6552	0.9048
**recall**	0.6623	0.0806	0.4	0.6	0.6667	0.7333	0.9	0.5	0.8

Table x3: Summary statistics of subject-level performance metrics across 1000 repeated K-fold cross-validation simulations for frequency-domain EEG-ECG features during the cold pressor condition using the selected Random Forest candidate configuration.

Frequency Domain – Resting – EEG and ECG – RF

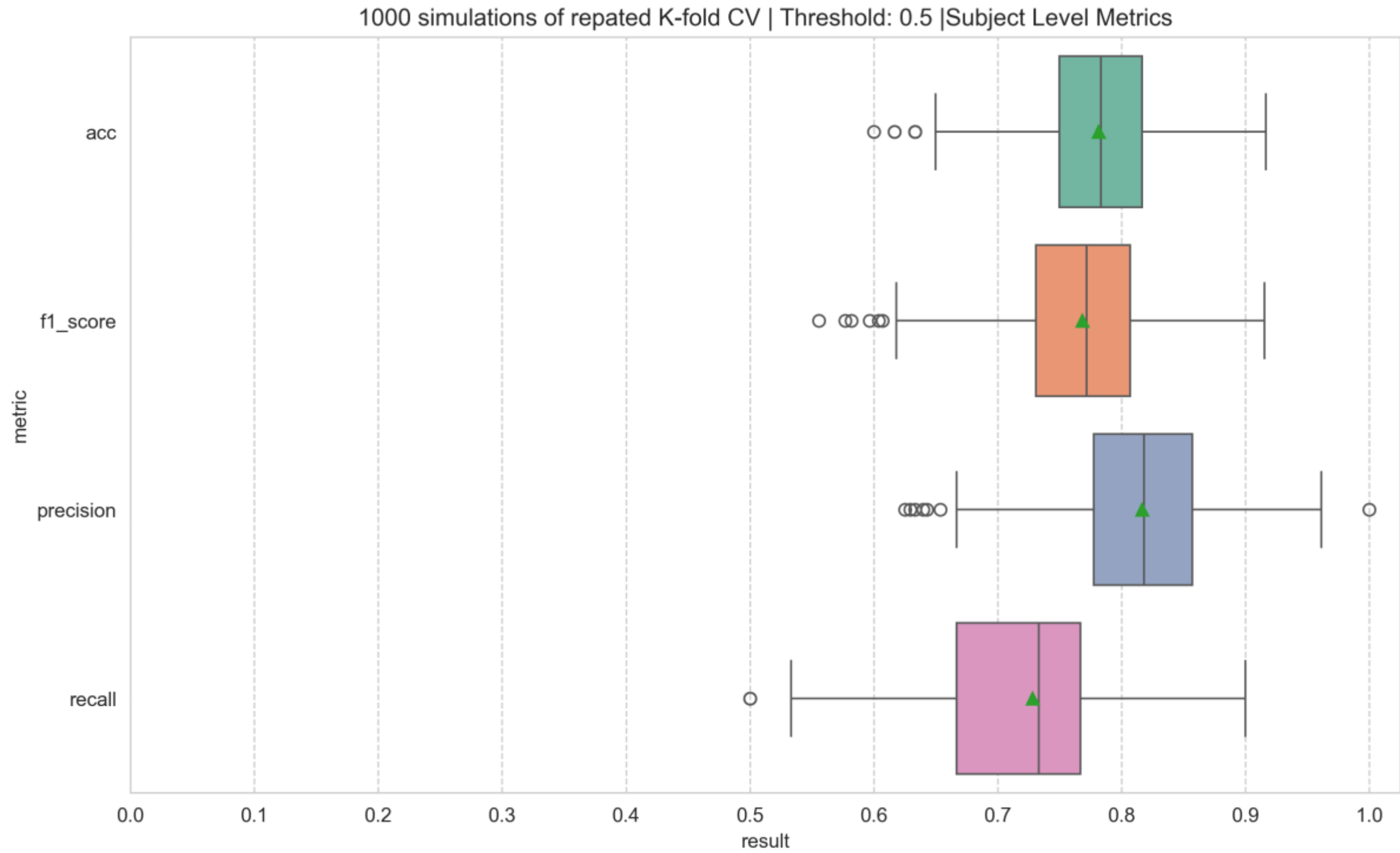

Fig x4: Caption

metric	mean	std	min	25%	median	75%	max	CI 2.5%	CI 97.5%
**acc**	0.7817	0.0505	0.6	0.75	0.7833	0.8167	0.9167	0.6833	0.8667
**f1_score**	0.7683	0.0573	0.5556	0.7308	0.7719	0.807	0.9153	0.6528	0.871
**precision**	0.8167	0.0569	0.625	0.7778	0.8182	0.8571	1	0.7037	0.92
**recall**	0.7283	0.0739	0.5	0.6667	0.7333	0.7667	0.9	0.5667	0.8667

Table x4: Summary statistics of subject-level performance metrics across 1000 repeated K-fold cross-validation simulations for frequency-domain EEG-ECG features during the resting condition using the selected Random Forest candidate configuration.

Evaluation of Selected Candidate Model Configurations – EEG

Time Domain – Cold Pressor – EEG – RF

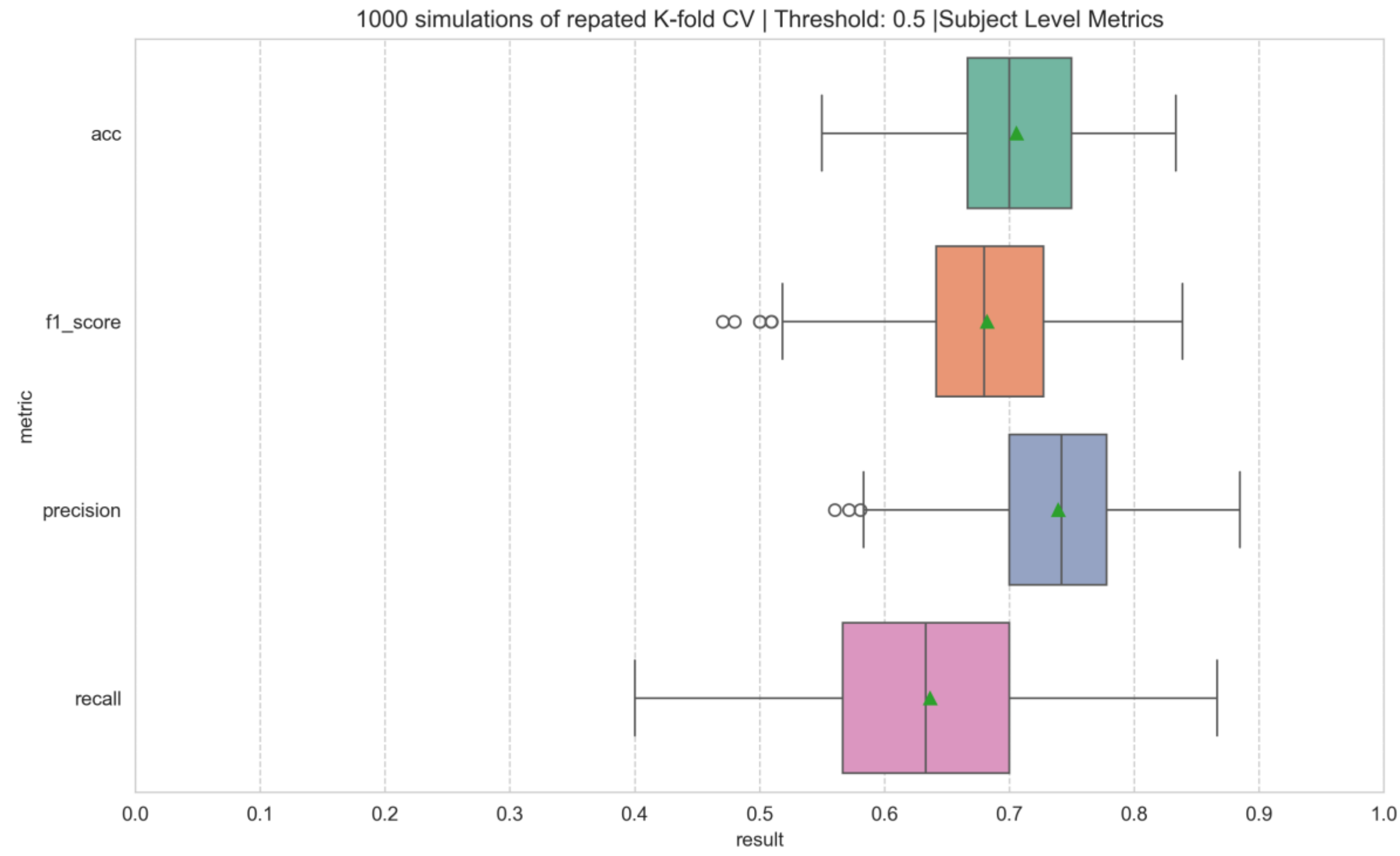

Fig x5:

metric	mean	std	min	25%	median	75%	max	CI 2.5%	CI 97.5%
**acc**	0.7057	0.0535	0.55	0.6667	0.7	0.75	0.8333	0.6	0.8
**f1_score**	0.682	0.0654	0.4706	0.6415	0.68	0.7273	0.8387	0.549	0.8
**precision**	0.7391	0.0581	0.56	0.7	0.7419	0.7778	0.8846	0.6189	0.8462
**recall**	0.6367	0.0834	0.4	0.5667	0.6333	0.7	0.8667	0.4667	0.8

Table x5: Summary statistics of subject-level performance metrics across 1000 repeated K-fold cross-validation simulations for time-domain EEG features during the cold pressor condition using the selected Random Forest candidate configuration.

Time Domain – Resting – EEG – SVM

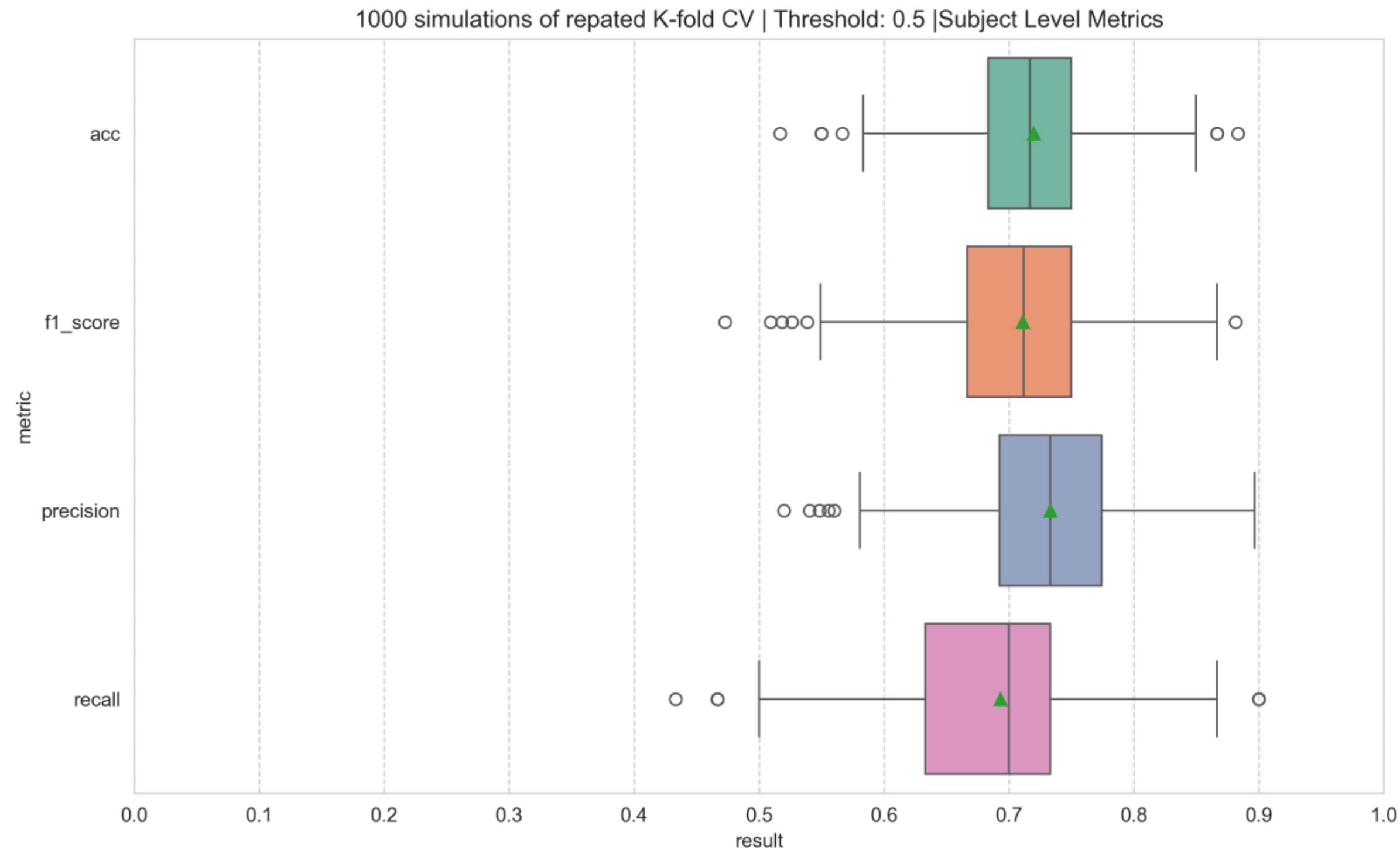

Fig x6:

metric	mean	std	min	25%	median	75%	max	CI 2.5%	CI 97.5%
**acc**	0.7197	0.0567	0.5167	0.6833	0.7167	0.75	0.8833	0.6	0.8333
**f1_score**	0.711	0.0621	0.4727	0.6667	0.7119	0.75	0.8814	0.5818	0.8276
**precision**	0.7333	0.0612	0.52	0.6923	0.7333	0.7742	0.8966	0.6154	0.8571
**recall**	0.6933	0.0784	0.4333	0.6333	0.7	0.7333	0.9	0.5333	0.8333

Table x6: Summary statistics of subject-level performance metrics across 1000 repeated K-fold cross-validation simulations for time-domain EEG features during the Resting condition using the selected Support Vector Classifier candidate configuration.

Frequency Domain – Cold Pressor – EEG – RF

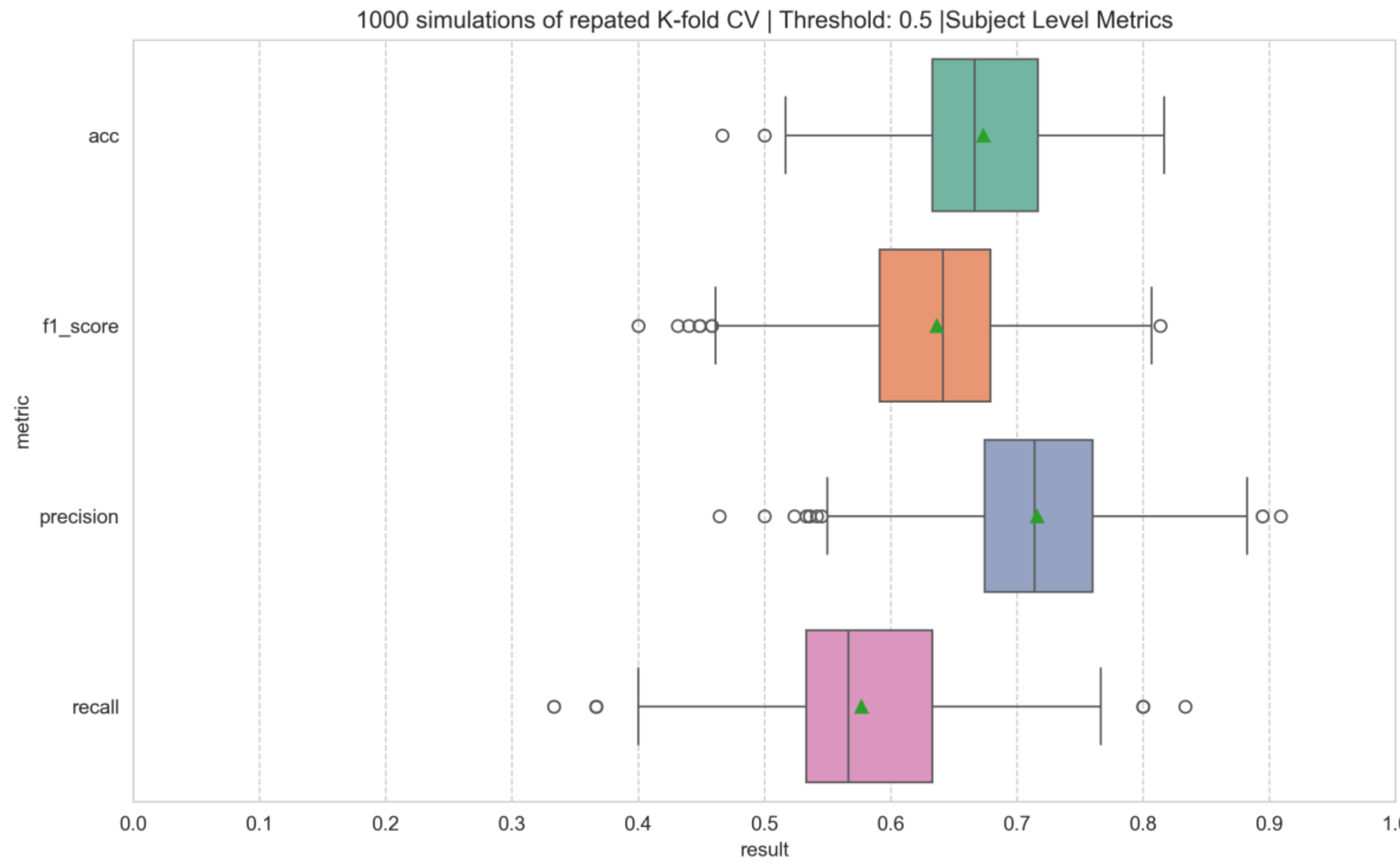

Fig x7:

metric	mean	std	min	25%	median	75%	max	CI 2.5%	CI 97.5%
**acc**	0.6734	0.0543	0.4667	0.6333	0.6667	0.7167	0.8167	0.5667	0.7833
**f1_score**	0.6365	0.0683	0.4	0.5915	0.6415	0.6792	0.8136	0.4906	0.7636
**precision**	0.716	0.0662	0.4643	0.6747	0.7143	0.76	0.9091	0.5789	0.8462
**recall**	0.5769	0.0824	0.3333	0.5333	0.5667	0.6333	0.8333	0.4333	0.7333

Table x7: Summary statistics of subject-level performance metrics across 1000 repeated K-fold cross-validation simulations for frequency domain EEG features during the cold pressor condition using the selected Random Forest candidate configuration.

Frequency Domain – Resting – EEG – RF

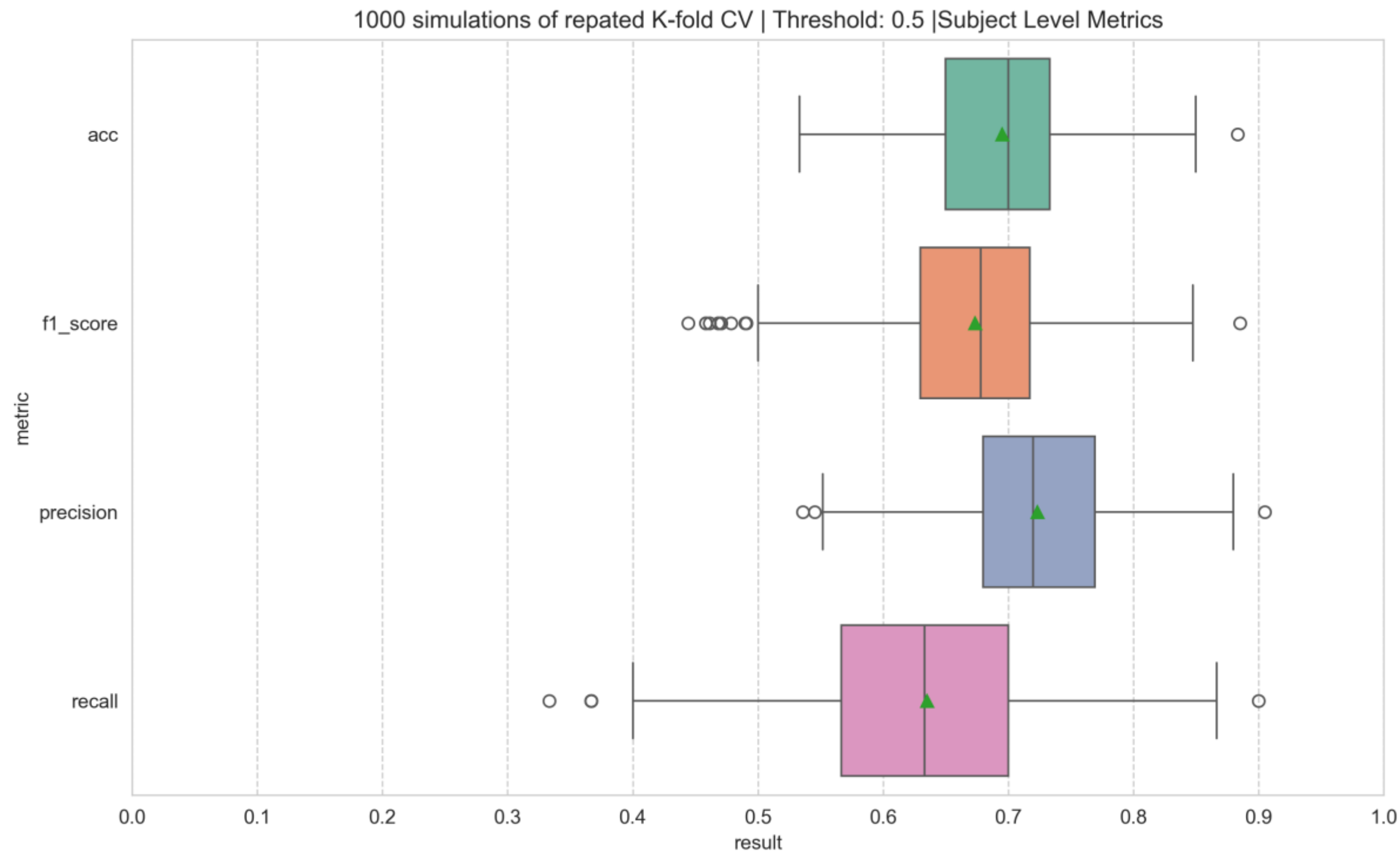

Fig x8:

metric	mean	std	min	25%	median	75%	max	CI 2.5%	CI 97.5%
**acc**	0.6952	0.0567	0.5333	0.65	0.7	0.7333	0.8833	0.5833	0.8
**f1_score**	0.6736	0.0688	0.4444	0.6296	0.678	0.717	0.8852	0.52	0.8
**precision**	0.7231	0.0637	0.5357	0.68	0.72	0.7692	0.9048	0.6	0.8462
**recall**	0.6349	0.0879	0.3333	0.5667	0.6333	0.7	0.9	0.4333	0.8

Table x7: Summary statistics of subject-level performance metrics across 1000 repeated K-fold cross-validation simulations for frequency domain EEG features during the resting condition using the selected Random Forest candidate configuration.

Results Pain-free vs Painful Chronic Pancreatitis

Evaluation of Selected Candidate Model Configurations – EEG and ECG

Time Domain – Cold Pressor – EEG and ECG – SVM

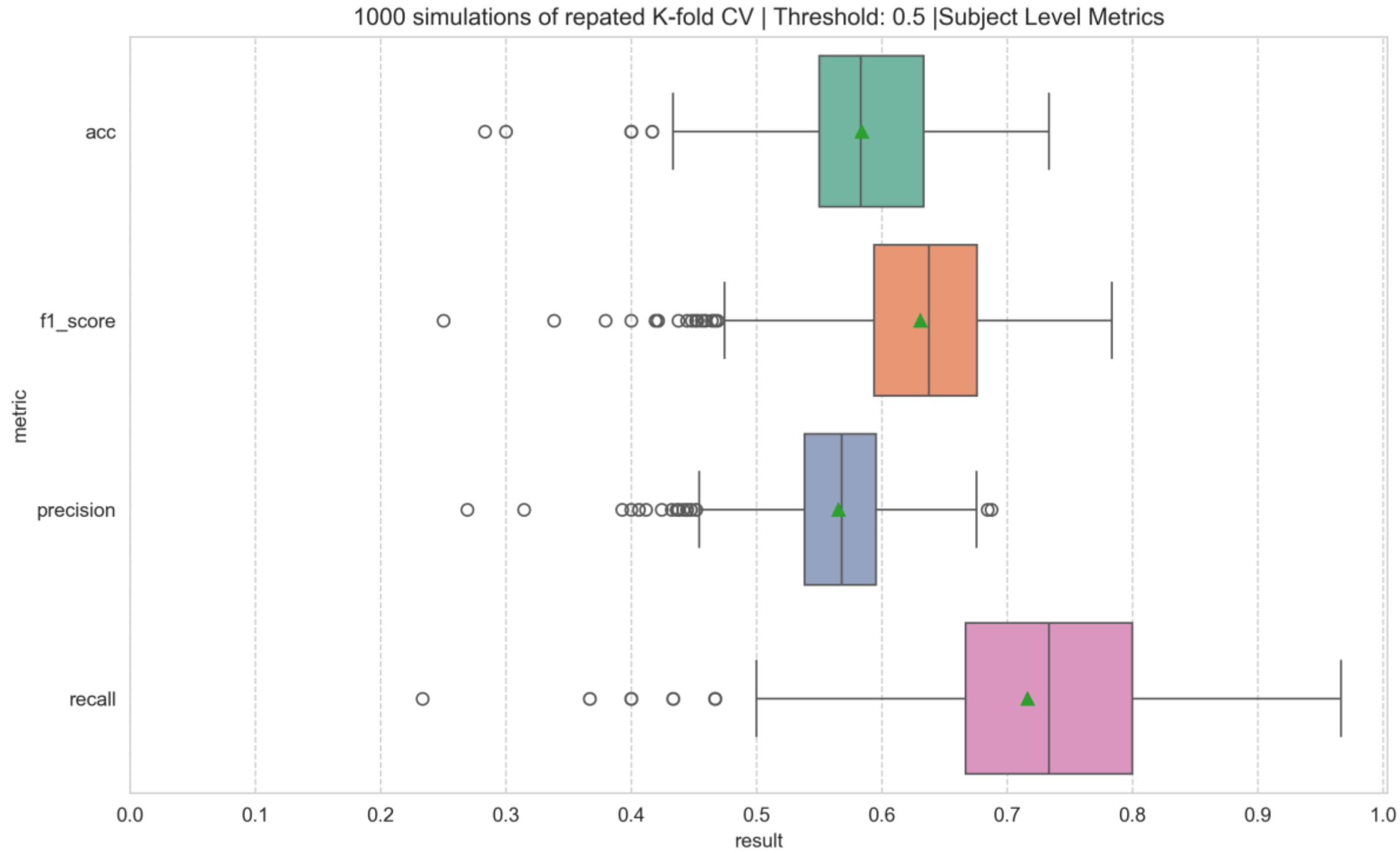

Fig x9:

metric	mean	std	min	25%	median	75%	max	CI 2.5%	CI 97.5%
**acc**	0.5841	0.061	0.2833	0.55	0.5833	0.6333	0.7333	0.45	0.6833
**f1_score**	0.6306	0.0654	0.25	0.5938	0.6377	0.6761	0.7838	0.4745	0.7324
**precision**	0.5652	0.0484	0.2692	0.5385	0.5682	0.5952	0.6875	0.457	0.6486
**recall**	0.7163	0.0977	0.2333	0.6667	0.7333	0.8	0.9667	0.4667	0.8667

Table x9: Summary statistics of subject-level performance metrics across 1000 repeated K-fold cross-validation simulations for time-domain EEG-ECG features during the cold pressor condition using the selected Support Vector Classifier candidate configuration.

Time Domain – Resting – EEG and ECG – DT

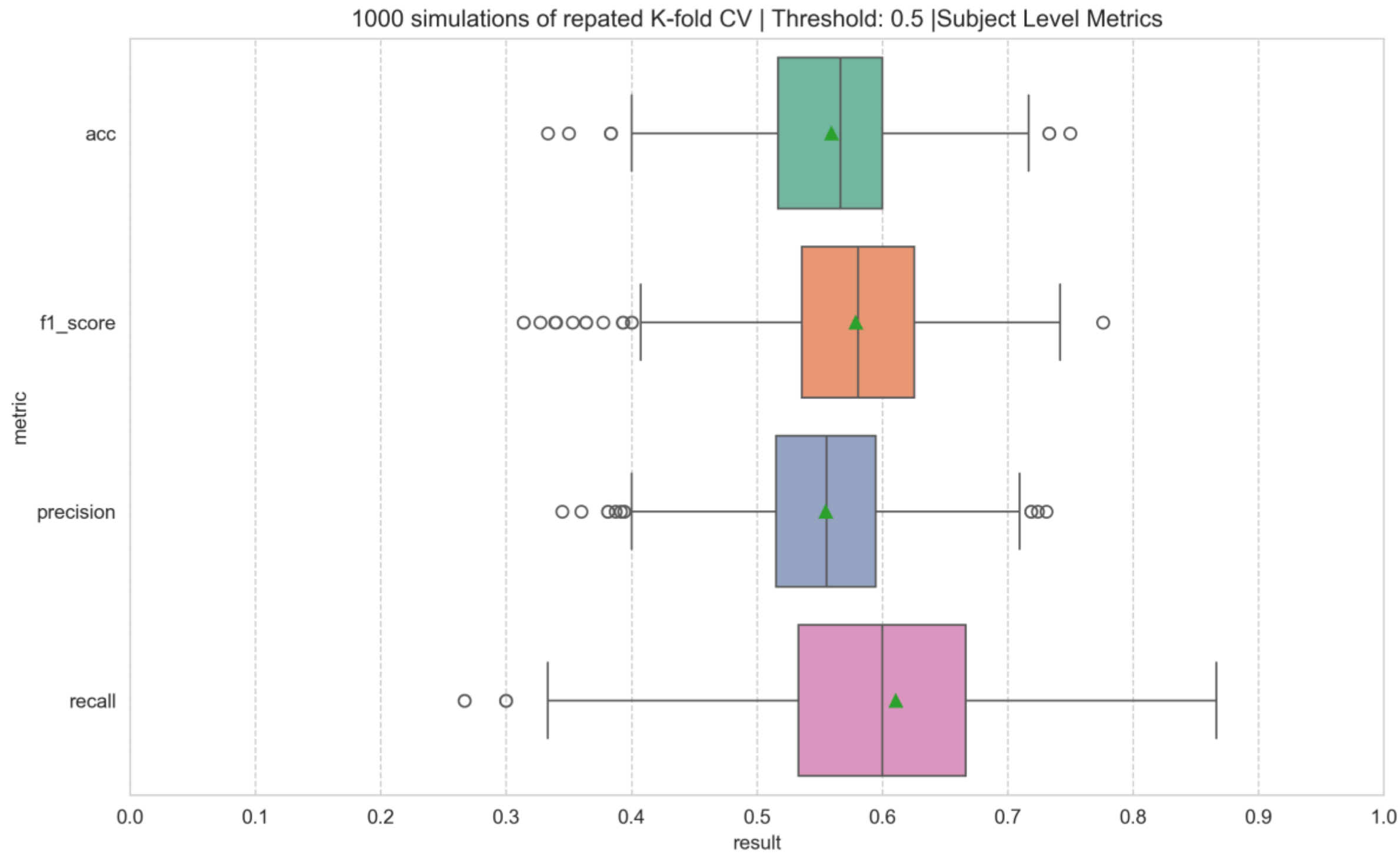

Fig x10:

metric	mean	std	min	25%	median	75%	max	CI 2.5%	CI 97.5%
**acc**	0.5595	0.0639	0.3333	0.5167	0.5667	0.6	0.75	0.4167	0.6833
**f1_score**	0.5787	0.0692	0.3137	0.5357	0.5806	0.6255	0.7761	0.4285	0.6984
**precision**	0.5548	0.0603	0.3448	0.5152	0.5556	0.5946	0.7308	0.4286	0.6667
**recall**	0.6106	0.0979	0.2667	0.5333	0.6	0.6667	0.8667	0.4	0.8

Table x10: Summary statistics of subject-level performance metrics across 1000 repeated K-fold cross-validation simulations for time-domain EEG-ECG features during the resting condition using the selected DT candidate configuration.

Frequency Domain – Cold Pressor – EEG and ECG – DT

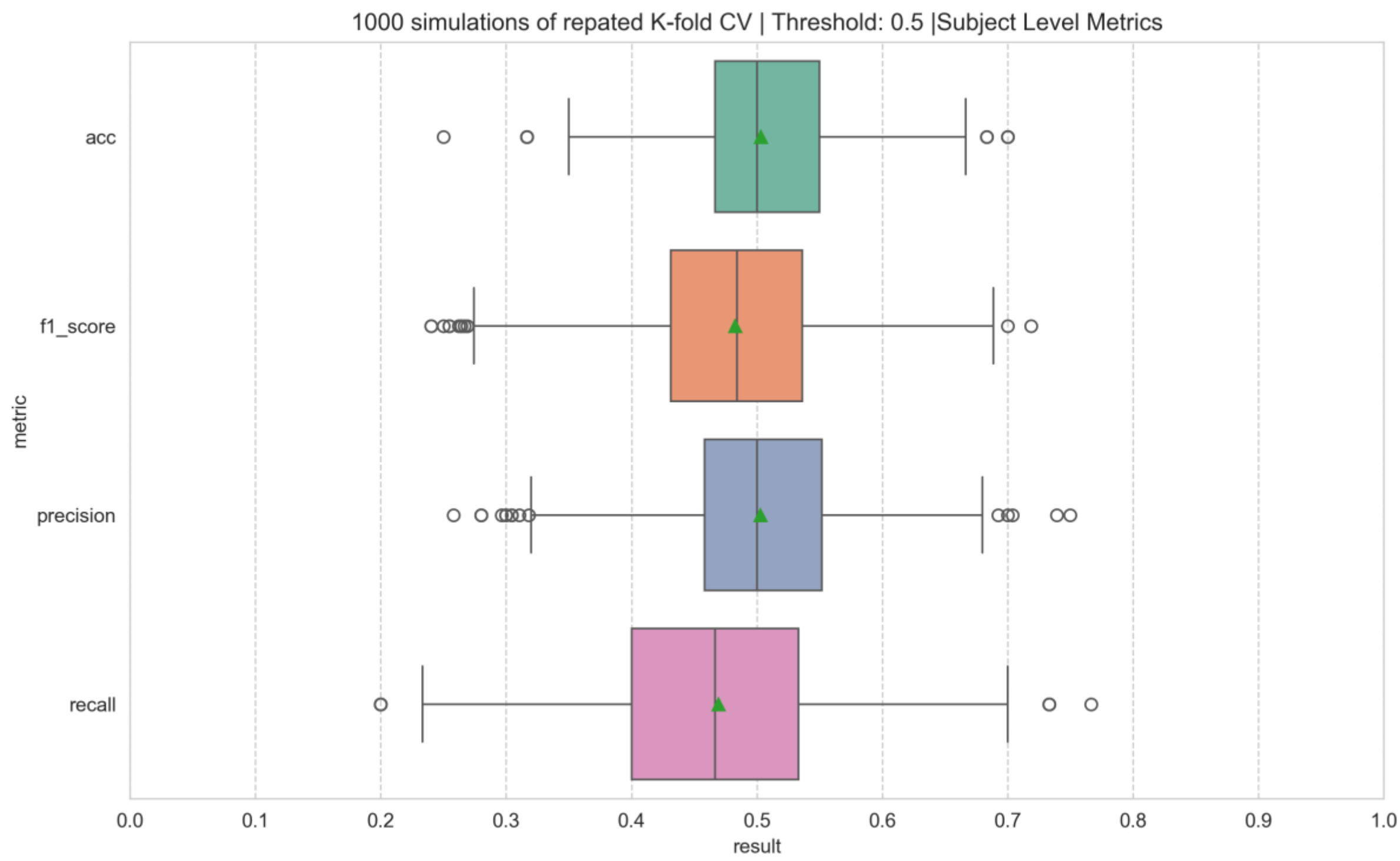

Fig x11:

metric	mean	std	min	25%	median	75%	max	CI 2.5%	CI 97.5%
**acc**	0.503	0.0659	0.25	0.4667	0.5	0.55	0.7	0.3667	0.6333
**f1_score**	0.4827	0.0798	0.24	0.4314	0.4844	0.5361	0.7188	0.3157	0.6349
**precision**	0.5028	0.0723	0.2581	0.4583	0.5	0.5517	0.75	0.3571	0.6429

**recall**	0.4694	0.0981	0.2	0.4	0.4667	0.5333	0.7667	0.2992	0.6667

Table x11: Summary statistics of subject-level performance metrics across 1000 repeated K-fold cross-validation simulations for frequency-domain EEG-ECG features during the cold pressor condition using the selected DT candidate configuration.

Frequency Domain – Resting – EEG and ECG – DT

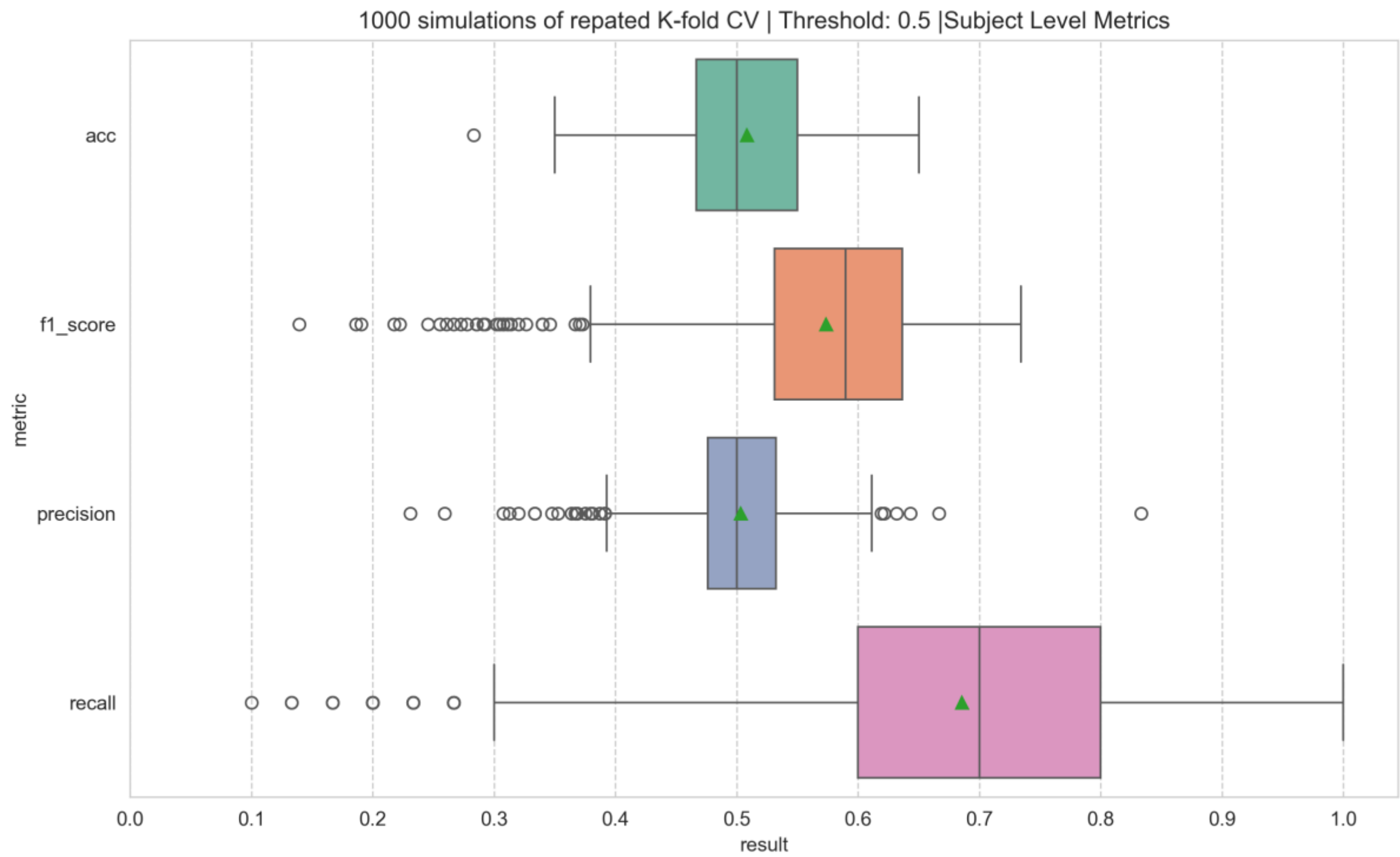

Fig: 12

metric	mean	std	min	25%	median	75%	max	CI 2.5%	CI 97.5%
**acc**	0.5083	0.0549	0.2833	0.4667	0.5	0.55	0.65	0.4	0.6167
**f1_score**	0.5735	0.0881	0.1395	0.5312	0.5897	0.6364	0.7342	0.3396	0.6988
**precision**	0.5032	0.0478	0.2308	0.4762	0.5	0.5322	0.8333	0.4053	0.5882

**recall**	0.6856	0.1649	0.1	0.6	0.7	0.8	1	0.3	0.9333

Table 12: Summary statistics of subject-level performance metrics across 1000 repeated K-fold cross-validation simulations for frequency-domain EEG-ECG features during the resting condition using the selected DT candidate configuration.

Evaluation of Selected Candidate Model Configurations – EEG

Time Domain – Cold Pressor – EEG - SVM

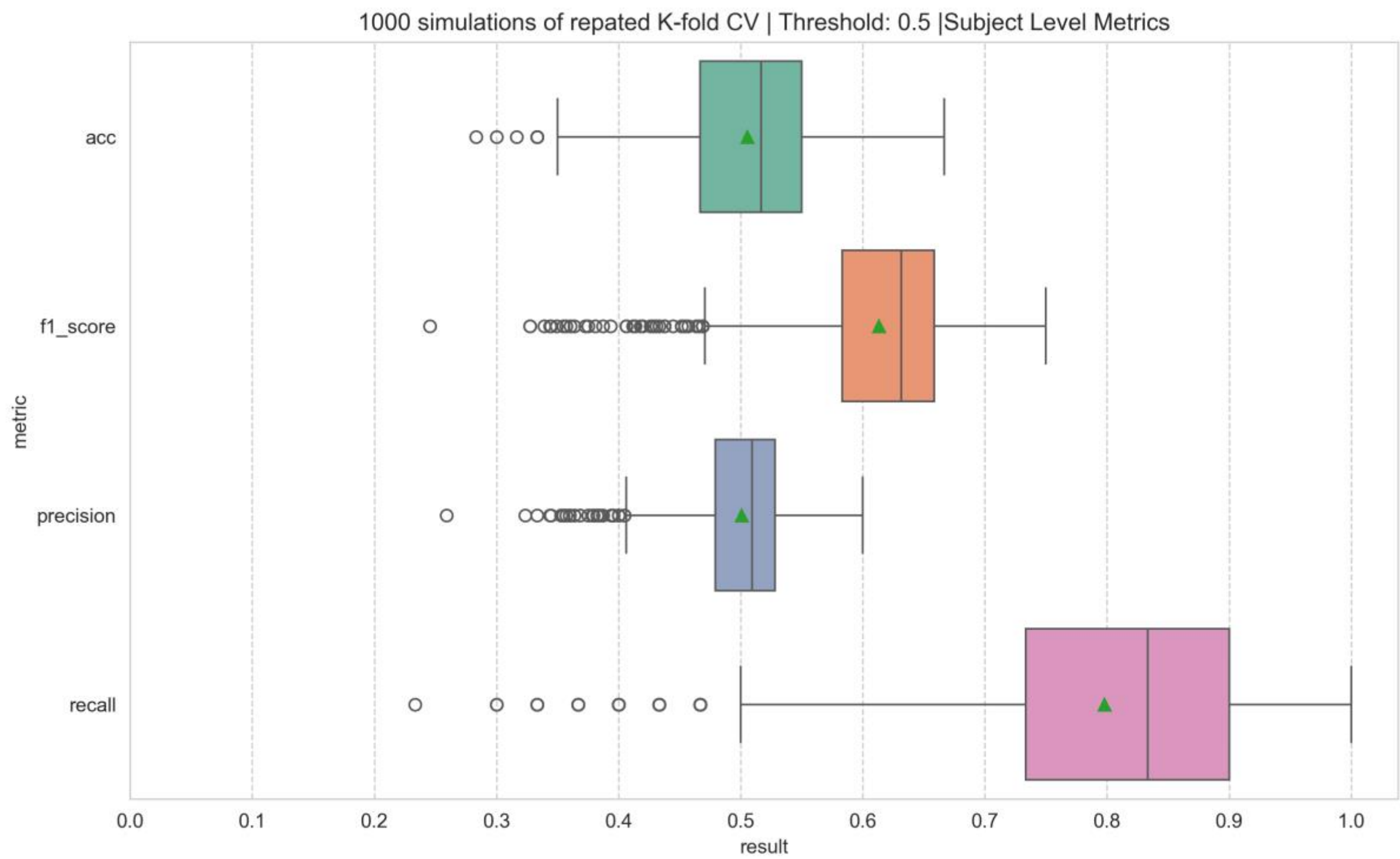

Fig 13:

metric	mean	std	min	25%	median	75%	max	CI 2.5%	CI 97.5%
**acc**	0.5053	0.0573	0.2833	0.4667	0.5167	0.55	0.6667	0.3833	0.6
**f1_score**	0.6133	0.0692	0.2456	0.5831	0.6316	0.6585	0.75	0.4262	0.7014
**precision**	0.5007	0.0405	0.2593	0.4792	0.5094	0.5283	0.6	0.4047	0.5625
**recall**	0.798	0.1305	0.2333	0.7333	0.8333	0.9	1	0.4333	0.9667

Table 13: Summary statistics of subject-level performance metrics across 1000 repeated K-fold cross-validation simulations for time-domain EE features during the cold pressor condition using the selected Support Vector Classifier candidate configuration.

Time Domain – Resting – EEG – DT

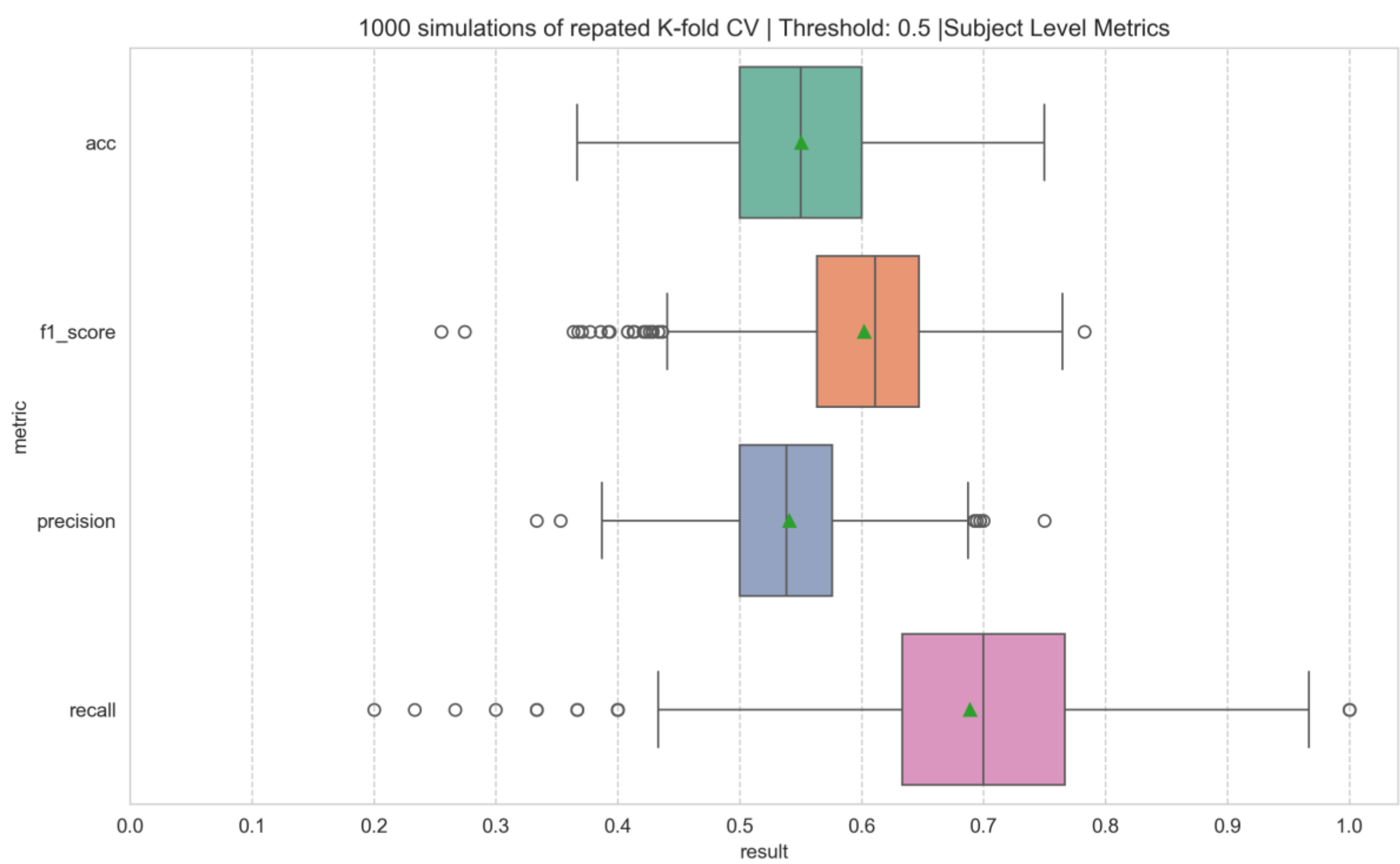

Fig 14:

metric	mean	std	min	25%	median	75%	max	CI 2.5%	CI 97.5%
acc	0.5503	0.0659	0.3667	0.5	0.55	0.6	0.75	0.4333	0.6833
f1_score	0.602	0.0699	0.2553	0.5634	0.6111	0.6471	0.7826	0.4333	0.7165
precision	0.5406	0.0554	0.3333	0.5	0.5385	0.5758	0.75	0.4333	0.6538
recall	0.6887	0.1165	0.2	0.6333	0.7	0.7667	1	0.4	0.8667

Table 14: Summary statistics of subject-level performance metrics across 1000 repeated K-fold cross-validation simulations for time-domain EE features during the resting condition using the selected Decision tree candidate configuration.

Frequency Domain – Cold Pressor – EEG – SVM

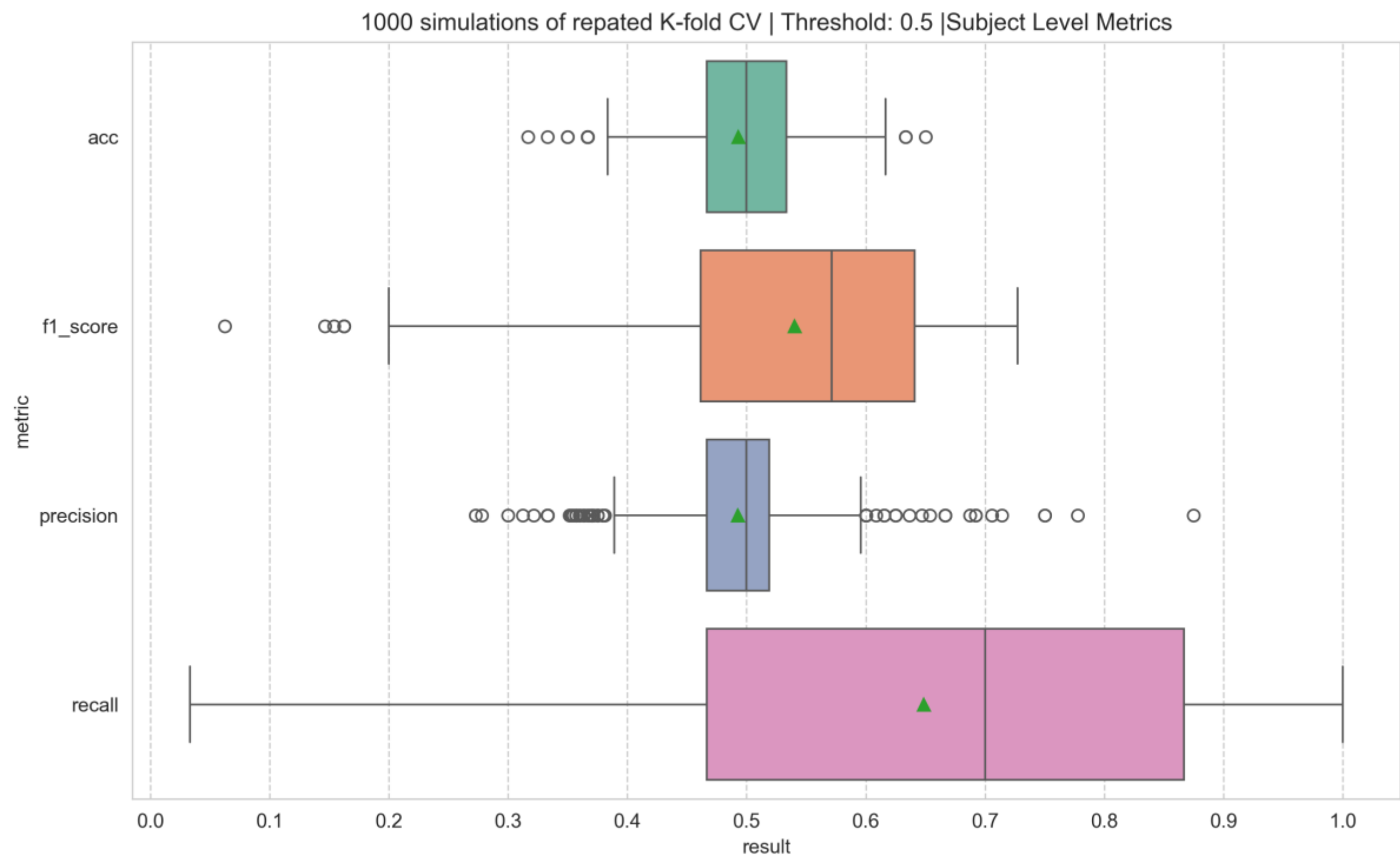

Fig 15:

metric	mean	std	min	25%	median	75%	max	CI 2.5%	CI 97.5%
acc	0.4931	0.0491	0.3167	0.4667	0.5	0.5333	0.65	0.3996	0.5833
f1_score	0.5403	0.1185	0.0625	0.4615	0.5714	0.641	0.7273	0.2727	0.6905
precision	0.4925	0.0537	0.2727	0.4667	0.5	0.5192	0.875	0.3749	0.6
recall	0.6485	0.236	0.0333	0.4667	0.7	0.8667	1	0.2	0.9667

Table 15: Summary statistics of subject-level performance metrics across 1000 repeated K-fold cross-validation simulations for frequency-domain EE features during the cold pressor condition using the selected Support Vector Classifier candidate configuration.

Frequency Domain – Resting – EEG – DT

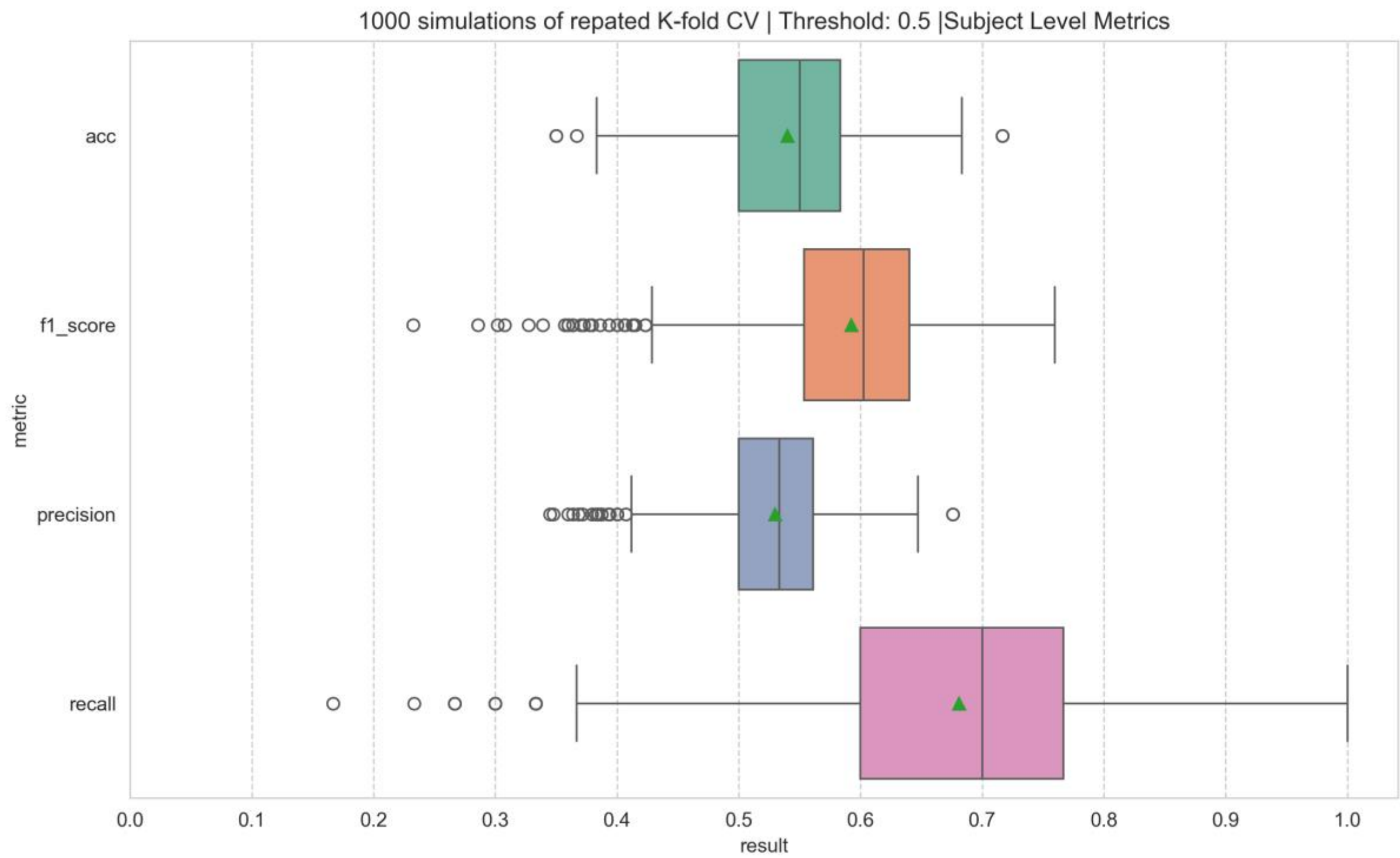

Fig 16:

metric	mean	std	min	25%	median	75%	max	CI 2.5%	CI 97.5%
acc	0.5401	0.0581	0.35	0.5	0.55	0.5833	0.7167	0.4167	0.65

**f1_score**	0.5927	0.0725	0.2326	0.5535	0.6027	0.6402	0.7595	0.4151	0.7042
**precision**	0.5298	0.0472	0.3448	0.5	0.5333	0.561	0.6757	0.4286	0.6136
**recall**	0.681	0.1224	0.1667	0.6	0.7	0.7667	1	0.4	0.8667

Table 16: Summary statistics of subject-level performance metrics across 1000 repeated K-fold cross-validation simulations for frequency-domain EE features during the resting condition using the selected DT candidate configuration.
