# Supplementary material for: Electrophysiological biomarkers for foundational signal markers in chronic pancreatitis using machine learning models
Source: Front Pain Res (Lausanne). 2026 Jul 22;7:1857591. doi: 10.3389/fpain.2026.1857591 (PMC13438447; doi:10.3389/fpain.2026.1857591)

CP vs Control – EEG

Time Domain – Cold Pressor – EEG | CP vs Control

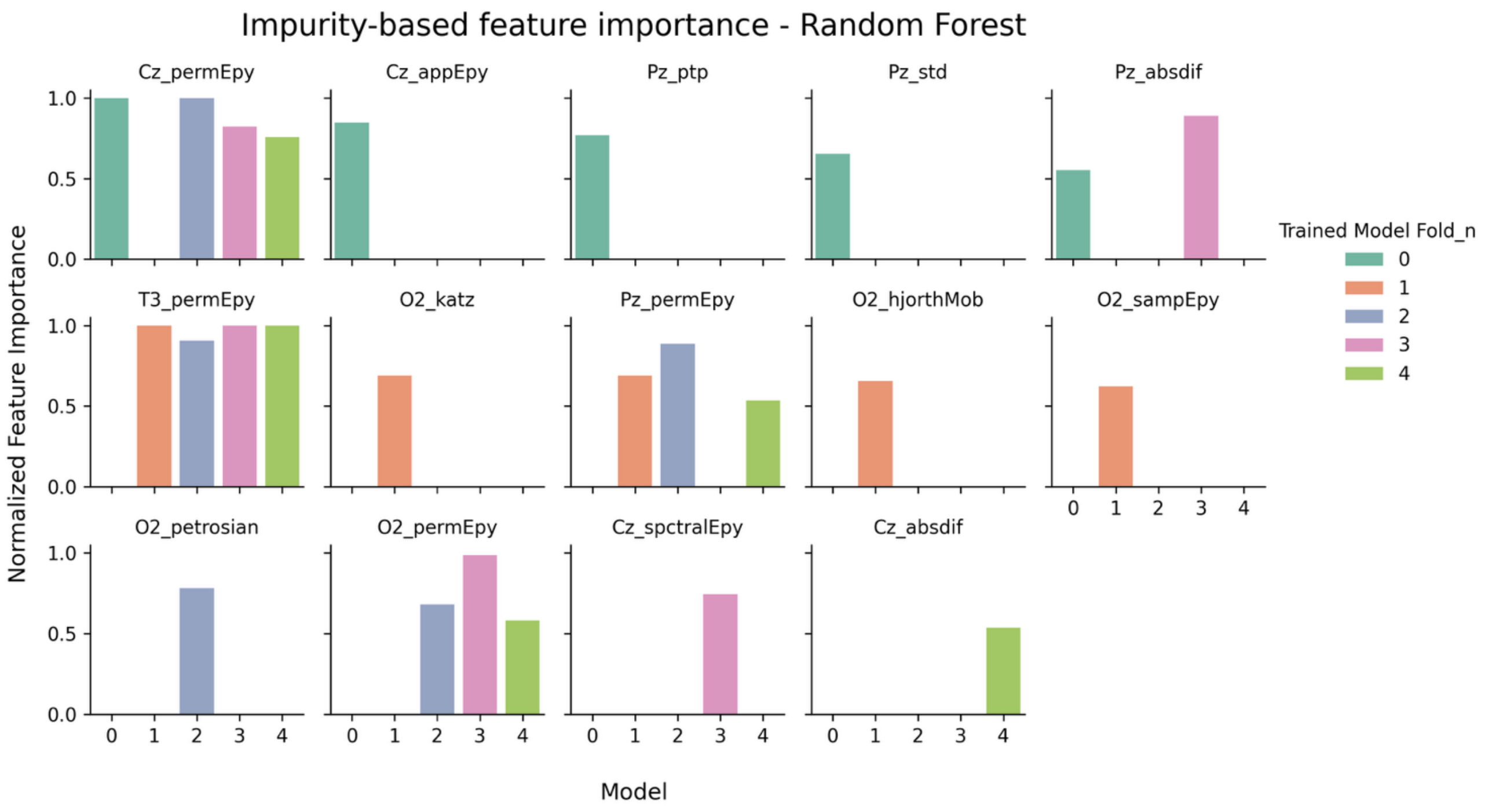

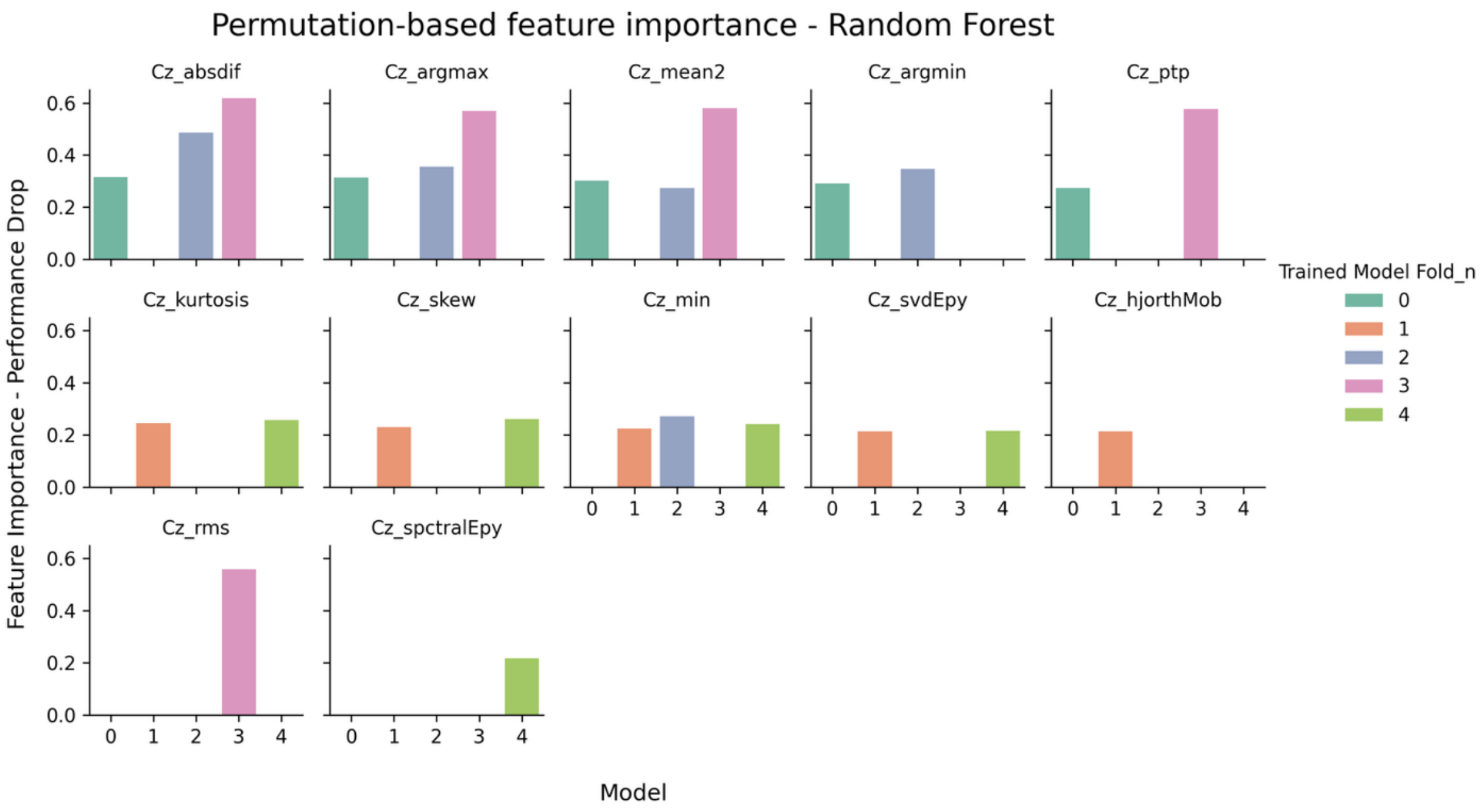

Time Domain – Resting – EEG | CP vs Control

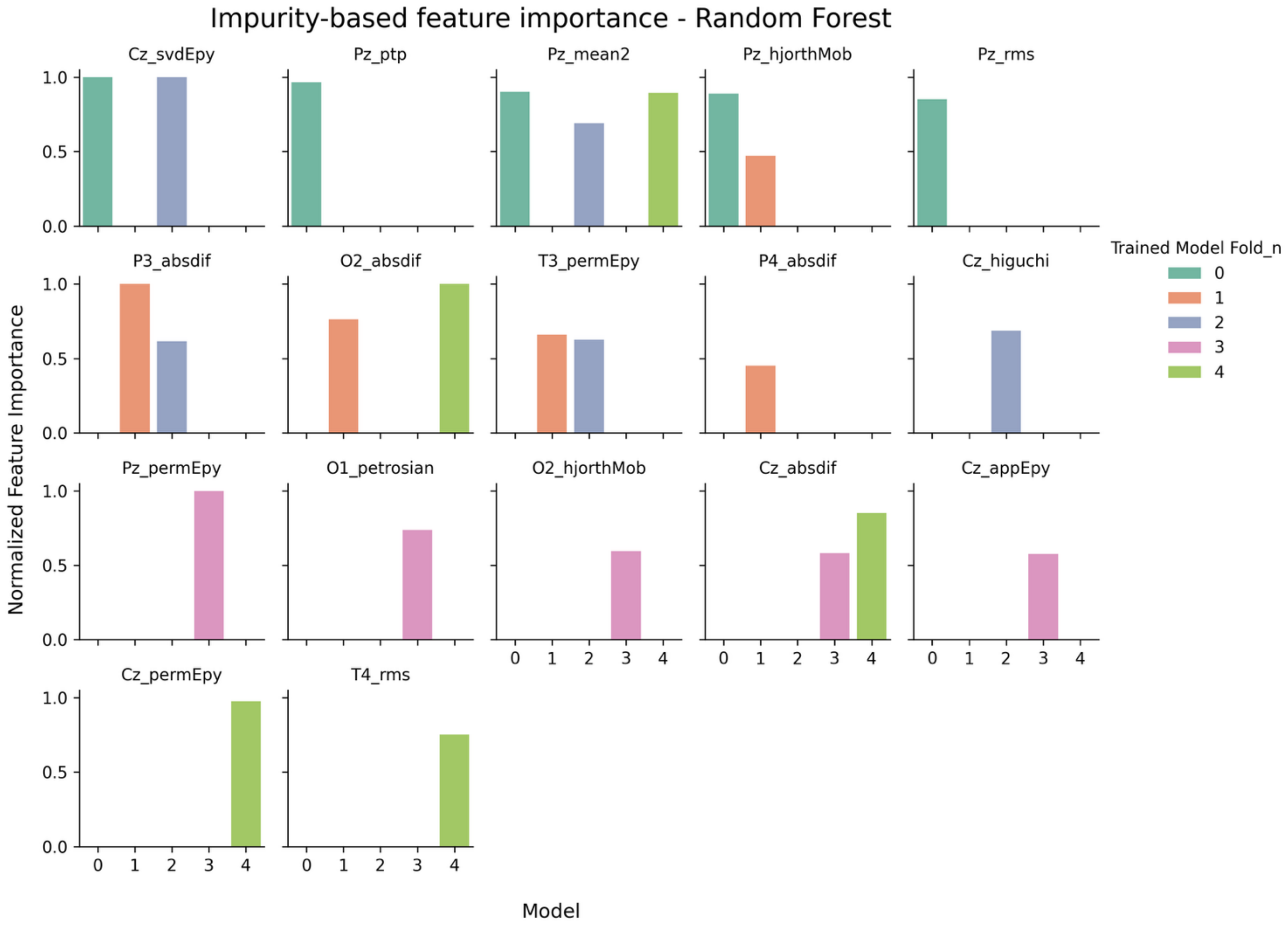

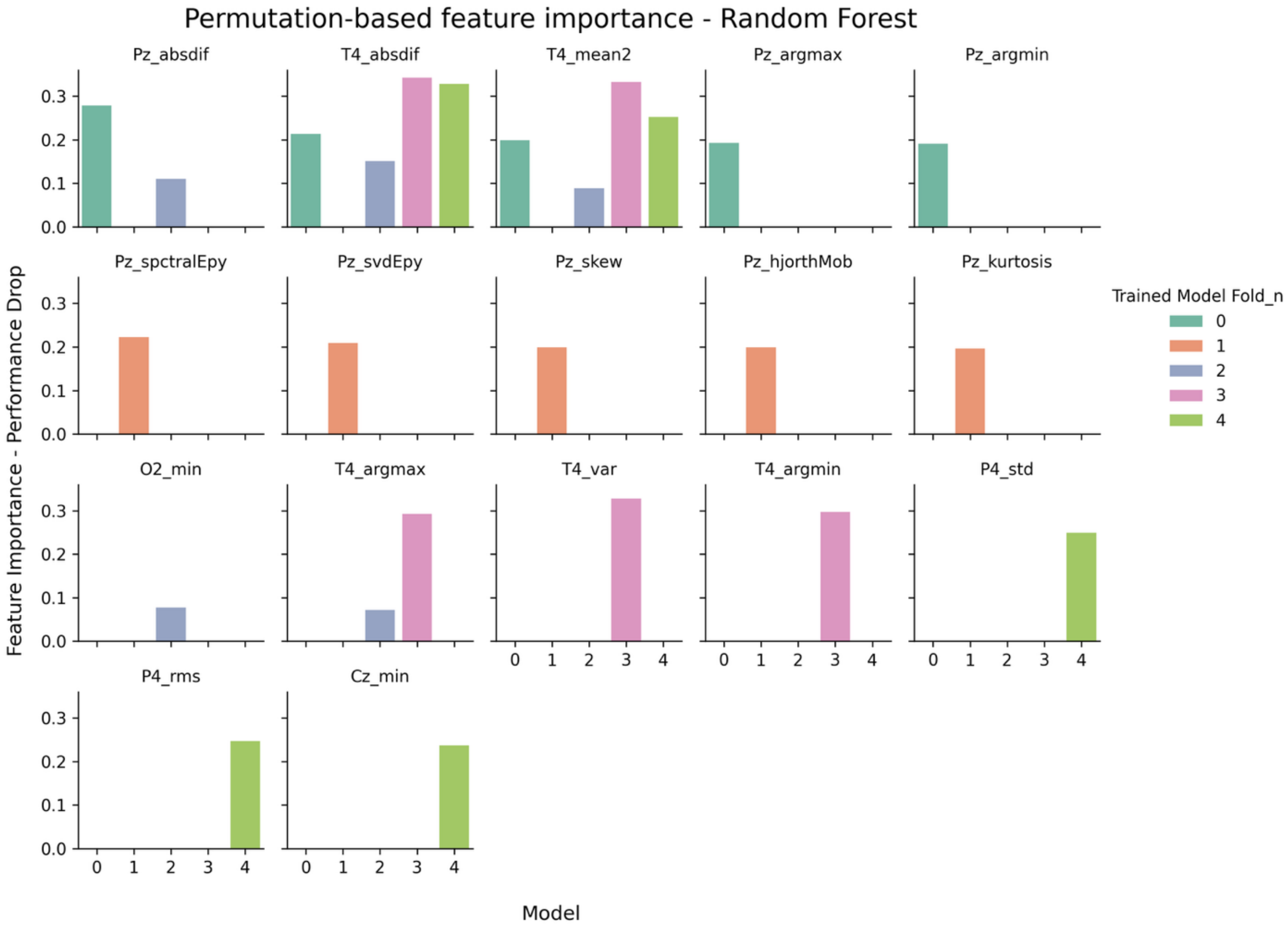

Freq Domain – Cold Pressor – EEG | CP vs Control

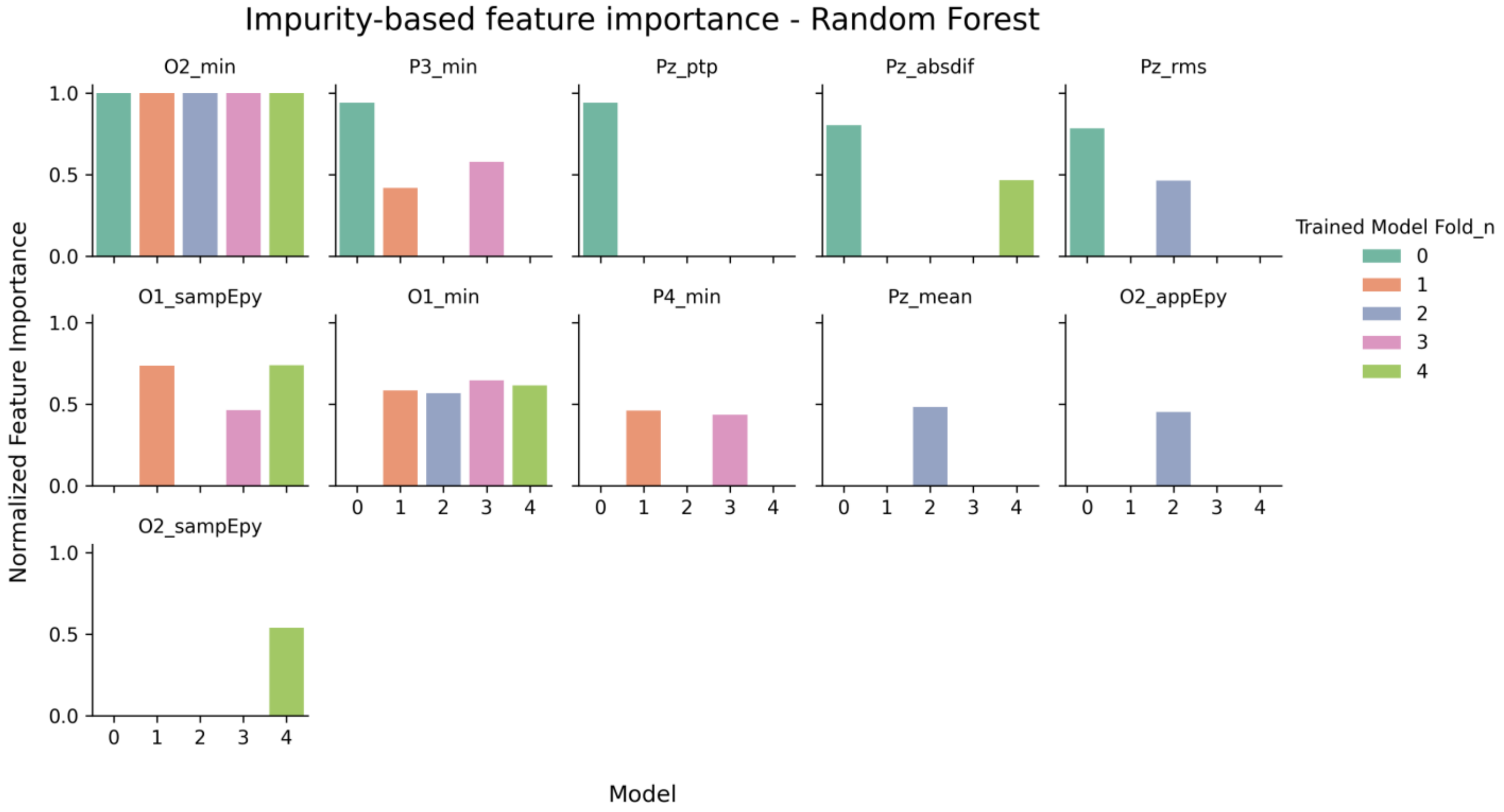

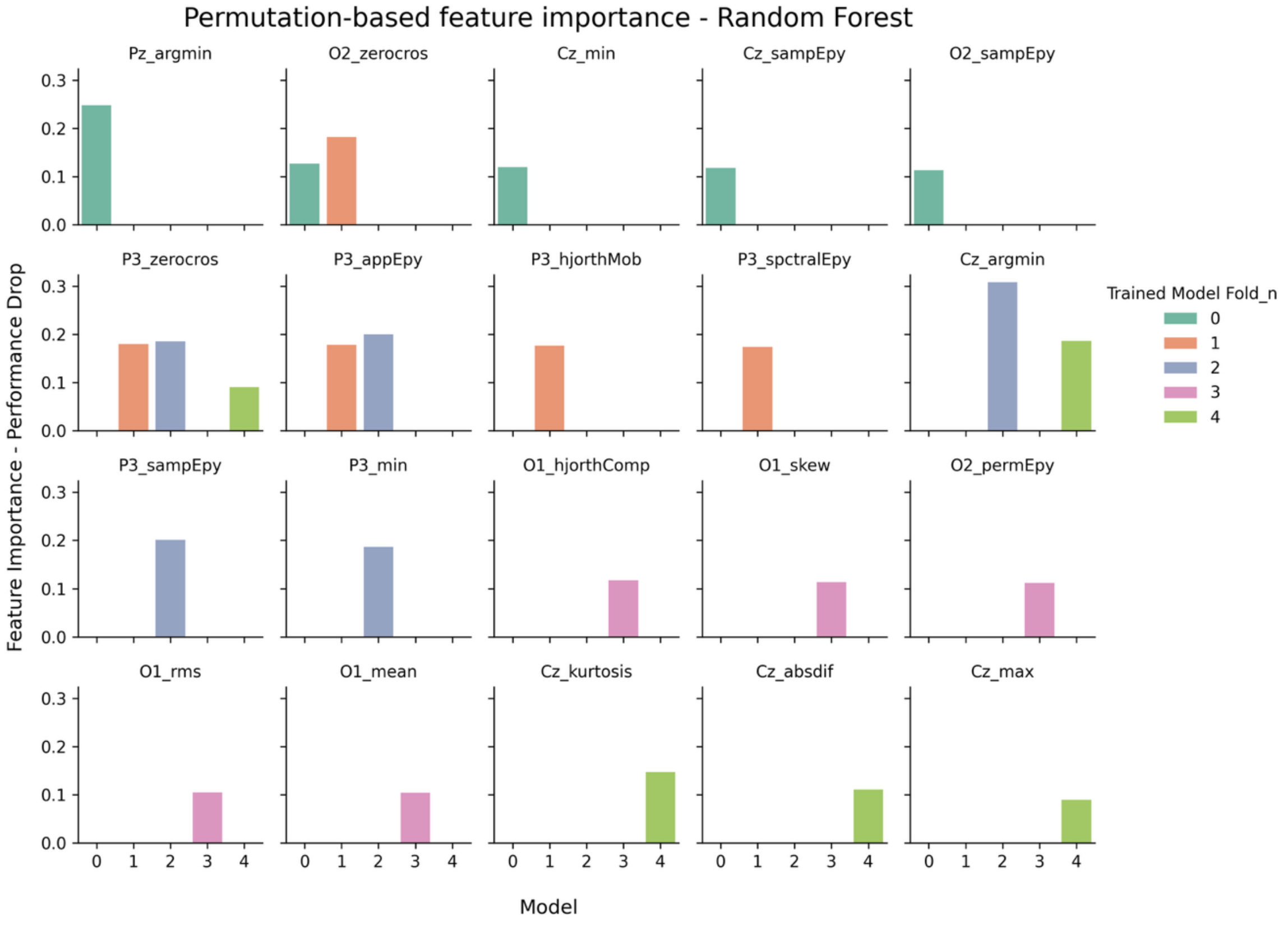

Freq Domain – Resting – EEG | CP vs Control

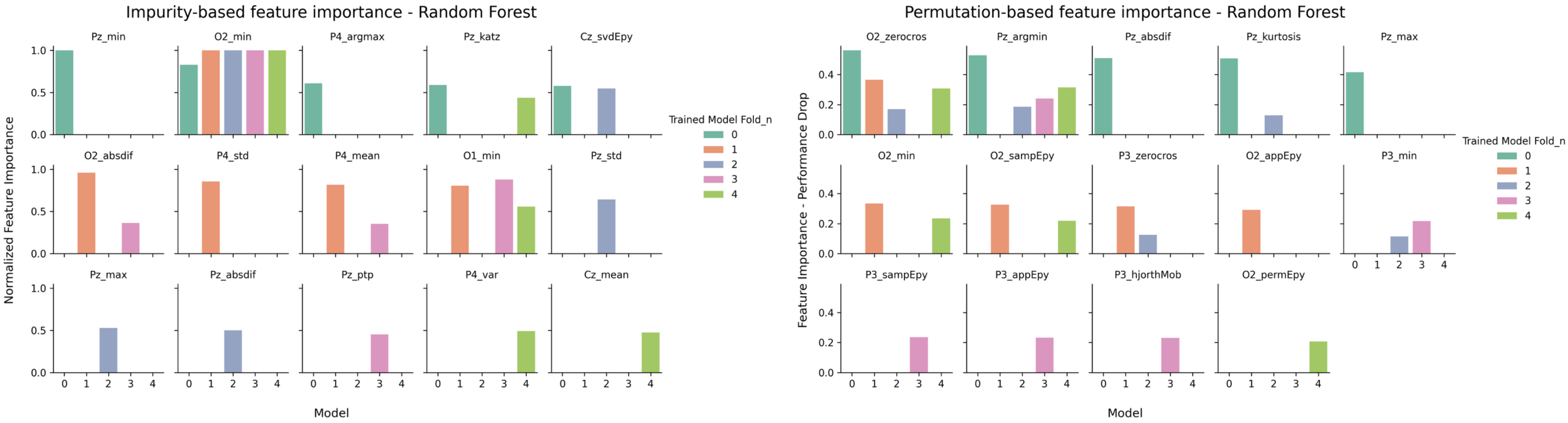

CP vs Control – EEG + ECG

Time Domain – Cold Pressor – EEG + ECG | CP vs Control

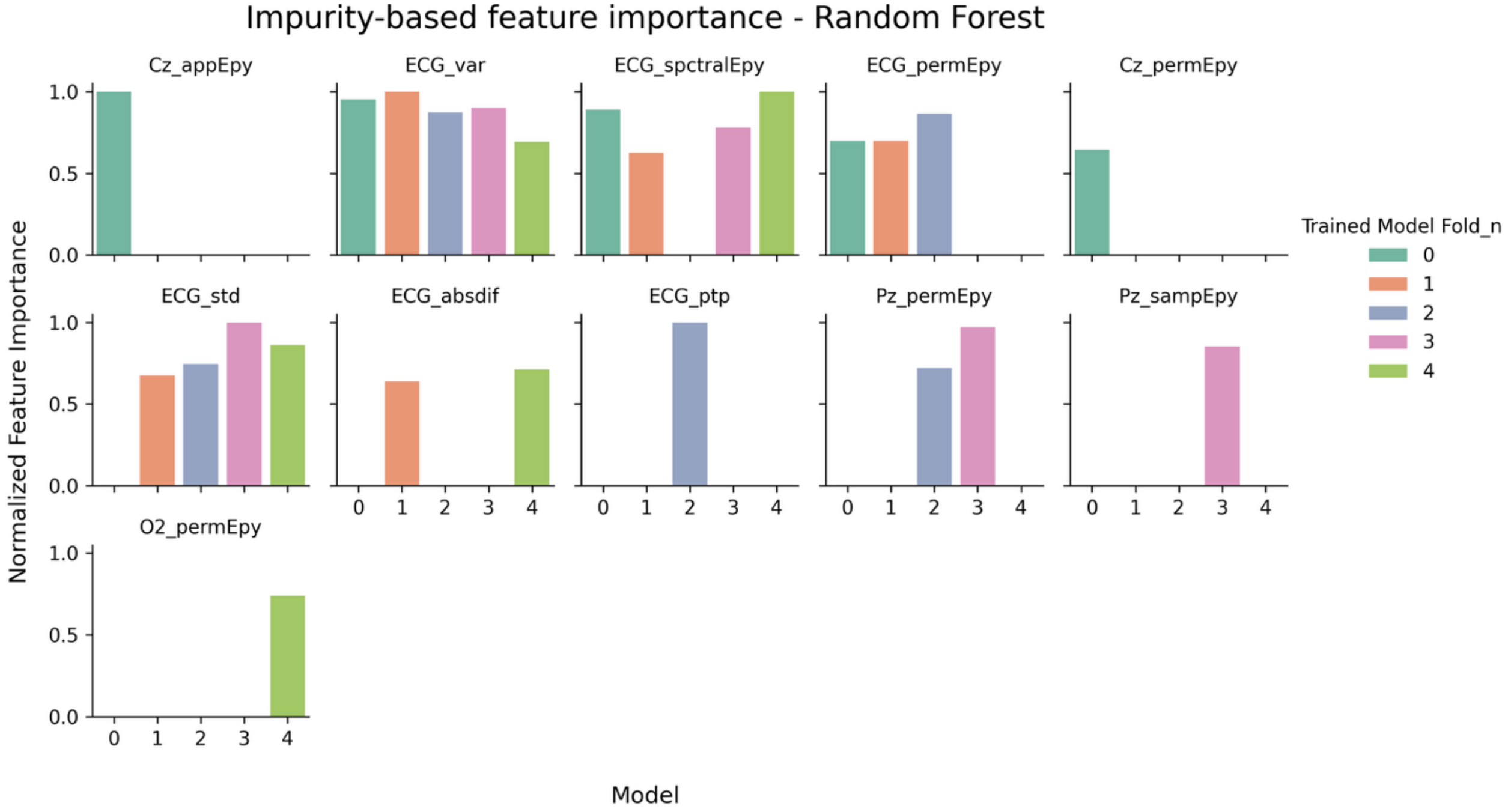

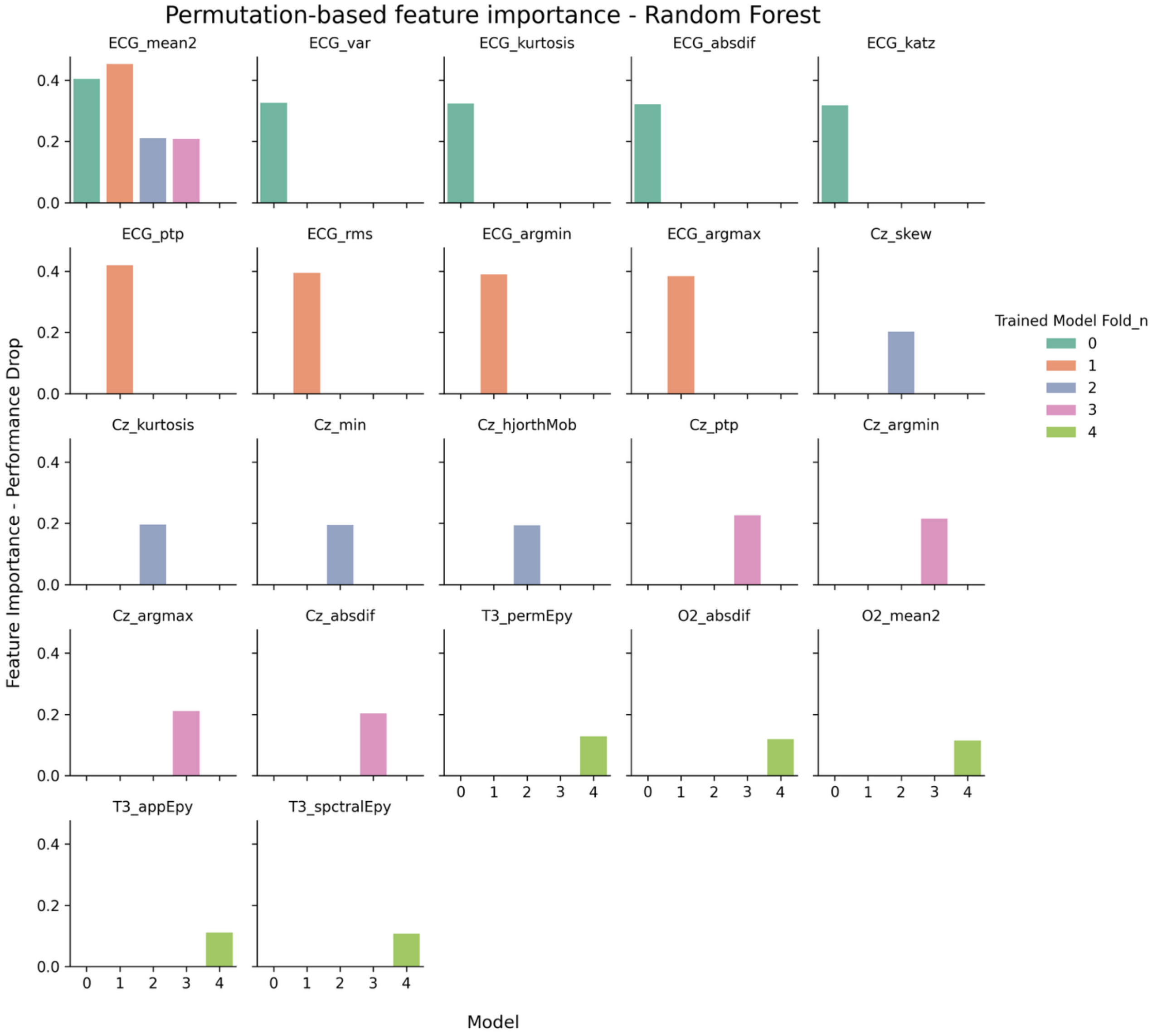

Time Domain – Resting – EEG + ECG | CP vs Control

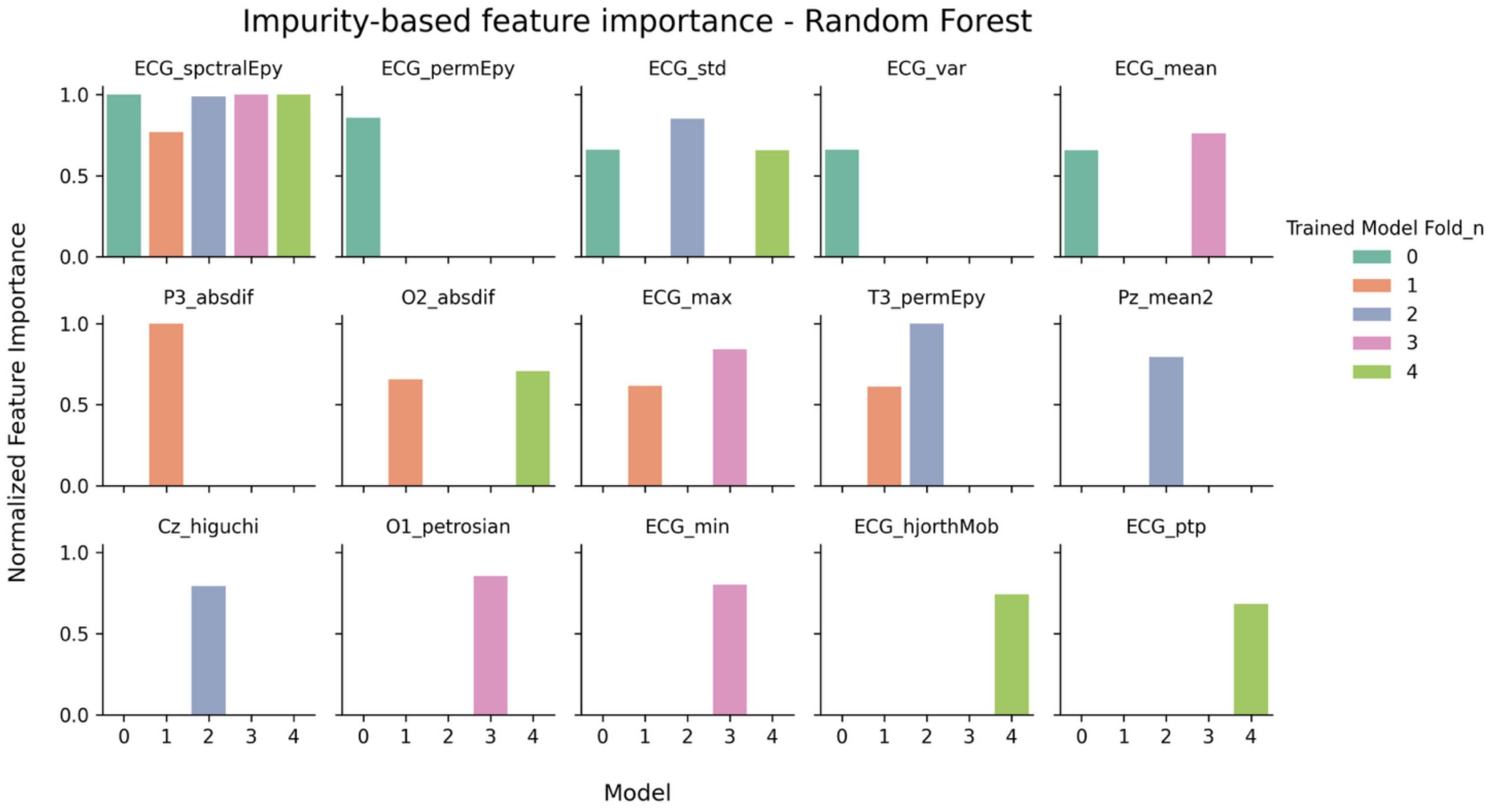

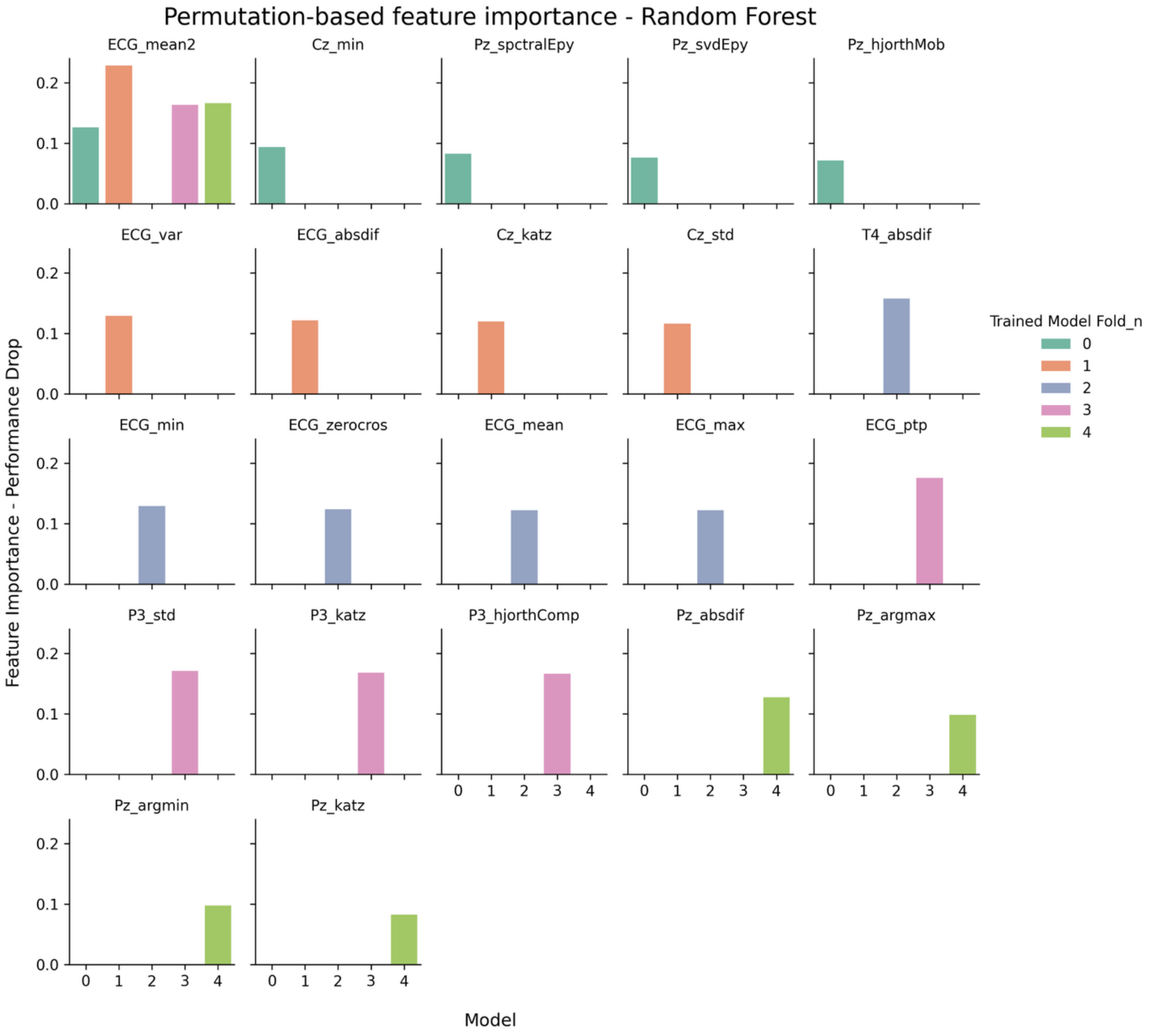

Freq Domain – Cold Pressor – EEG + ECG | CP vs Control

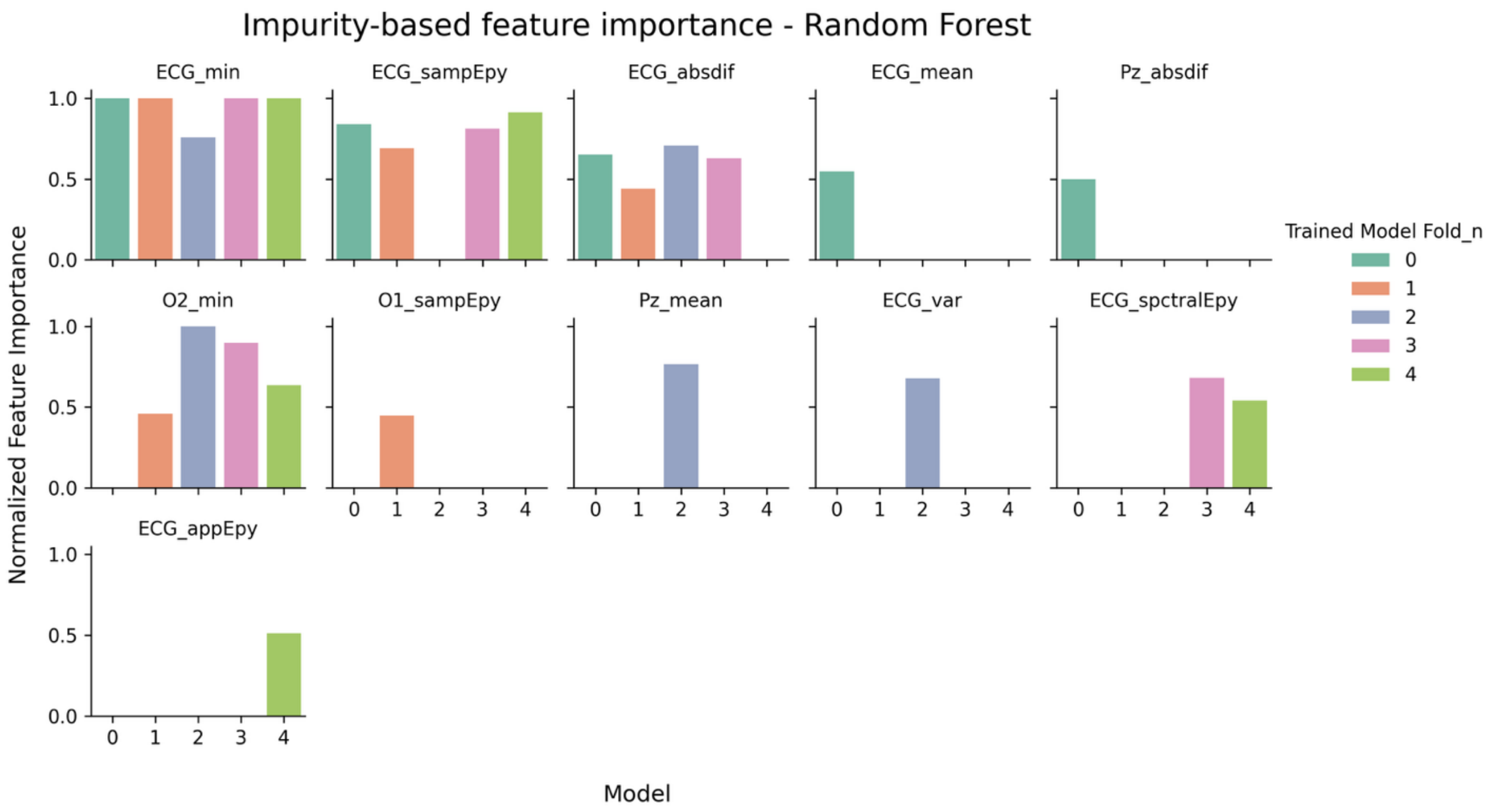

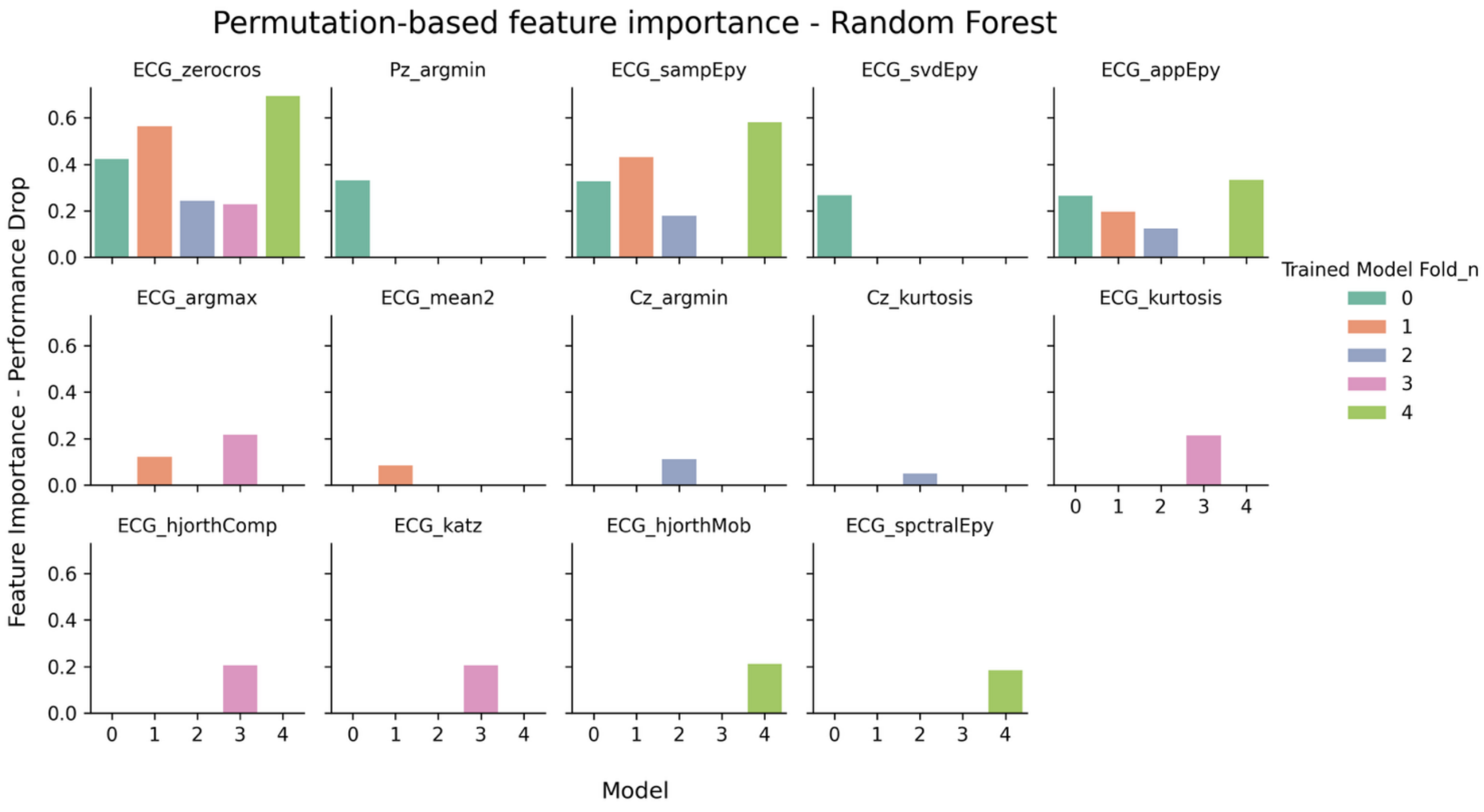

Freq Domain – Resting – EEG + ECG | CP vs Control

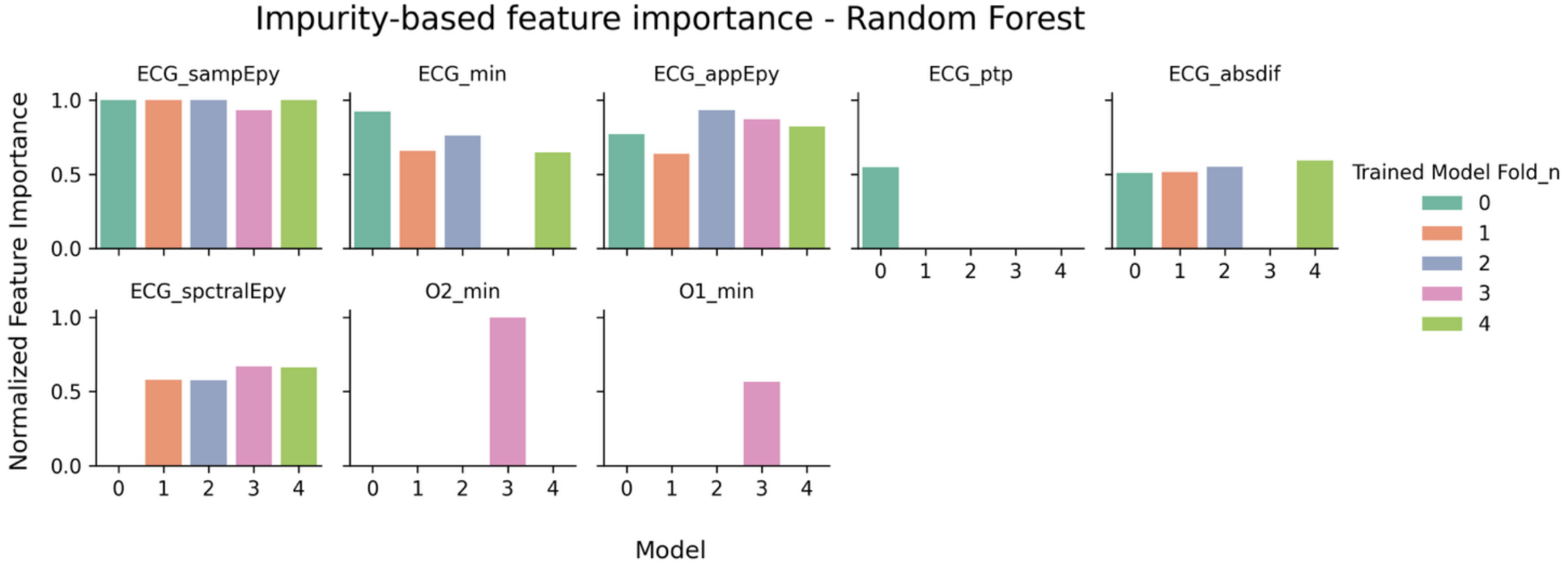

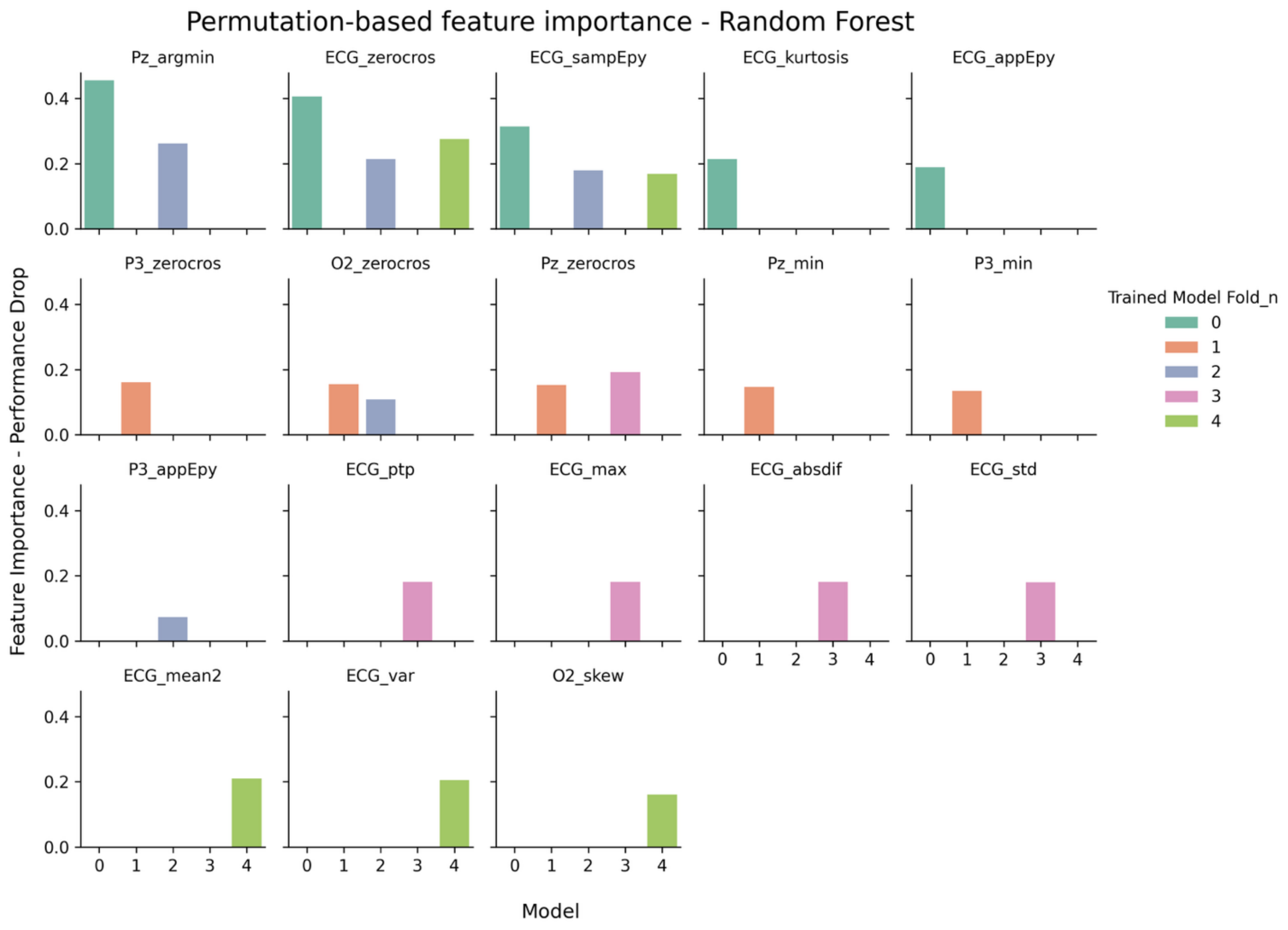

Painful vs Pain free – EEG

Time Domain – Cold Pressor – EEG | Pain vs Pain free

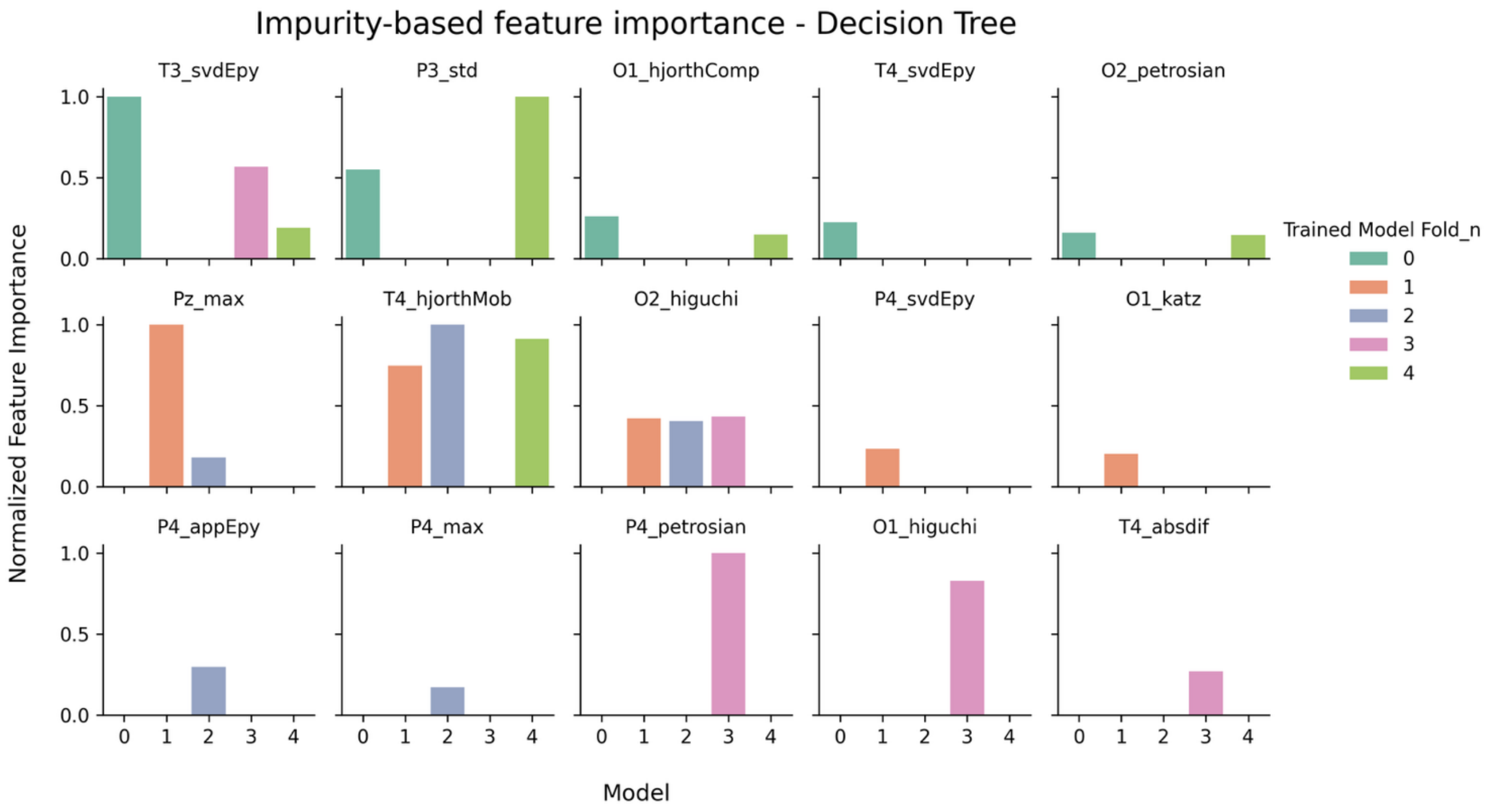

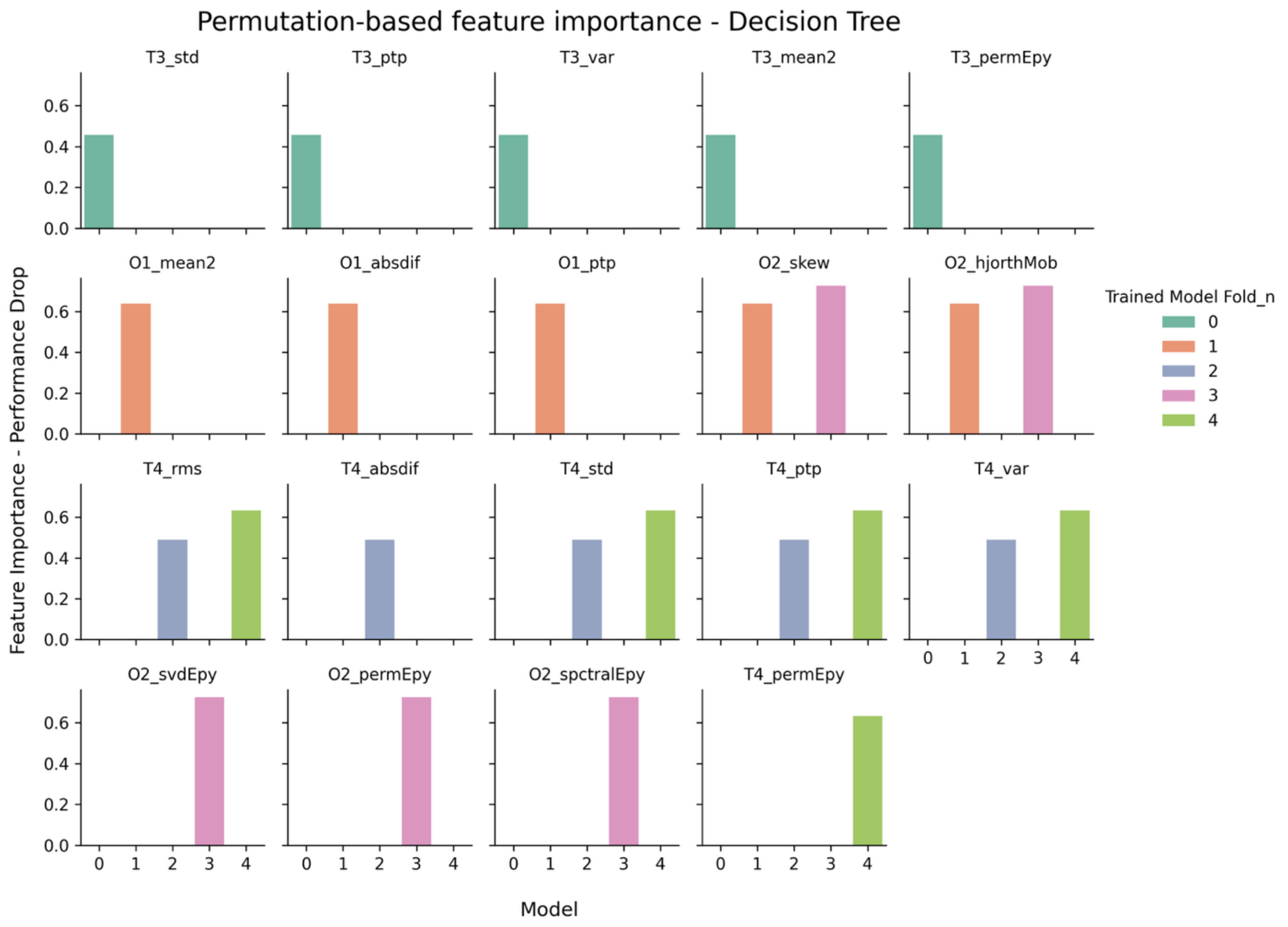

Time Domain – Resting – EEG | Pain vs Pain free

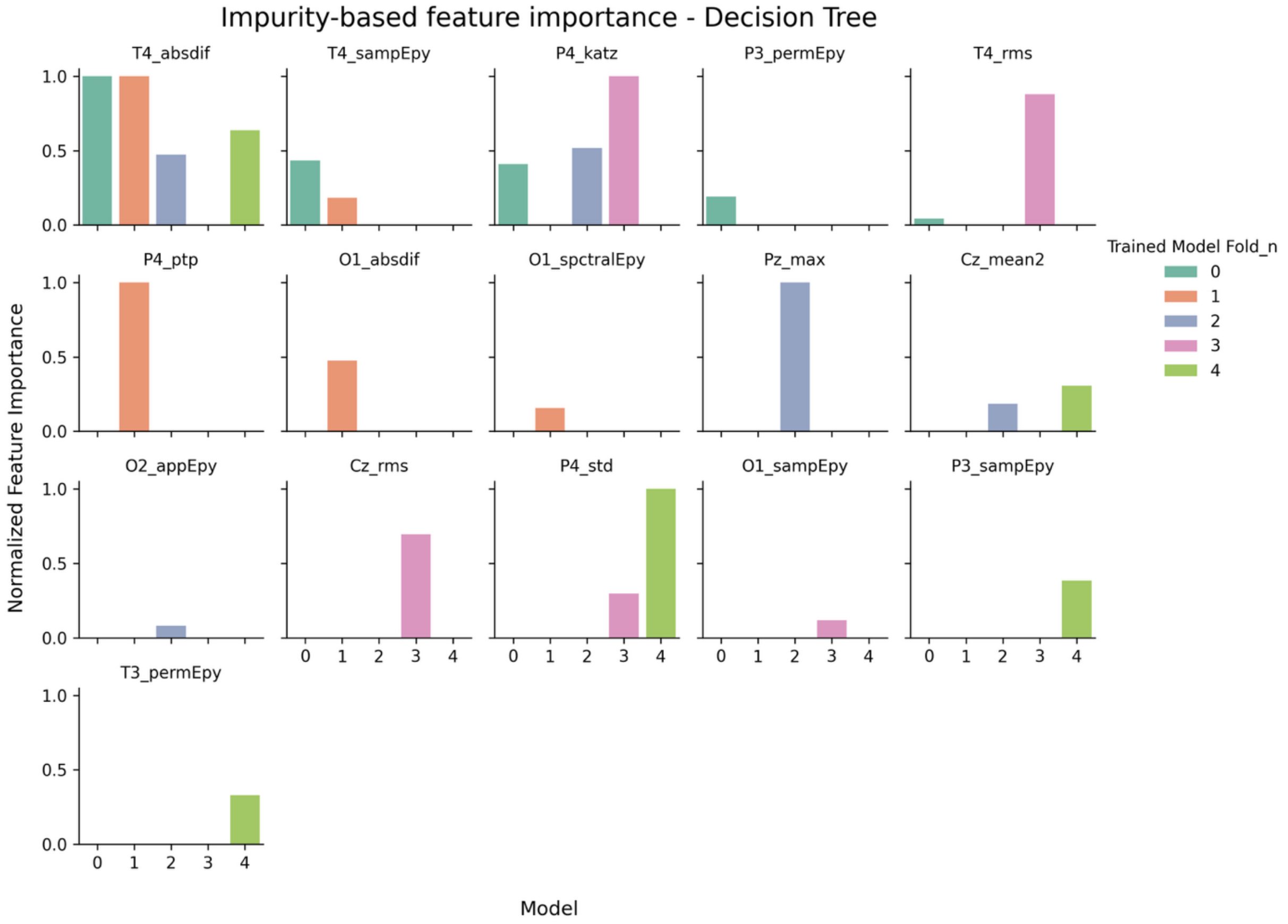

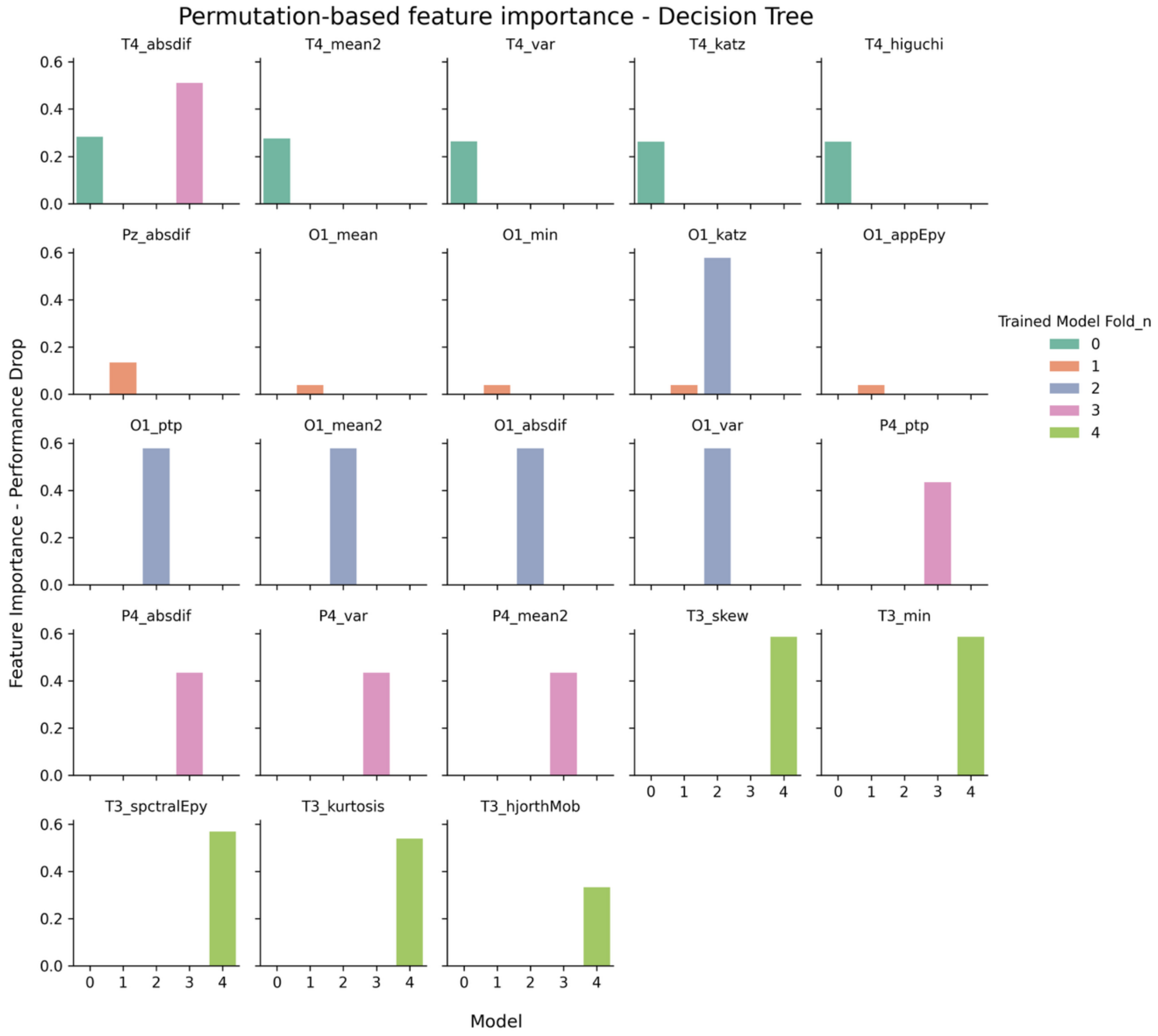

Freq Domain – Cold Pressor – EEG | Pain vs Pain free

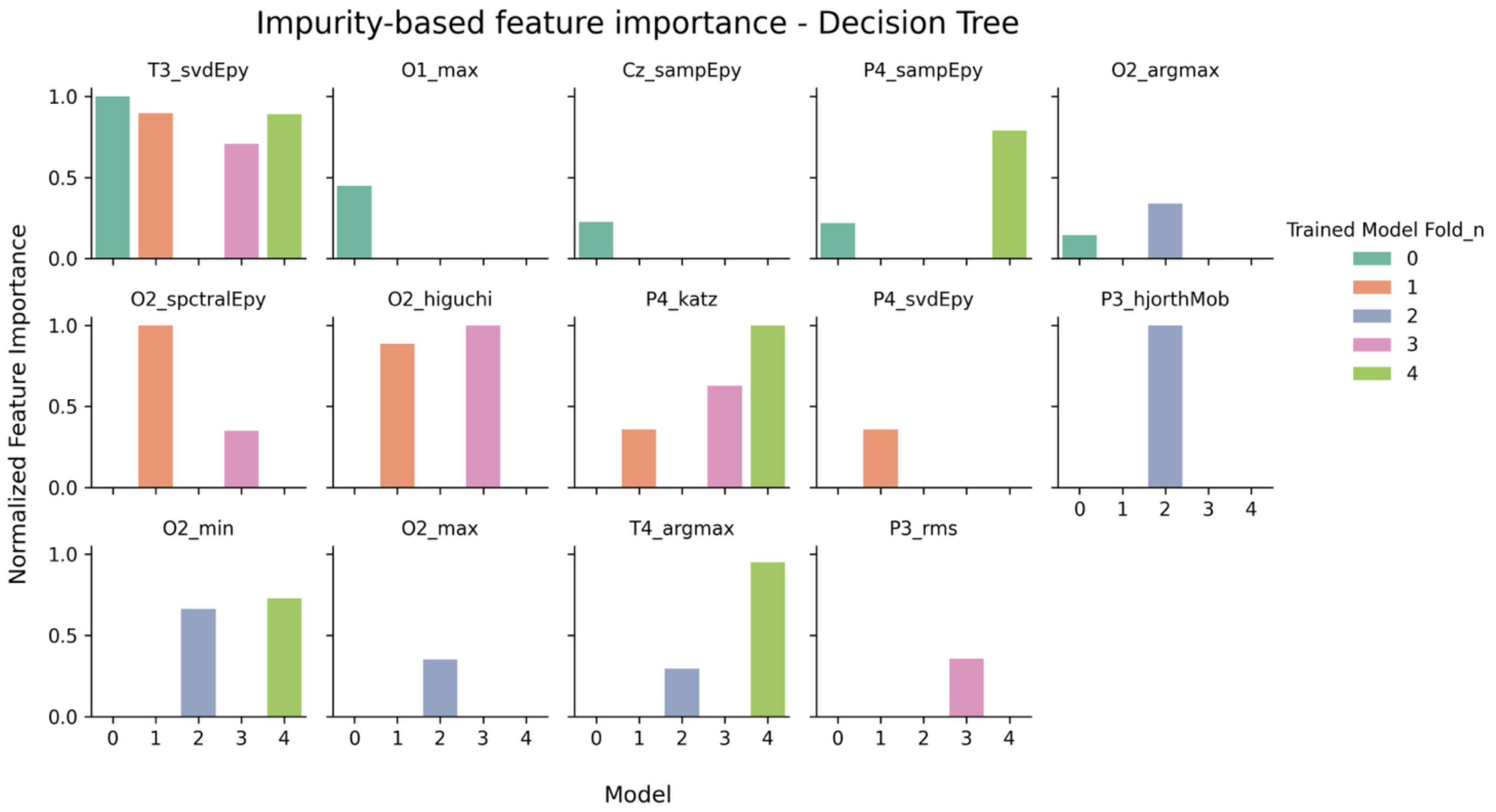

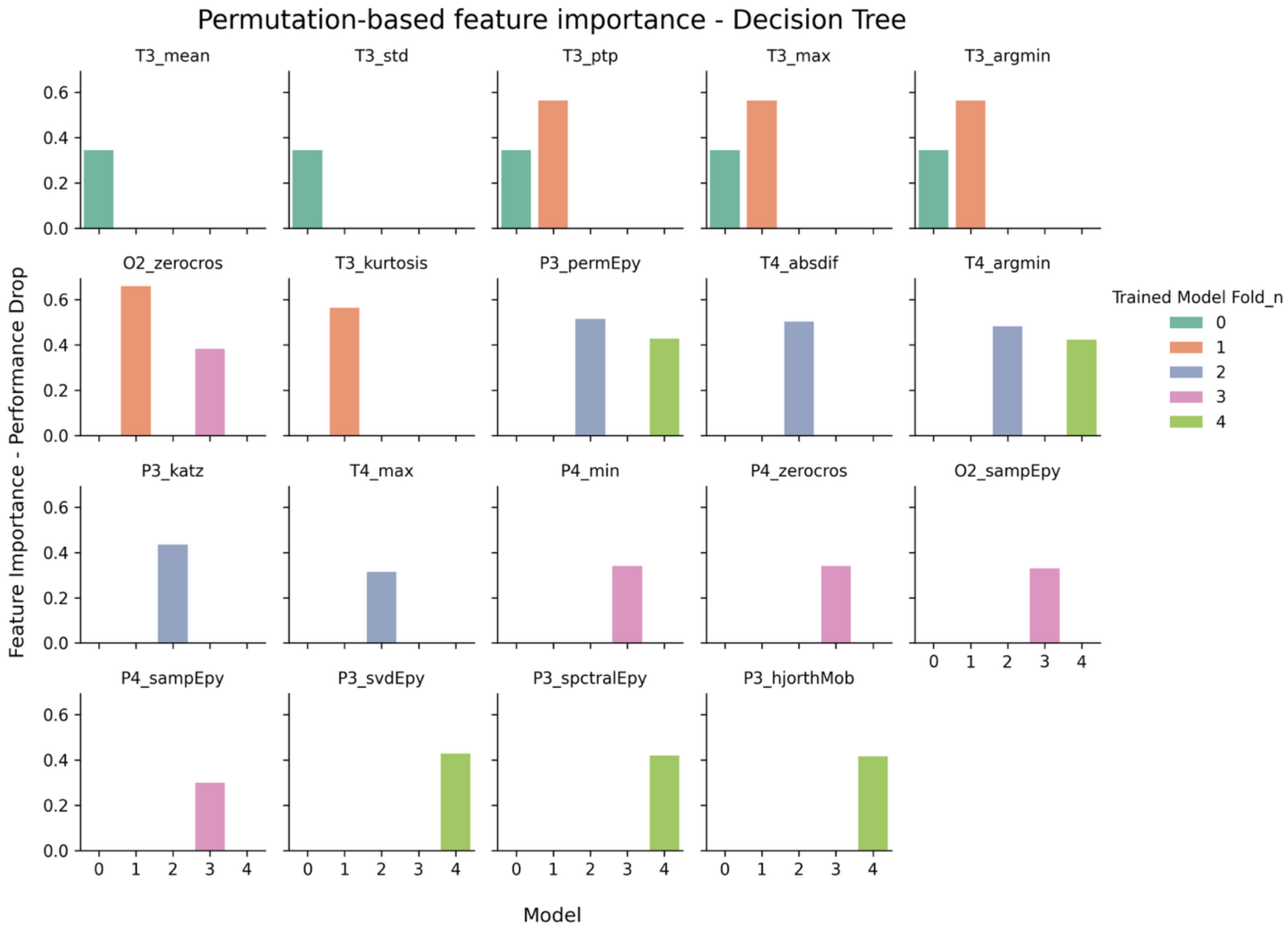

Freq Domain – Resting – EEG | Pain vs Pain free

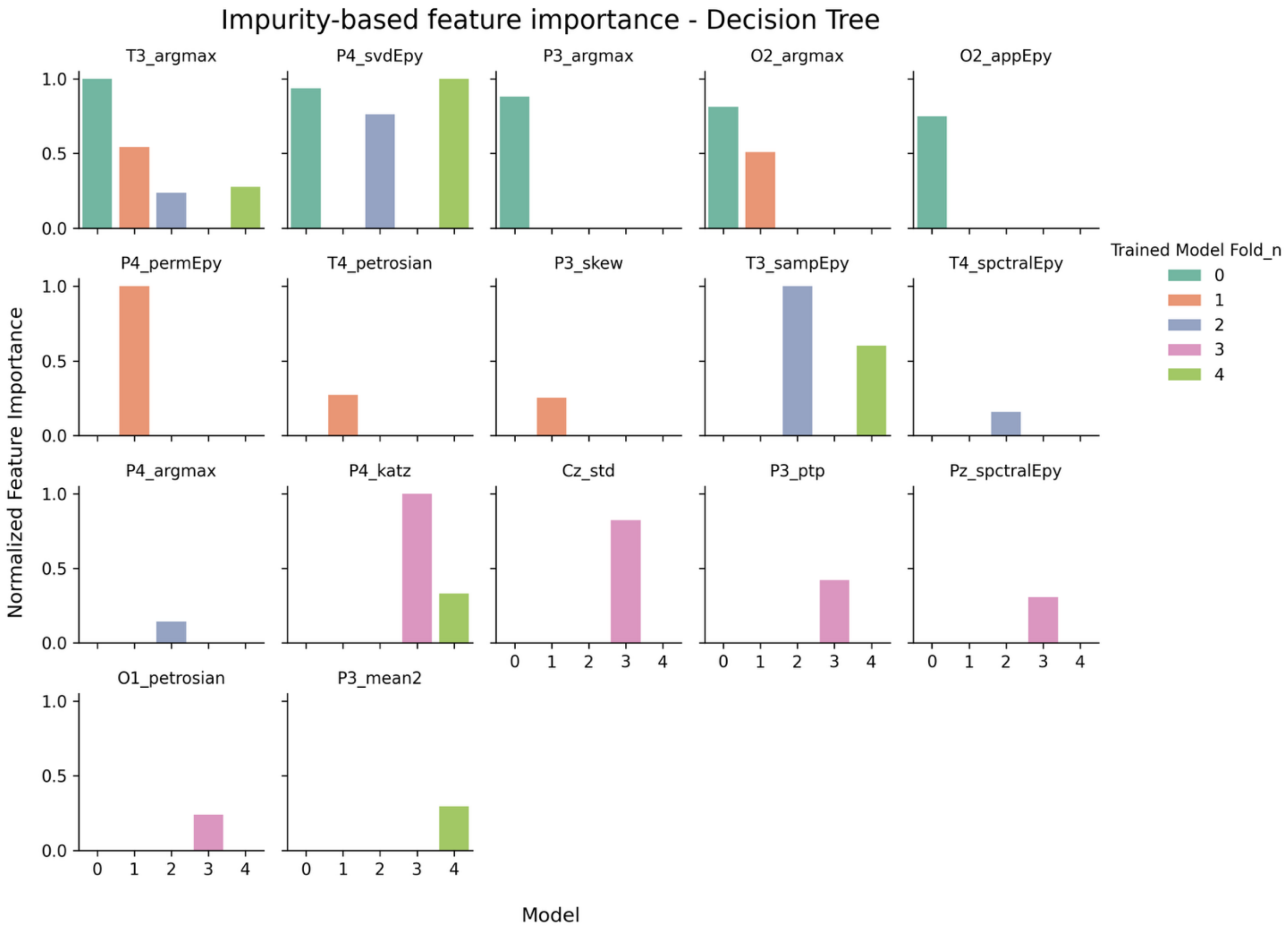

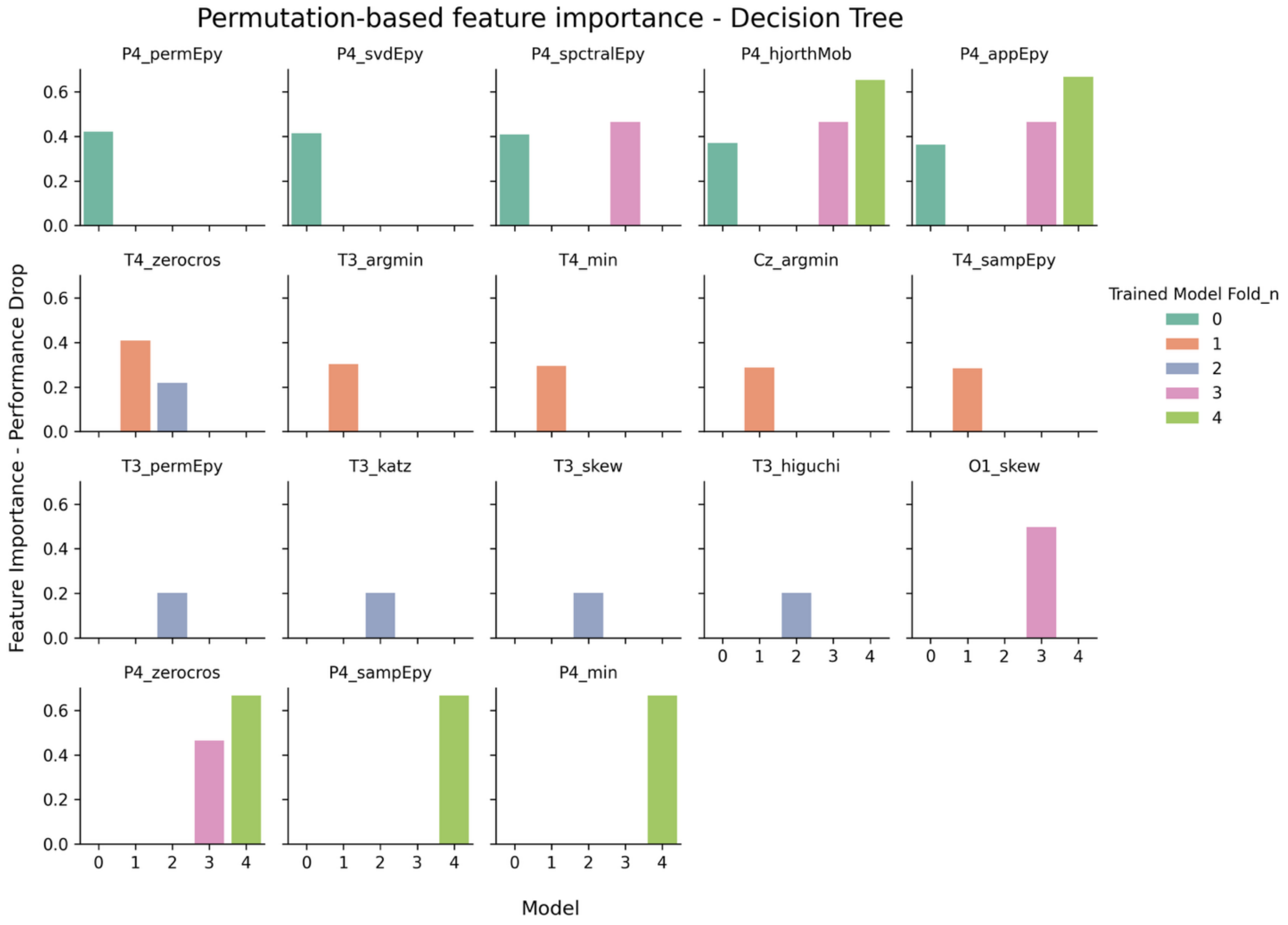

Painful vs Pain free – EEG + ECG

Time Domain – Cold Pressor – EEG+ECG | Pain vs Pain free

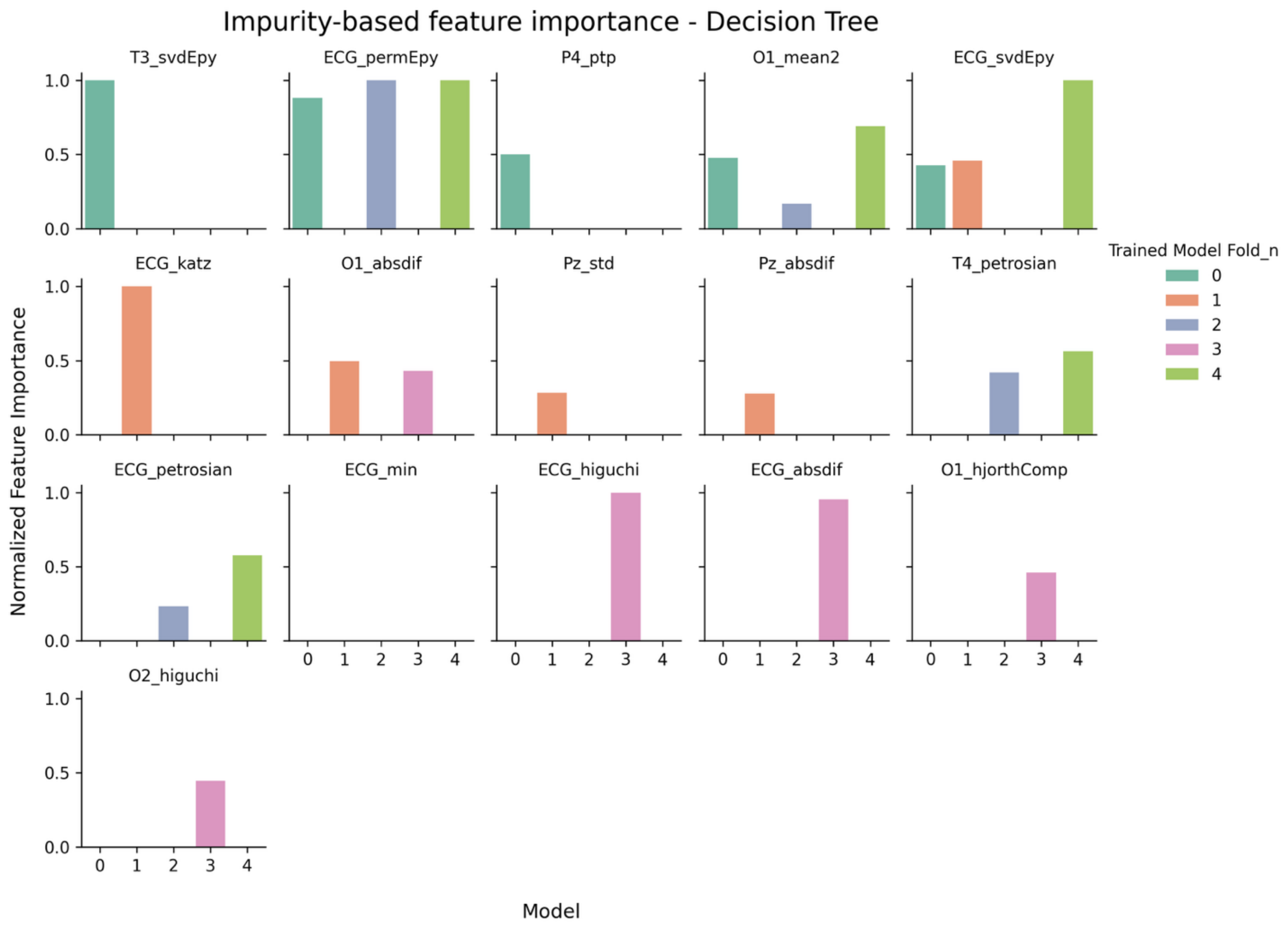

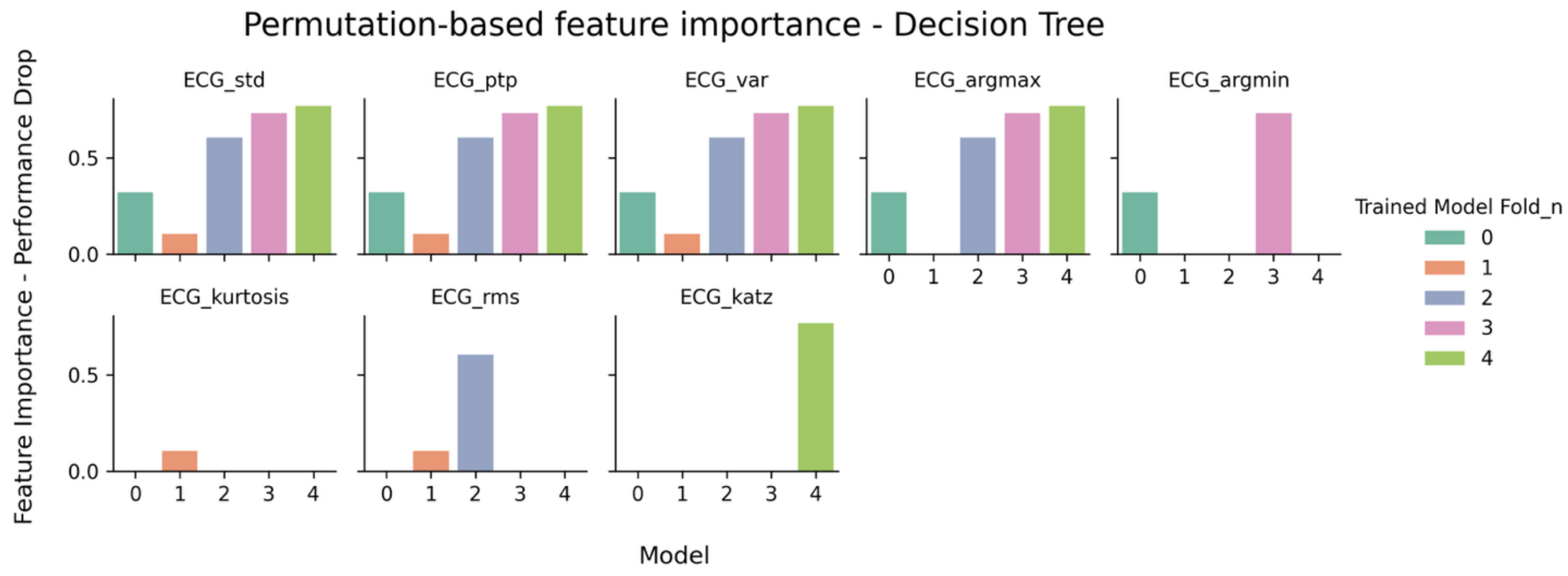

Time Domain – Resting – EEG+ECG | Pain vs Pain free

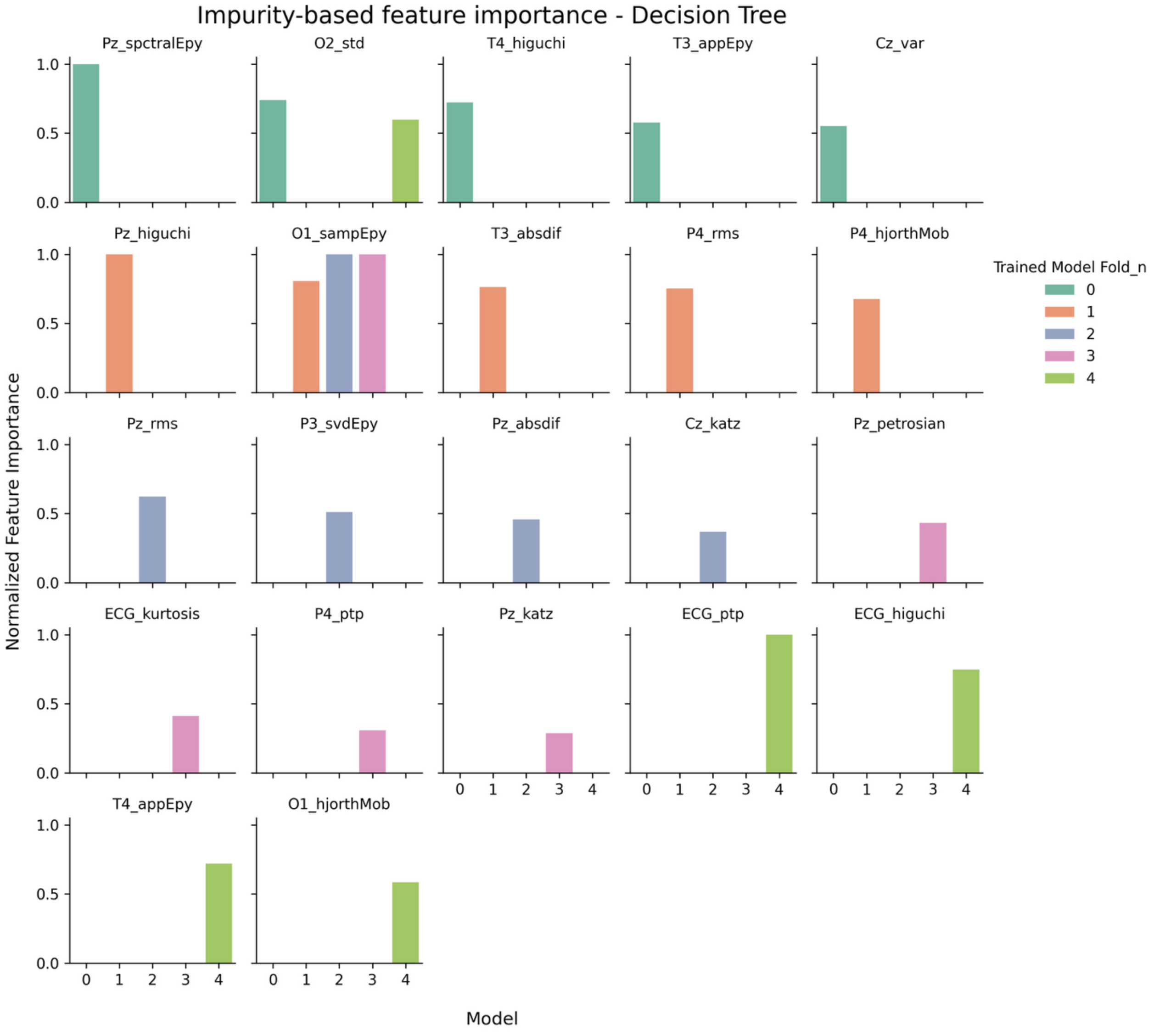

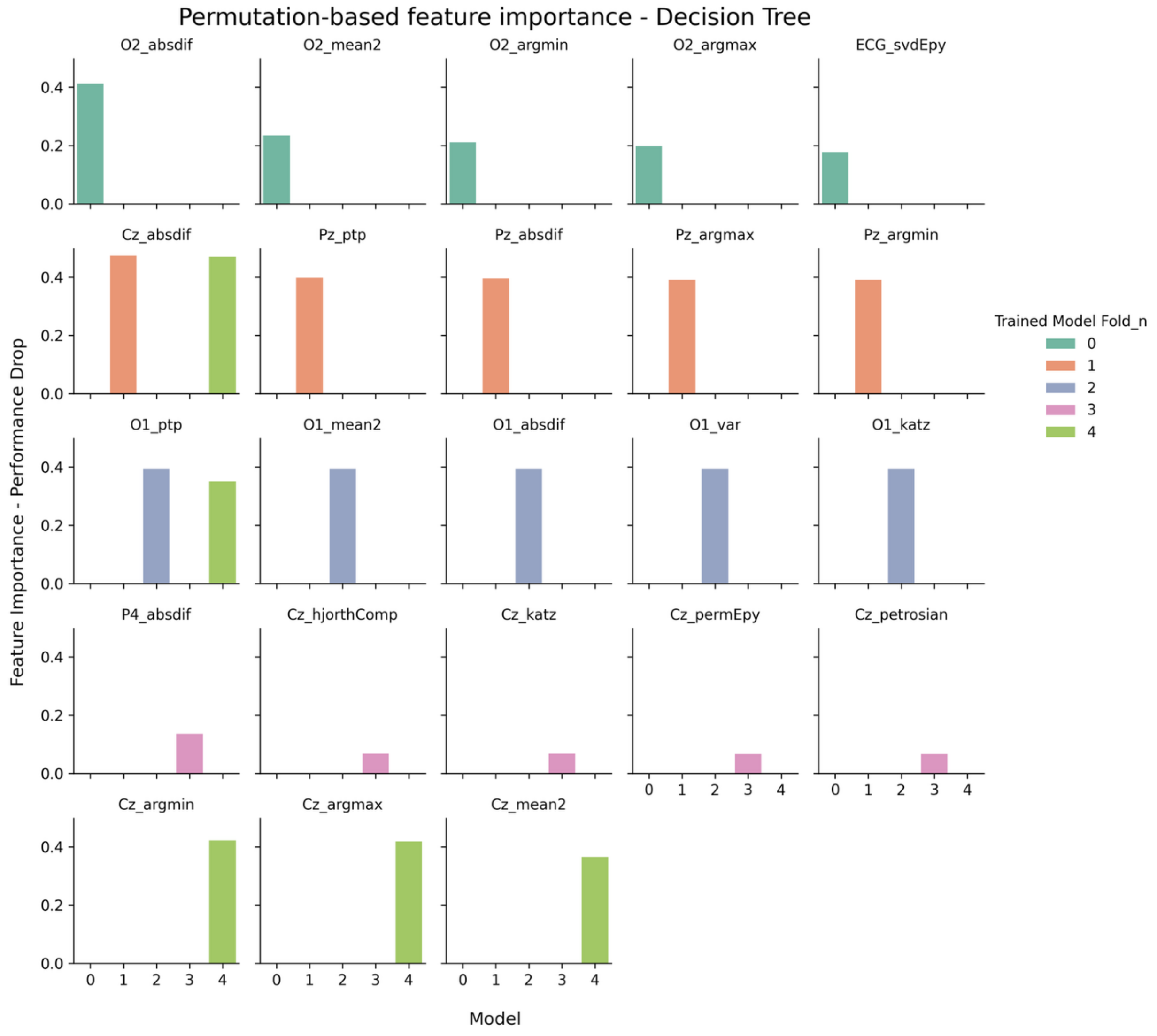

Freq Domain – Cold Pressor – EEG+ECG | Pain vs Pain free

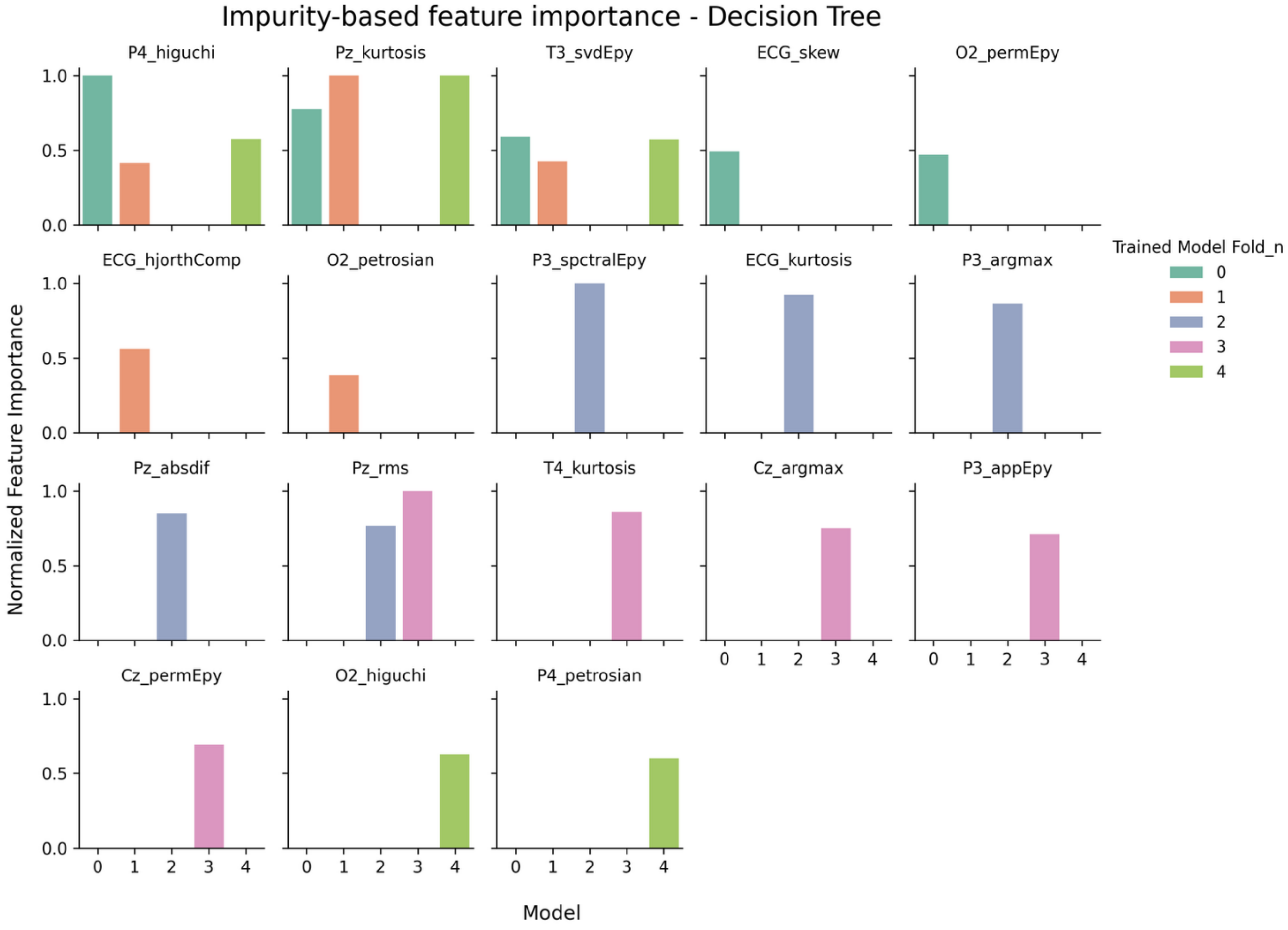

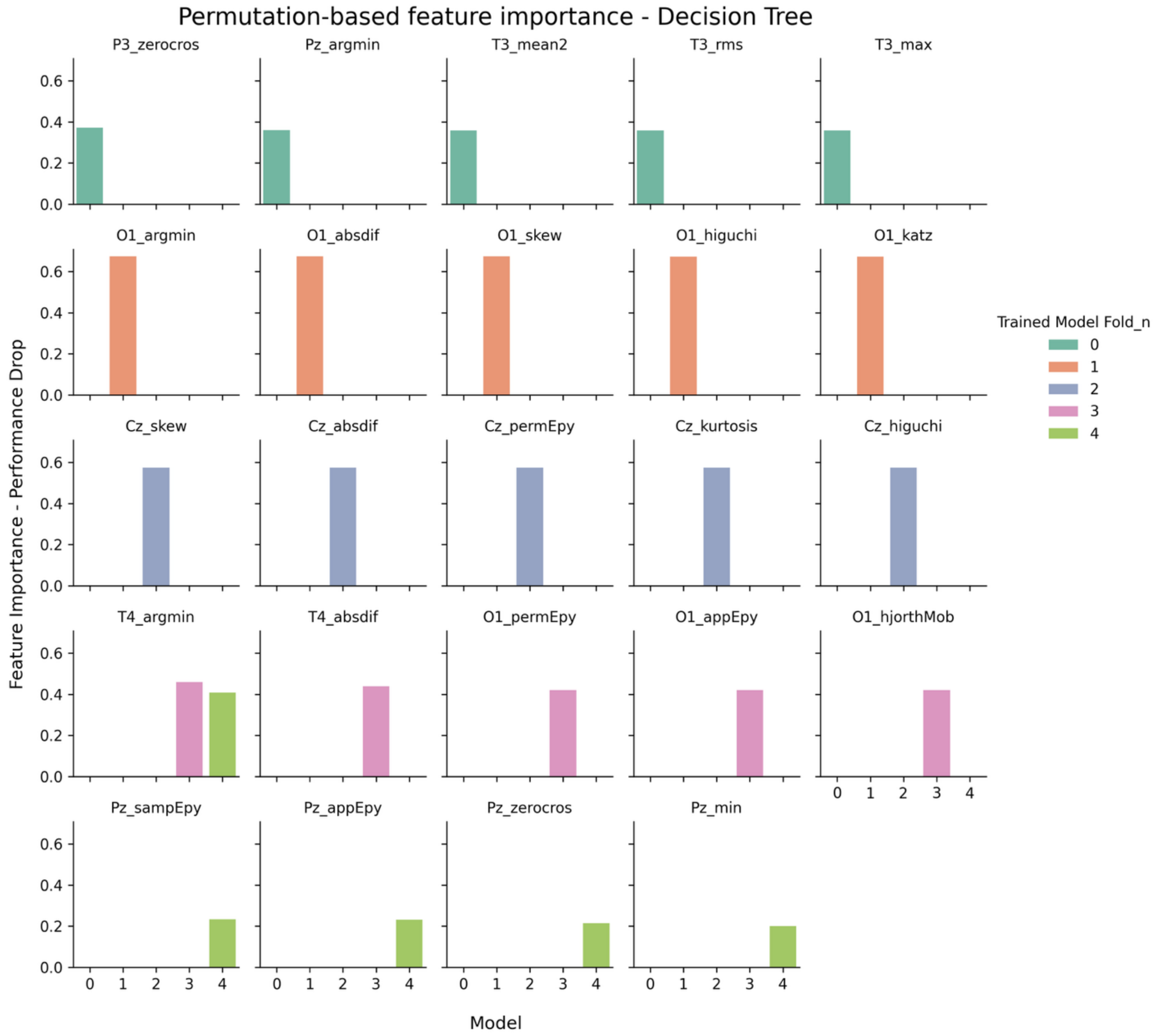

Freq Domain – Resting – EEG+ECG | Pain vs Pain free

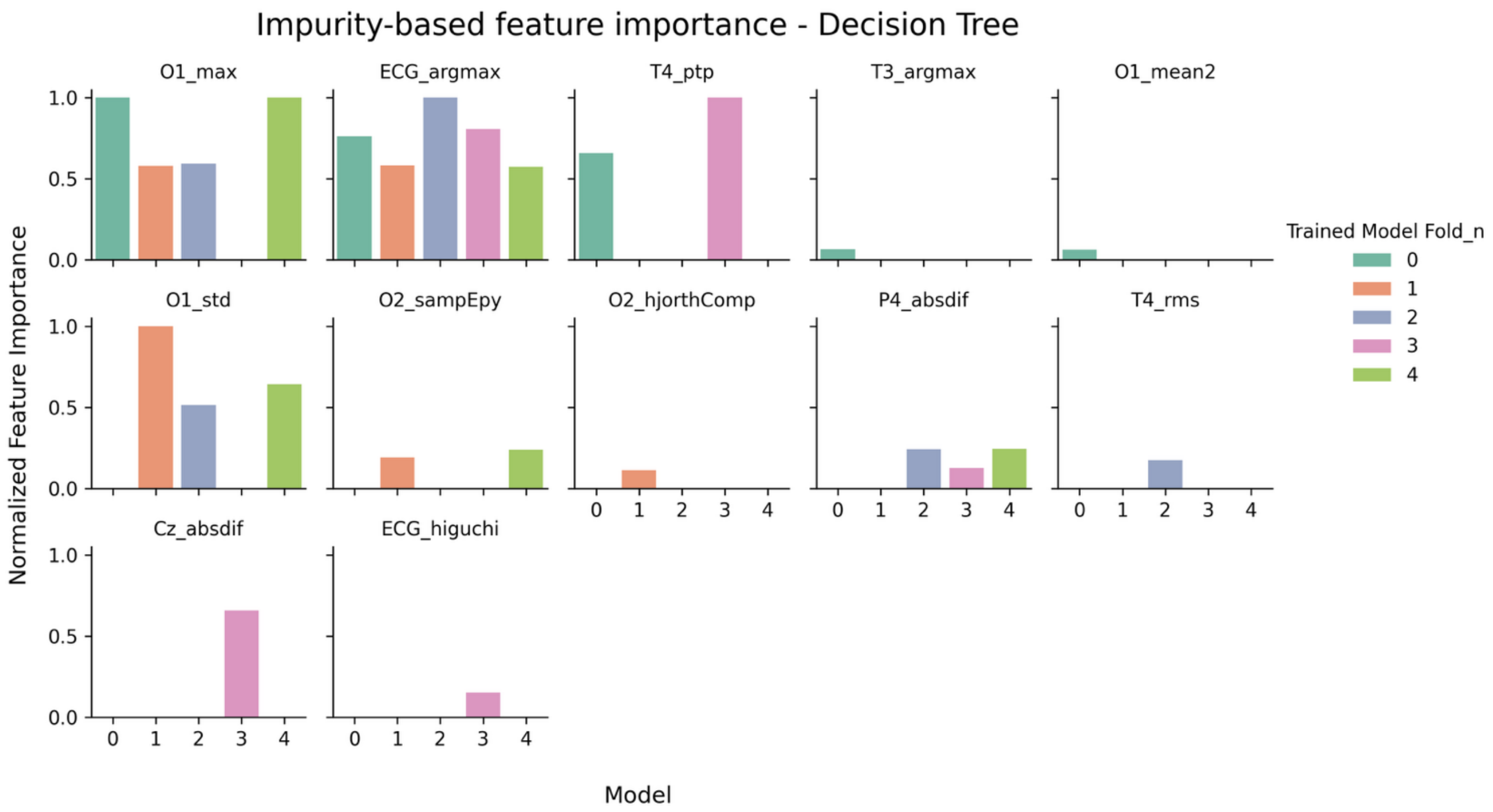

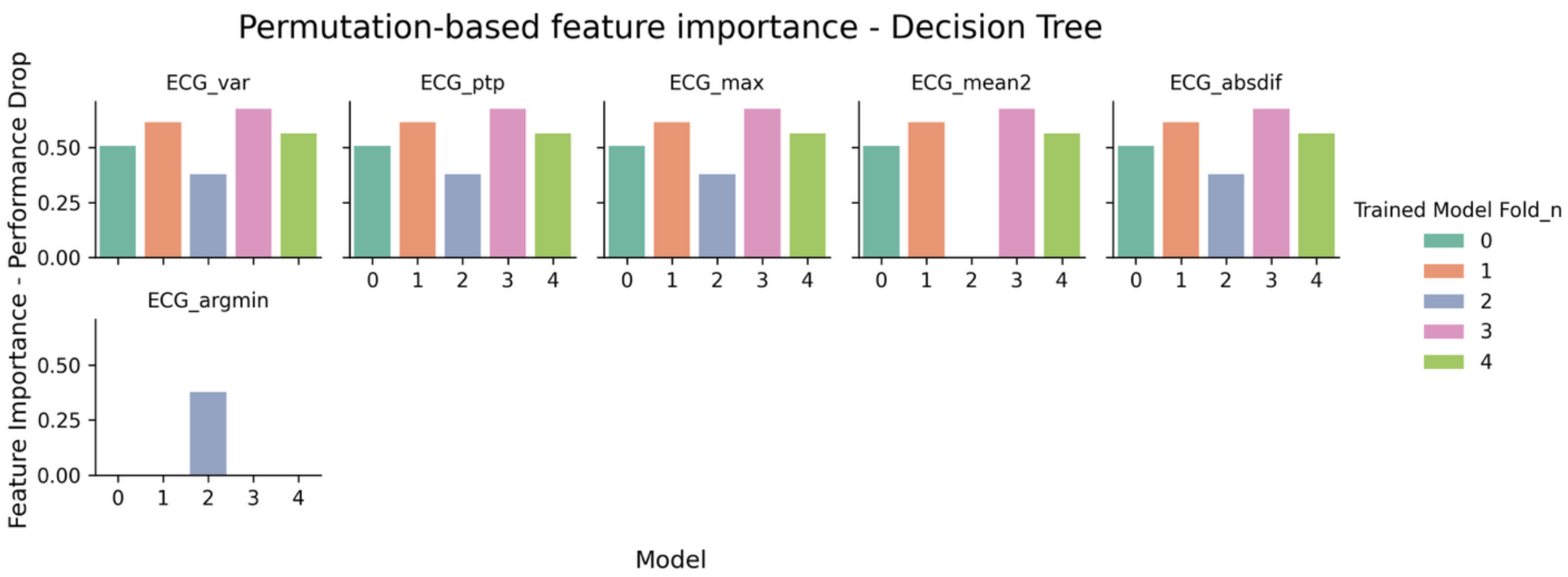

Supplement: Supplementary file 4 [file Datasheet4.pdf]
