# Supplementary material for: Electrophysiological biomarkers for foundational signal markers in chronic pancreatitis using machine learning models
Source: Front Pain Res (Lausanne). 2026 Jul 22;7:1857591. doi: 10.3389/fpain.2026.1857591 (PMC13438447; doi:10.3389/fpain.2026.1857591)
Supplement: Supplementary file 5 [file Datasheet5.pdf]

# Supplementary — Software Environment

*Manuscript 1857591 — Python package versions used for the analysis (requirements.txt)*

```
# Python 3.8.8
numpy==1.24.4
pandas==1.5.3
scikit-learn==1.3.2
scipy==1.10.1
matplotlib==3.7.0
seaborn==0.13.1
mne==1.3.1
imbalanced-learn==0.12.2
antropy==0.1.5
```
